# Reference Standards in Artificial Intelligence Detection of Apical Periodontitis: A Narrative Review of What Reported Accuracy Measures

**DOI:** 10.3390/diagnostics16193225

**Published:** 2026-10-06

**Authors:** Omar R. Alkhattab, Abdullah Bokhary

**Affiliations:** 1 Department of Endodontics, Faculty of Dentistry, King Abdulaziz University, Jeddah 21589, Saudi Arabia; oralkhattab@kau.edu.sa; 2 Department of Dental Public Health, Faculty of Dentistry, King Abdulaziz University, Jeddah 21589, Saudi Arabia

**Keywords:** artificial intelligence, deep learning, reference standard, diagnostic accuracy, apical periodontitis, cone-beam computed tomography, periapical radiography, incorporation bias, endodontics

## Abstract

The accuracy reported for detecting apical periodontitis using artificial intelligence (AI) is generally interpreted as accuracy against the disease; however, this is usually not the case. Compared with histopathology, single-view periapical radiography detects 16 to 27% of confirmed lesions and 38% with parallax, with a pooled digital sensitivity of 61.0% and a negative predictive value of 41.6%. Two blinded specialists reading 1717 radiographs disagreed on 22% of teeth. Detection depended on lesion size and site: simulated cancellous-bone lesions were invisible below 1 mm in incisors and 3 mm in molars. Of the 44 primary studies identified, 34 (77%) used a label that was a reading of the index image or was unascertainable; eight (18%) used an independent reference—six (14%) suited to contemporaneous detection and two to later treatment outcomes. No cone-beam computed tomography (CBCT) input study had a reference independent of its volume. One commercial platform reported 92.3% sensitivity against same-image consensus and 47.9% against blinded CBCT. The reported accuracy is thus in agreement with a noisy rater; recognising this changes what meta-analyses pool. A model that reproduces human labels would have a lower sensitivity against disease than reported; for real-world models, the direction depends on error dependence that the study design cannot identify.

## 1. Introduction

A diagnostic performance estimate is only as interpretable as what it is compared against. When a network is reported to detect apical periodontitis on a periapical radiograph with a sensitivity of 0.92, that number provides information about apical periodontitis only if the labels originated from something other than the radiograph. This is not the case in most studies; often, the label is a human reading of the same image, so the figure indexes agreement between the two readers, with one of them being a machine. This would be pedantic if two-dimensional radiographs faithfully recorded periapical bone; however, radiographs do not record periapical bone with complete accuracy. Compared with histopathology of human cadaver jaws, a single digital periapical view reached a sensitivity of 0.27 and a cone-beam computed tomography (CBCT) value of 0.89 [1]. Pooled across 48 studies with a histopathological or clinical reference, the advantage of CBCT over 2D imaging lies almost entirely in its sensitivity, because 2D imaging mainly misses the disease [2].

The human readings that supply those labels agree only modestly: two blinded specialists who analysed 1717 periapical radiographs disagreed on 22% of teeth [3]. Therefore, the quantity a same-image design reports represents agreement with a noisy rater. Its distance from sensitivity against disease is not identifiable, and its direction depends on how the model’s errors relate to those of the reader; under the scenario examined below, the reported figure is larger. One commercial platform family has been evaluated both against readers of the index image and against blinded CBCT; the audit returns to this pair as an illustration of the distinction. What a title names and what such a design measures are two different quantities, and the gap between them is systematic rather than incidental.

Endodontics is an unusually tractable setting for this problem for three reasons. First, discordance between index modality and disease has been measured against tissue for two decades. Second, the same task on CBCT volumes reaches far higher agreement [4], so the gap has a measured alternative. Third, an independent reference is often obtainable, since CBCT is already indicated for specified endodontic problems [5]. The first of these reasons is what this review builds on: tissue-referenced discordance evidence gives a same-image label a measured sensitivity of its own.

Earlier reviews recorded the variation in reference standards without treating it as measurement error. Reference standards vary across this literature: high heterogeneity preventing pooling across 34 studies [6]; a call for validated reference standards from a QUADAS-2 appraisal of 30 panoramic studies [7]; a lack of histological correlation in CBCT studies [8]; a case where human expert interpretation was used as the surrogate reference in all 47 studies of one review, with the models being “fundamentally optimized to replicate human expert opinion rather than to independently discover absolute pathological truths” [9]; and incorporation bias identified where the reference and index modality were both based on CBCT [10]. In each case, the reference standard is treated as a source of heterogeneity rather than as a measurement whose error propagates into the pooled numbers. The same reviews cite cadaver histopathology [1,11,12], but they apply it in one direction only: it is used to justify CBCT for modality comparison and is not applied to the 2D labels the AI models were trained against.

This review addresses the following four topics: what tissue shows about radiographs; what radiographs miss in terms of lesion size and site; what the labels in the 44 AI studies identified here were compared against; and what a reported figure therefore measures. Each topic starts with a claim and ends with what it establishes, and names one anchor value and states why it is important. The method by which the studies were identified and coded is set out first; the four topics follow. Technical terms are defined in Table 1.

## 2. Approach

### 2.1. Review Design and Adherence

This article comprises a declared narrative review that reports counts and proportions based on a structured search. The choice of review is based on how the audit’s categories were derived. The six reference standard categories that are used to organise the audit were not fixed in advance; they were developed iteratively as the Methods sections of the identified studies were read. Meanwhile, the mixed and unascertainable categories exist because studies were found that fitted neither a same-image nor an independent reference. The classification framework was therefore developed iteratively during this narrative audit rather than fixed in advance, with the sentence supporting each decision recorded verbatim alongside it. A systematic review can also refine or expand coding categories during extraction, provided the changes are documented; the argument presented here concerns how this audit was conducted, rather than a limit on what the systematic form permits. This review’s contribution is conceptual—an account of what a class of estimates measures—and no effect is pooled, because pooling across reference standards that differ in kind is not defensible. What the narrative design does not deliver is a representative estimate for the literature as a whole; the proportions in the section on the third topic describe the 44 studies identified, and are presented as such.

We adopt a standard narrative review design rather than a systematic review design. This review follows SANRA, the six-item scale for assessing the quality of narrative reviews [13]. No PRISMA adherence is claimed: no protocol was registered and screening was not independently duplicated.

### 2.2. Search Strategy

Three searches, each serving a distinct part of the argument, were conducted using PubMed: the first on 3 August 2026, and the second and third between 3 August and 18 September 2026. Table S3 provides the available search terms; recorded search dates; restrictions; deduplication, screening, and coding procedures; along with the PubMed queries as they were logged when run. The queries were developed iteratively and each retrieved a capped number of records, so the table documents the search as it was conducted rather than a protocol from which the same set of records could be regenerated. No language restriction was applied, and most queries carried no publication-date limit. The first search identified the primary AI studies of periapical lesion detection, segmentation, or grading, combining disease terms (periapical lesion, apical periodontitis, periapical radiolucency, periapical, endodontic) with method terms (artificial intelligence, deep learning, convolutional neural network, CNN, U-Net, segmentation, detection) and, in some queries, with imaging terms (periapical radiograph, intraoral radiograph, cone-beam computed tomography) or reference standard terms (reference standard, ground truth). The second search identified evidence of discordance—imaging performance against a reference independent of the index image (histopathology, histolog∗, cadaver, gold standard, reference standard) and observer-agreement studies. The third search identified the methodological literature on reporting and appraisal instruments, imperfect and missing reference standards, verification and incorporation bias, and dependent errors. The reference lists and citing articles of four anchor studies of the discordance evidence and of one methodological paper, named in Table S3, were also traced through the OpenAlex citation index (accessed on 4 August 2026). Eligibility required being a primary study applying an AI system to intraoral periapical, panoramic, or CBCT imaging and reporting performance for apical periodontitis separately from other findings; reviews, editorials, conference abstracts, and studies in which periapical lesions formed one incidental class were excluded. Two studies concerning apical periodontitis did not meet that requirement; they are retained in Table S1, with the reason supporting the exclusion, so that the exclusion is auditable. Forty-six studies were identified and 44 met these criteria (Table S1).

### 2.3. Coding of the Reference Standard

The full text was obtained for all 46 studies, so every study was coded based on its Methods section (Table S1). Each of the 46 identified studies was coded on 47 fields covering bibliography, input modality, reference standard, annotation provenance and quality, validation design, dataset size, labelling-platform overlap, and first-author country. Screening and coding were performed in a single pass with generative AI assistance (Claude Opus 5, Anthropic) directed by the authors, and every reference standard classification was verified by the authors against the verbatim sentences in the Methods section recorded in Table S1 (Table S3).

Three conventions governed the coding: how the reference standard is categorised and counted, the units in which dataset size is expressed, and how annotation quality and the readers’ information are recorded. The reference standard was coded into six mutually exclusive categories: a human reading of the same image the model consumed; CBCT verification of a 2D index image; histopathology; surgical or clinical outcome; a mixed reference combining index image reading with additional information; or not ascertainable. The first and last categories establish no independent reference and are counted together. Dataset sizes were harmonised to imaging units—radiographs for 2D, volumes or examinations for 3D—but never to teeth, roots, lesions, slices, or augmented copies. For annotation quality, an empty cell denotes a field the published Methods section did not report, which is itself a finding, and separate fields record whether readers have information beyond the index image and whether any agreement coefficient pertains to the labels themselves. A data dictionary accompanies Table S1 as Table S2.

### 2.4. Figures and Supplementary Tables

The supporting material is divided between the two figures, which show the plotted evidence, and the supplementary tables, which show the audit and the methods. One figure assembles the reference standard evidence map (Section 2.8) and the other the scenario analysis (Section 2.18), with every plotted value tracing to a named source. The figures were drawn in Python (version 3.13) with Matplotlib (version 3.11). The search and coding methods, search log, and prognostic study sensitivity analysis are shown in Table S3. With the method stated, the first topic follows: what radiographs show when they are judged against tissue.

### 2.5. What Tissue Shows About Radiographs

In this section, we claim that two-dimensional periapical radiographs identify a minority of histologically confirmed apical periodontitis. The anchor value of 0.27 is the sensitivity of a single digital periapical view in human cadavers against histopathology, compared with 0.89 for CBCT in the same material.

This value is based on 67 teeth (86 roots) from unclaimed bodies, read blind by several raters against histopathological sections; sensitivity was 0.27 for a single digital periapical view, 0.38 for digital with parallax, and 0.89 for CBCT [1]. A companion analysis of the same series reported 0.16 for a single film view and 0.37 for film with parallax [14]. The following four properties make this the anchor value: the reference is the tissue rather than another image; human material is used; there was more than one rater and the raters were blinded; and the images were acquired with contemporary digital sensors, the type of acquisition that the AI studies use. The following two points govern how these values are used: the 0.16 film figure sets the lower bound used here, and the two papers analyse one cadaver series, not two.

One animal series corroborates these results. In a dog model with histological verification of 83 roots, sensitivity was 0.77 for periapical radiography against 0.91 for CBCT [11]. These results are relevant because histological induction and verification of apical periodontitis is only possible experimentally. They corroborate the direction of the cadaver findings, not their magnitude, and no human estimate in this review relies on them. The results appear in the evidence map (Section 2.8) as animal evidence only and are not used in the scenario analysis.

A three-dimensional comparator is imperfect against tissue. CBCT reached a sensitivity of 0.89 in the human cadaver series and 0.91 in the dog model [1,11], which are not fixed properties of the modality. A second human cadaver series of 223 teeth (340 roots) demonstrated CBCT sensitivity, specificity, and negative predictive values that were significantly lower in root-filled than in non-root-filled teeth [12]—which matters for AI datasets, in which root-filled teeth are heavily represented [15]—and uncorrected patient motion degraded it further [16]. Syntheses have also failed to settle on a value: two pooled CBCT estimates differ by several points, and neither states its own reference [17,18]. A CBCT reference has a higher reference accuracy than the image it replaces without being a fixed-performance instrument.

Histology-referenced evidence of this kind is scarce. In the only systematic review of this issue, seven articles were eligible, all of them cadaveric studies against a histopathological reference, and no clinical, histology-referenced estimate of two-dimensional sensitivity for detection exists [19]; the one clinical correlation with surgical or histopathological findings covers 60 patients [20]. Other articles have addressed different questions: a clinical series against histology addressed lesion type rather than presence [21], and a meta-analysis of confirmed lesions reported an accuracy—the metric (TP + TN)/N—for a mixture of references [22].

Even when a lesion is observed, the image and tissue correspond only moderately. In cadaver blocks of root-filled teeth, a radiographic lucency carried 9.2-fold higher odds of histological inflammation than an intact apex, yet the two classifications often disagreed [23], and radiographically successful root-filled teeth spanned uninflamed to mildly inflamed histologies [24]. Calibration to tissue does not alter this. The periapical index is anchored to histologically verified reference images [25]: a reading of the image whose calibration is tissue-derived, not a measure of disease. Where defects were created rather than grown, intraoral radiography detected a minority of what CBCT detected in full [26]. However, a defect of known geometry is not apical periodontitis, so such figures describe the ceiling the image imposes rather than sensitivity against disease.

In this section, the following is established: Against tissue, a single digital periapical radiograph shows roughly a quarter of apical periodontitis and CBCT roughly nine-tenths. The evidence is ex vivo, rests on two human cadaver series and one animal series, and shows CBCT to be a better comparator, but not a perfect one.

### 2.6. What Radiographs Miss According to Lesion Size and Site

The difference between the performance of CBCT and a single radiograph lies in the disease that the radiograph does not show; which lesions are not shown is a key question. In this section, we claim that the missed fraction is not random: what the radiograph fails to show is governed by lesion size and by the bone the lesion occupies. The anchor value is the site-specific visibility threshold based on a virtual simulation platform—lesions confined to cancellous bone could not be visualised below 1 mm in the incisor, 2 mm in the premolar, and 3 mm in the molar [27].

The platform anchors this topic because it controlled the lesion: the platform was built from micro-computed tomography of a cadaver mandible, so lesion geometry and location were fixed exactly, and the thresholds are site-specific. That control is also its limit. A defect of known geometry is not an inflammatory lesion, so the thresholds describe what the image can resolve, not the sensitivity against disease.

Thirty years of experimental studies agree on the direction of the effect of lesion size and site on detection. For simulated lesions analysed by 16 observers, detectability rose with cortical plate involvement and was higher in cortical than in trabecular bone [28]. Lesions confined to the spongiosa became detectable only above the millimetre thresholds given above [27]. With simulated lesions of known bur diameter, both CBCT and periapical radiography showed a size threshold, with CBCT’s threshold being lower [29]. These studies measure geometry, not biology.

The classic interpretation of this literature states more than the evidence carries. Bender and Seltzer’s experimental observation of lesions in bone [30] is usually interpreted as showing that a lesion confined to cancellous bone does not appear on a radiograph until the cortex is involved; no numeric threshold is taken from it here, since their millimetre figures are bur depths, not detection thresholds. Chang et al., by contrast, analysing 129 roots against a CBCT reference, found that lesions in cancellous bone were identified nearly as often as lesions in cortical bone [31]. The two measure different things—a minimum detectable size under controlled conditions, and agreement between two readings in a clinical sample—and what is apparent in both is direction, not magnitude: small lesions are missed more often than large ones, by an amount that depends on the material studied.

In living patients, with CBCT as a comparator, the same principle holds at larger scale. Across 888 examinations covering 1508 teeth, sensitivity was 0.55 for periapical and 0.28 for panoramic radiography against CBCT [32]; the value of 0.55 is the one clinical value carried into the scenario analysis. Four further comparisons agree on the direction of this difference: a paired-root series [33], a study of lesion size on the two modalities [34], a prospective panoramic series [35], and one model analysing both modalities against one CBCT reference [36]. All four found that the two-dimensional image revealed a fraction of what CBCT shows. Pooled across eight studies, digital radiography sensitivity was 61.0% (95% CI 38.3–79.8) at a negative predictive value of 41.6% (95% CI 28.0–56.6), against a pooled CBCT sensitivity of 94.6% [17]. A negative two-dimensional reading in that synthesis had less than a 50% chance of being correct. A further synthesis gives the same range [37], and the pooled value of 0.61 falling inside the 0.16 to 0.77 range from single studies represents a consistency check, not an additional measurement.

Size dependence propagates directly into AI performance. Across 358 root-filled teeth from 167 CBCT scans, referenced to two dentists’ consensus on the same volumes, apical lesion sensitivity rose from 33.6% for sub-millimetre lesions to at least 80% at 1 to 5 mm [38], and another CBCT input evaluation found sensitivity rising once the smallest lesions were excluded [39]. A same-volume consensus is not a tissue reference, so these figures locate where a model’s sensitivity sits across the size range, not how much disease is present. Detection varies by site within a single model as well: on panoramic radiographs referenced to a reading of the same images, one system detected roots in the mandible far more often than in the maxilla [40], and another was several times less sensitive in canines than in its best tooth group [41]. A single headline sensitivity thus averages over a severalfold spread according to size, jaw, and tooth type, and because no study stratifies two-dimensional sensitivity by lesion diameter against histopathology, the two cannot be joined within one design. Some of what the reader does not report is nonetheless recorded in pixels—radiomic texture features separated CBCT-positive, radiograph-negative regions from true negatives [42]—and a model trained to reproduce human labels is trained to discard that signal.

In this section, we establish the following: A radiograph’s missed fraction is concentrated in small lesions and in cancellous bone, with the threshold varying by site. In living patients, as the periapical, panoramic, and pooled digital radiography sensitivities against CBCT given above imply, between a third and three-quarters of CBCT-visible disease is absent from the image an AI label is drawn from. Because no study stratifies histology-referenced sensitivity by lesion size, a model’s headline sensitivity depends on a case mix that is rarely reported.

### 2.7. What the Labels in AI Studies Were Compared Against

If a radiograph shows a minority of a disease and misses it selectively, then what a model’s label was compared against decides what a reported figure can mean, and where those labels came from is the next question. In this section, we claim that, in the studies identified, the label a model is trained on and tested against is most often a clinician’s reading of the same image that the model sees. The anchor value is the audit count itself: 34 of the 44 included studies (77.3%) used a reference that was a reading of the index image or was unascertainable.

The audit count is this topic’s anchor because it is a count rather than an estimate: every one of the 44 studies was coded from its Methods section, and the verbatim sentence supporting each classification is shown (Table S1). It is nonetheless a descriptive finding about the studies included here, not a population estimate for the literature, and every proportion below is to be interpreted from that perspective.

### 2.8. What the Published Labels Are

**Figure 1:**
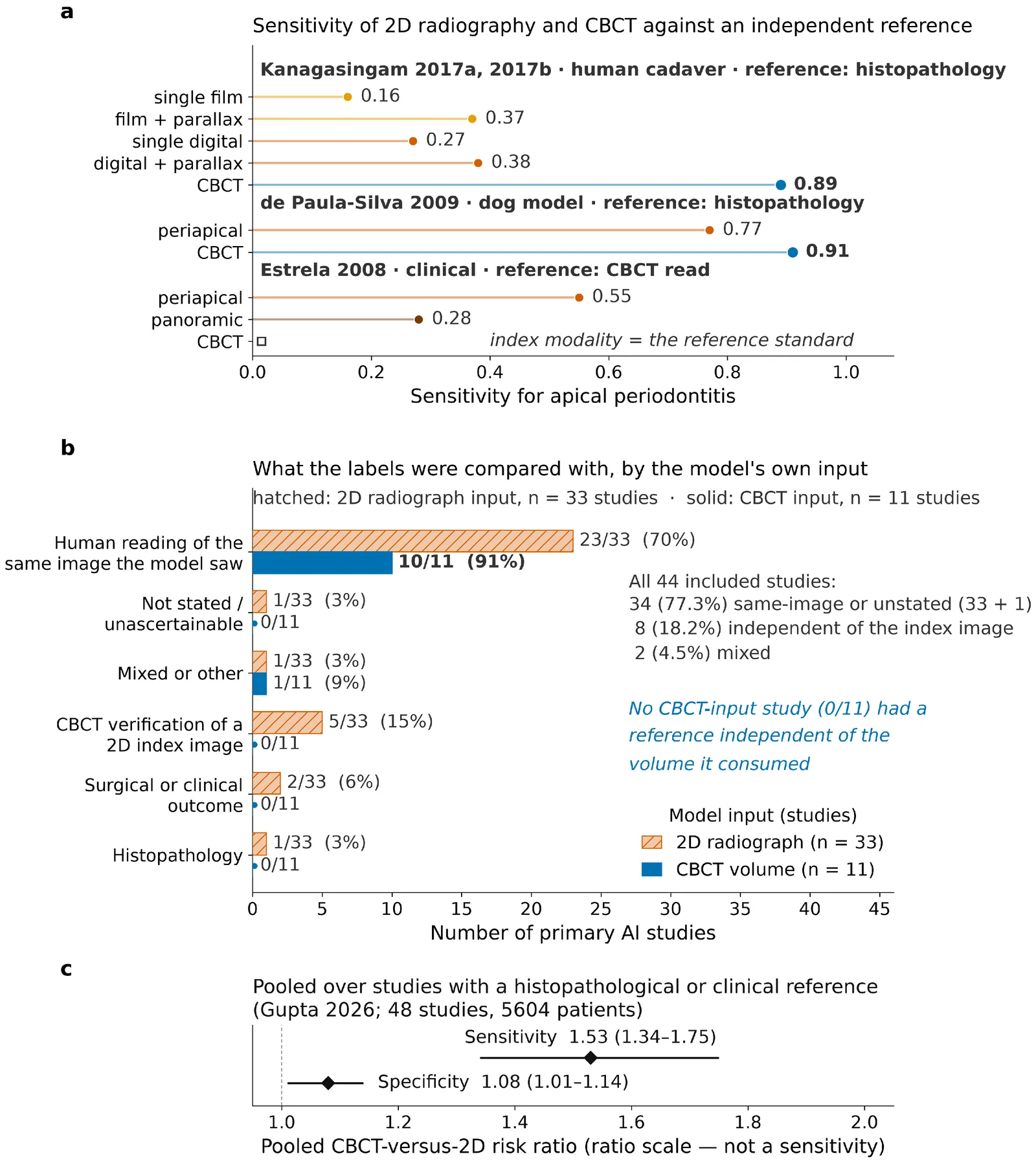
Reference standard evidence map for AI detection of apical periodontitis. (**a**) Sensitivity of each imaging acquisition technique against an independent reference, by study. Kanagasingam 2017a [1] and 2017b [14], human cadaver mandibles (67 teeth, 86 roots; mean 5 raters), reference histopathology: single film 0.16, film plus parallax 0.37, single digital 0.27, digital plus parallax 0.38, CBCT 0.89. de Paula-Silva 2009 [11], dog model (83 roots), reference histopathology: periapical 0.77, CBCT 0.91. Estrela 2008 [32], clinical (888 examinations, 1508 teeth), reference a CBCT reading: periapical 0.55, panoramic 0.28; the open square marks CBCT, for which sensitivity is not estimable because the index modality is itself the reference standard. (**b**) Distribution of the reference standard actually used in the 44 included primary AI studies, split by the image the model consumed. Percentages are calculated within each input modality subgroup—out of 33 for 2D radiographs and out of 11 for CBCT volumes—not out of 44; the two subgroup totals appear in the key. Across all 44 studies, 34 (77.3%) used a reference that was a reading of the index image or was unstated (33 a human reading of the same image, 1 unascertainable), 8 (18.2%) a reference independent of the index image (6 appropriate for contemporaneous detection, 2 a later treatment outcome), and 2 (4.5%) a mixed reference that was neither a reading of the index image alone nor independent of it. No CBCT input study (0 of 11) had a reference independent of the volume the model consumed: ten used a reading of the same volume, and the eleventh used a composite that incorporated it. All 44 studies were coded from their Methods sections; two further studies were identified and excluded as out of scope. (**c**) Pooled CBCT-versus-2D risk ratios from Gupta 2026 [2] (48 studies, 5604 patients), plotted on a ratio scale that is not a sensitivity, with a reference line at 1.0. These pooled estimates—sensitivity 1.53 (95% CI 1.34–1.75) and specificity 1.08 (95% CI 1.01–1.14)—span six pathology categories combined (periapical lesions, root fractures, endodontic complications, root resorption, third molar issues, and root perforations) and are therefore not specific to periapical lesions. The 2D radiograph series in panel (**b**) is drawn with a light fill with diagonal hatching so the paired bars remain separable in greyscale and in monochrome print.

Of the 44 studies, 33 (75.0%) state an explicit same-image reference and 1 (2.3%) has a reference that cannot be determined even from its methods. The largest group provides explicit statements rather than no information: the reference standard in each of the 33 studies is described as an expert reading or manual annotation of the images the model received. What follows is a limitation on interpretation, not a defect in execution: a figure obtained this way describes agreement with a reader. A reference independent of the index image is present in eight studies (18.2%)—six appropriate for contemporaneous detection and two a later treatment outcome, separated below—and a mixed reference, combining an index image reading with additional information, in two studies (4.5%) (Figure 1b).

Two of the eight independent reference studies (Bennasar et al. and Lee et al., both Table S1) label preoperative radiographs by treatment outcome at three and nine years, rather than using a contemporaneous reference for the lesion in the image. Their labels are independent of the index image, which is the property the audit codes, but they validate prognosis rather than detection. Separating them, six of the 44 studies (13.6%) use an independent reference appropriate for contemporaneous detection—five use CBCT verification of a 2D index image and one uses histopathology—and two (4.5%) use an independent outcome used for prognosis. Removing the two studies from the denominator changes the headline proportions in one direction only: the same-image-or-unascertainable share rises from 34 of 44 (77.3%) to 34 of 42 (81.0%), the independent share falls from 8 of 44 (18.2%) to 6 of 42 (14.3%), and the mixed share is 2 of 42 (4.8%). The two studies are retained in the principal denominator because they are primary AI studies of apical periodontitis on radiographs that meet the stated eligibility criteria, and because retaining them credits the literature with more independence than a detection-only count would; the figure of 8 in 44 is the more generous of the two readings (Table S3). Counts for CBCT input are unaffected (0 of 11 independent), since both studies use intraoral periapical input.

### 2.9. Neither Input Modality Escapes the Arrangement

Of the 44 studies, 33 take a 2D radiograph as input and 11 a CBCT volume, and the dimensionality of the input does not determine where the label comes from. Independence is least common where it is most feasible. Among the 33 with 2D input, where an independent three-dimensional reference is in principle obtainable, 23 rest on a human reading of the same image, 5 on CBCT verification, 2 on a clinical outcome, 1 on histopathology, and 1 on a mixed reference, with 1 unascertainable. The detection-appropriate independent designs among them are CBCT read blind by two endodontists [43]; radiology reports cross-verified through CBCT review [44], with a companion CBCT-confirmed test set [45]; a composite of CBCT, clinical findings, and specialist consensus (Gosavi et al., Table S1); matched CBCT for radiograph-negative regions of interest [42]; and one histopathology-referenced study [46]. The two outcome-labelled studies (Bennasar et al. and Lee et al., Table S1) are prognostic—independent, without involving detection validation.

For CBCT input, the position is uniform. No CBCT input study (0 of 11) has a reference independent of the volume the model consumed: in 10 studies, the labels came from expert reading or manual segmentation of that volume, and in the remaining study, the reference combines pathology reports for a minority of cases with CBCT reports for the rest. A 3D input does not confer independence, which is a property of where the label came from. The signature such a reference produces—high sensitivity at moderate specificity—is visible in one platform evaluation on molar CBCT scans referenced to two endodontists’ reading of the same volumes [47], and incorporation of this kind is already described in the CBCT literature [10].

### 2.10. One Platform Family Against Two Reference Standards

How far a reference standard can govern a reported number is illustrated, rather than proven, by one commercial AI platform family measured against two reference standards. In a retrospective evaluation of 20 periapical radiographs covering 60 teeth, referenced to one expert and one trainee reading the index images, the platform returned a sensitivity of 92.30% [48]. In a study of 334 preoperative periapical radiographs covering 339 teeth and 796 roots, drawn from prospective outcome cohorts and referenced to CBCT read blind by two endodontists, the same platform family reached a tooth-level sensitivity of 47.9%, against 65.3% for clinicians [43]. The two evaluations differ in more than their reference standard—in sample size, design, population, and evaluation conditions—so the difference cannot be attributed to the reference standard alone. The contrast is consistent with the effect of reference standard choice and is offered as an illustration of it: one figure shows agreement with two readers of the index image, the other shows sensitivity against an independent reference, and only the second is a diagnostic performance estimate against disease.

A headline figure can also deviate from the diagnostic value within a single evaluation: general-purpose large language models assessed against specialist consensus have returned sensitivities approaching 0.9 alongside very low specificities and chance-level agreement [49,50]. A sensitivity reported without its complement describes a model that flags almost everything.

### 2.11. What Label Reliability Is, and Where It Has Been Measured

Where the reference standard is a human reading, its reliability bounds everything downstream, and agreement on radiographic periapical diagnosis has sat at modest levels for half a century [51,52]. The reference value for label reliability here is the largest blinded specialist comparison, as cited in the Introduction: an oral and maxillofacial radiologist and an endodontist reading 1717 pretreatment periapical images in a national practice-based research network reached Cohen kappa 0.56 (95% CI 0.52–0.60), disagreeing on 377 teeth (22%), a disagreement attributed to bone changes masked by overlying anatomy [3]. Under ideal conditions, six observers reading 150 molar periapical radiographs twice reached Fleiss kappa 0.34 and 0.35 [52]; other designs give the same range [53,54] and calibration raises agreement without closing the gap [55]. On CBCT, by contrast, the same task reaches Fleiss kappa 0.88 [4], although agreement on CBCT still varies with reader experience [56]. High agreement does not validate the label. Where agreement between the two modalities is high, as in one medically compromised cohort [57], the claim at issue is untouched: two readers can agree closely on an image that shows a minority of the disease. The image, not the observer, is what is unreliable, and the label inherits that.

### 2.12. Label Reliability in Audited Studies Is Mostly Unreported

In particular, what the preceding measurements establish for the task in general is not available for the audited studies; how good their labels are is, for most, an open question rather than a low number. The number of annotators is unstated in seven studies, and where stated, the median is 2. A quantitative agreement statistic for the study’s own reference labels appears in 17 of the 44 studies; 17 carry no annotator-agreement information at all, 7 describe consensus resolution without a coefficient, and 3 report a coefficient for something other than their own label generation. For 27 of the 44 studies, therefore, neither a label sensitivity nor an agreement coefficient exists from which the label’s quality could be judged, and where a value exists, it can be as low as Cohen kappa 0.27 (Table S1).

### 2.13. Validation, Dataset Size, and Case Mix

The datasets behind the reported figures are seldom externally evaluated, small, and rarely characterised for case mix. External evaluation on data from outside the developing institution is identifiable in 5 of 44 studies; 35 rest on an internal split or cross-validation alone and 4 report no held-out evaluation. The datasets are small: across the 43 studies reporting size, harmonised to imaging units, the median is 312 (IQR 142–1276). Lesion size is stratified in 7 of 44 studies and anatomical region in 7, so a sensitivity measured on an unreported lesion size distribution carries no guarantee of transport to a screening population, where small lesions predominate. Across the wider literature, a synthesis of 12 panoramic deep learning studies reports sensitivity from 26% to 100% and specificity from 51% to 100% [58], a range too wide to describe one quantity and consistent with comparators that differ in kind.

### 2.14. Where the Labelled Data Come from

The reference standard problem has a geographic aspect: the studies have first authors in a small number of countries, and a CBCT requirement would narrow them further. Of the 44 studies, 39 have an identifiable first-author affiliation country, spread across 19 countries; the four most frequent countries—Turkey, China, Saudi Arabia, and India—account for 19 of the 39 studies, and none has a first author in sub-Saharan Africa or a low-income country. Dental imaging datasets and clinical AI databases generally show the same concentration [59,60,61], while disease burden sits elsewhere [15,62]. Those populations are also least likely to have CBCT available, so a blanket CBCT requirement would confine validated evaluation to the settings that need it least—a generalisability failure as much as an equity one, since a model validated only where CBCT is available has been validated only in that case mix [63].

In this section, we establish the following: In the 44 studies identified, roughly four in five report performance against a reading of the same image or an unstated reference, six against an independent reference suited to detection, and two against a later outcome; no CBCT input study has an independent reference; label reliability is unmeasured in most studies and modest where measured; and the datasets are small, internally validated, poorly characterised for lesion size, and concentrated in a few countries. Each of these points describe the identified sample, not the literature.

### 2.15. What a Reported Figure Therefore Measures

The audit found the same image arrangement in most of the identified studies, and the first two topics described what such a label misses; together, they determine what a figure reported against that label measures. As stated in the Introduction and supported by the three other topics, in this section, we claim the following: when a label is a reading of an index image, the reported sensitivity is an agreement statistic between two readers of one radiograph, with one of them being a model. The anchor value is the scenario value calculated in Section 2.18: a model reporting a sensitivity of 0.90 against a same-image label whose own sensitivity against histopathology is 0.27 would, if it behaved as its label does, have a sensitivity against disease near 0.26.

The scenario figure is the anchor here because it joins the two preceding topics—the label’s measured sensitivity against tissue and the audited prevalence of same-image labels—in one stated scenario whose assumptions can be inspected. What it yields is a scenario value, not a corrected estimate: what a reported figure would mean if the model reproduced its human labels, not what any model achieves.

### 2.16. Naming the Estimand

When a model is trained and tested on expert readings of periapical radiographs, the sensitivity reported is the probability that the model agrees with the reader, not the probability that it identifies the disease; it is properly called concordance with expert 2D reading, which coincides with diagnostic performance against disease only if the reader observed all instances of the disease. Let D denote apical periodontitis as an independent reference would ascertain it, H the human reading of the index image, and M the model’s output. Reported is P(M = 1|H = 1); described in the studies’ titles and abstracts is P(M = 1|D = 1). These coincide only if H = D, and the evidence puts P(H = 1|D = 1) between 0.16 and 0.38 against histopathology in human cadaver material and at 0.55 against CBCT in a clinical series, with two readers of the same image agreeing at kappa 0.22 to 0.76 across the reader studies retrieved here. CLAIM 2024 made the terminological half of this point, retiring “ground truth” for “reference standard” because an assigned label may itself be uncertain [64]; what follows completes it quantitatively.

### 2.17. Three Properties of a Reference Standard and Two Mechanisms of Error

Three properties of a reference standard are easily conflated, and a same-image design fails one of them but not necessarily the others. Reference independence specifies whether the reference was determined without access to the index data. Reference accuracy specifies how well the reference itself approximates the clinical or biological target: a blinded CBCT read is independent of a periapical radiograph, yet CBCT is itself 0.89 to 0.91 sensitive against tissue. Construct validity specifies whether the labelled target is the clinical entity the model is claimed to detect. Which property a same-image reference fails depends on the estimand. A same-image reference is inadequate when the intended estimand is disease status independent of the index image, although it may be appropriate when the target construct is explicitly an image-defined finding: a model whose stated target is radiographic periapical radiolucency, evaluated against expert annotation of that radiograph and reported as concordance, has an adequate reference for its claim, and the problem arises when that agreement is described as apical periodontitis detection. The audit codes independence only; the other two properties are addressed below.

The mechanism by which a same-image reference misleads also needs to be defined, because the following three related phenomena are often treated as one: incorporation bias, reference imperfection, and correlated errors on a shared source. The first, classical incorporation bias, in the taxonomy of Whiting et al. [65], arises when the result of the index test itself forms part of the reference standard. The second is an imperfect reference standard that misclassifies the target condition regardless of its relation to the index test, and the direction in which it distorts an estimate depends on whether its errors are correlated with those of the index test [66]. The third comprises correlated errors on a shared information source that arise when an index test and its reference draw on the same data, so that both fail on the same cases even though neither result informed the other—a source of bias distinct from reference imperfection itself [67]. In the audited studies, the dominant mechanism is the third: Table S1 records no methods sentence in which the model’s own output entered the human label, so classical incorporation bias is not what the audit documents; the model and reader both read one image, and a lesion that an image does not show is missed by both. The reference is also imperfect in the second sense, since the reader’s sensitivity against tissue is what the first topic measured. The distinction matters for the solution: blinding the reader to model output addresses incorporation bias but not correlated errors on a shared image, which only a reference drawn from information the model did not consume addresses.

### 2.18. Ceiling Propagation

**Figure 2:**
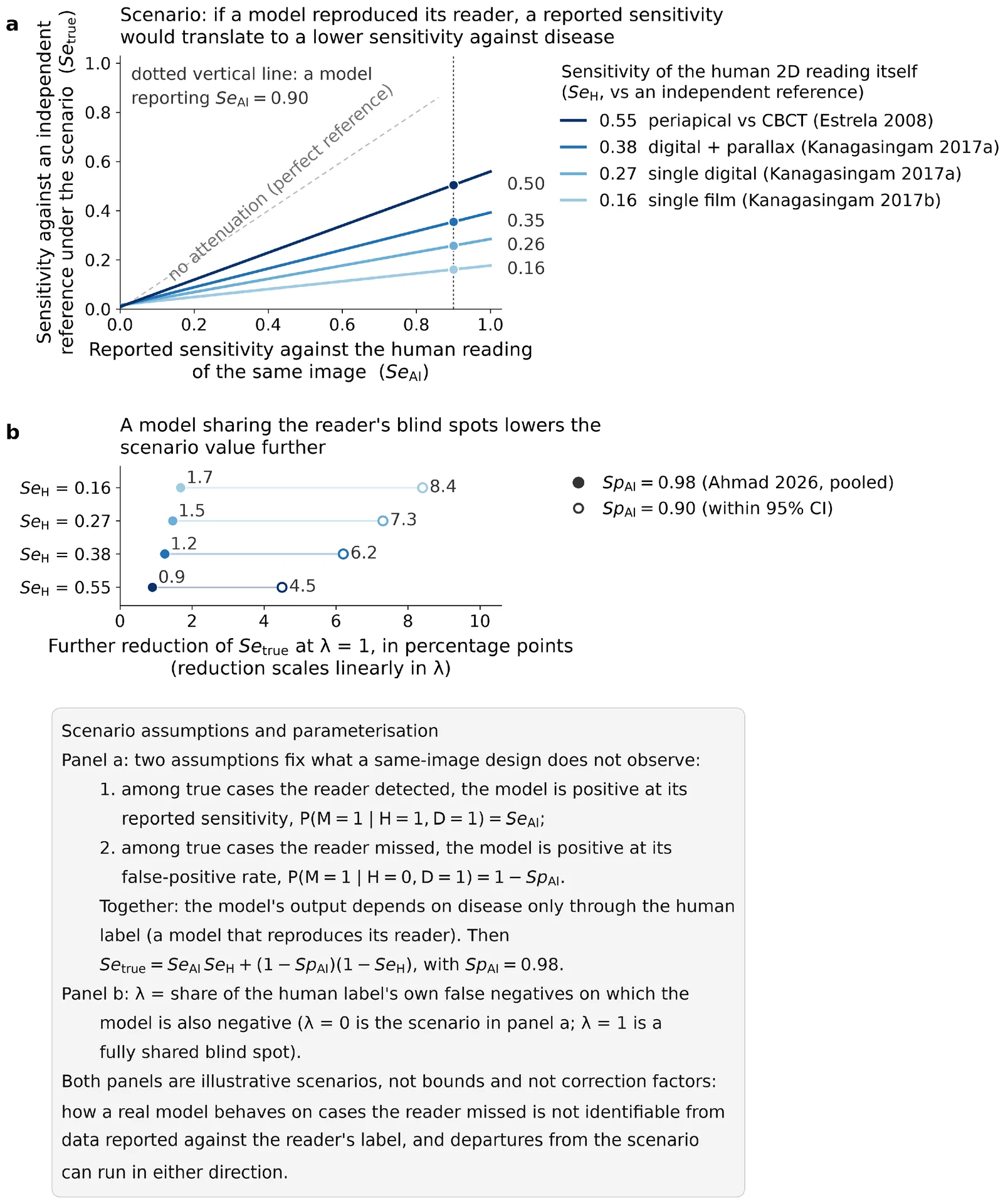
Scenario analysis: what sensitivity reported against a same-image label would mean about disease if the model reproduced the label of a human reader. (**a**) Sensitivity against an independent reference (*Se*_true_) as a function of the sensitivity a model reports against a human reading of the same image (*Se*_AI_), under the following two assumptions: among the true cases the reader detected, the model is positive at its reported sensitivity, P(M = 1|H = 1, D = 1) = *Se*_AI_; among the true cases the reader missed, it is positive at its false-positive rate, P(M = 1|H = 0, D = 1) = 1 − *Sp*_AI_. Together, these state that the model’s output depends on the disease only through the human label. Then, *Se*_true_ = *Se*_AI_·*Se*_H_ + (1 − *Sp*_AI_) (1 − *Se*_H_), with *Sp*_AI_ = 0.98 (Ahmad 2026 [7], pooled specificity 0.98, 95% CI 0.87–1.00). Each curve fixes *Se*_H_, the sensitivity of the human 2D reading itself against an independent reference: 0.16 (single film) and 0.27 and 0.38 (single digital, digital with parallax) from a human cadaver series against histopathology (Kanagasingam 2017a [1] and 2017b [14]); 0.55 from a clinical series against CBCT (Estrela 2008 [32]). The dotted vertical line marks a model reporting *Se*_AI_ = 0.90; the markers on that line read 0.16, 0.26, 0.35, and 0.50 (values to two decimal places throughout this figure and its caption). (**b**) Further reduction in *Se*_true_ at λ = 1, in percentage points, where λ is the share of the human label’s own false negatives on which the model is also negative; the reduction scales linearly in λ. Filled markers use *Sp*_AI_ = 0.98, open markers *Sp*_AI_ = 0.90, a value toward the lower end of that pooled interval. Panel (**b**) shows one departure from the scenario, a model sharing the reader’s blind spots; the opposite departure, a model detecting part of what the reader missed, raises *Se*_true_ and is not plotted because its size cannot be inferred from a same-image study. The caveat on the *Se*_H_ values stated above applies to both panels. Both panels are illustrative scenarios, not correction factors and bounds. The λ parameterisation in panel (**b**) is a single-parameter description of dependent false-negative errors in the spirit of Walter et al. [70], whose own setting (a test measured against an imperfect reference) and estimand differ from those here.

In Figure 2, we ask one question: if a model reproduced the labels of a human reader, what would its reported sensitivity imply about disease? With D, H, and M defined as above, and assuming that the model’s behaviour among disease-positive cases follows its behaviour against the label, sensitivity against disease is given by Equation (1):

*Se*_true_ = *Se*_AI_·*Se*_H_ + (1 − *Sp*_AI_)(1 − *Se*_H_) (1)

Here, *Se*_AI_ and *Sp*_AI_ are the sensitivity and specificity reported against the label, and *Se*_H_ is the sensitivity of the human two-dimensional reading against an independent reference. The exact decomposition, the two assumptions that fix the terms that a same-image design does not observe, the sourcing of each input, and what departures from the assumptions would result in are set out in Supplementary Note S1.

This is a scenario, not a bound and not a property of any particular model. The first term counts true cases the reader detected and the model agreed on; the second counts true cases the reader missed and the model flagged anyway at its false-positive rate. A model that finds part of what the reader missed has a higher sensitivity against disease than the scenario returns; one that shares the reader’s blind spots has a lower one. Neither can be estimated from the data reported against the reader’s label, so the scenario is not a correction and the value in Figure 2 is not a corrected performance estimate. The inputs are taken from the AI literature itself: *Sp*_AI_ = 0.98 [7], whose confidence interval runs from 0.87 to 1.00; *Se*_AI_ = 0.90 from the middle of the range the audited same-image studies report; and four *Se*_H_ values (0.16, 0.27, 0.38, and 0.55) from series using a reference other than the index radiograph [1,14,32]. Those four values are an illustrative range of plausible label sensitivities, not a graded scale; they differ in reference standard and imaged population, and share only the property the calculation requires. A second pooled synthesis cannot serve as an independent check on the reported side: across 24 studies, it gives a sensitivity of 0.94 (95% CI 0.90–0.96) at a specificity of 0.96 (95% CI 0.91–0.98) [68], but does not state its reference standard, which places its inputs inside the arrangement examined here. What a same-image sensitivity value can conceal is visible in one evaluation against two clinicians’ consensus on 120 periapical radiographs, where three general-purpose multimodal models returned sensitivity 95.00% at specificity 13.33%, 20.00% at 98.33%, and 95.00% at 48.33% [69].

Where specificity against the human label is high, the second term is small and almost all of the difference sits in the first, *Se*_AI_ *Se*_H_: within the scenario, the model is credited only with detecting what the label recorded. Here we have presented the whole calculation; what is new here is merging the information, not the inputs themselves, which have been in print since 2008.

### 2.19. Correlated Errors, and Why Their Direction Is Not Identified

The scenario’s second assumption—that the model is positive on the reader’s missed cases at its false-positive rate—is an assumption that a same-image training design is most likely to violate. The violation can run either way. A model trained on labels from readers like the reference reader, having learned their features, may be negative on the small, non-cortical, anatomically superimposed lesions they missed more often than it is on reader-negatives generally. A model trained on independently verified labels, or one exploiting image features the reader does not use, may be positive on those same lesions more often.

The first departure—a model sharing the reader’s blind spots—is examined in panel b through a single dependence parameter λ ∈ [0, 1]: the share of the human label’s own false negatives on which the model is also negative. It enters Equation (2):

*Se*_true_ = *Se*_AI_·*Se*_H_ + (1 − λ)(1 − *Sp*_AI_)(1 − *Se*_H_) (2)

With λ = 0, Equation (2) reduces to the calculation plotted in panel a, and λ = 1 describes a fully shared blind spot; the extra reduction is largest when *Se*_H_ is smallest, reaching 1.7 percentage points at *Se*_H_ = 0.16 and 8.4 points at specificity 0.90 (Figure 2b; algebra in Supplementary Note S1). The opposite departure, a model detecting part of what the reader missed, is not plotted, because nothing in the literature on same-image studies allows its size to be guessed; it is the reason the scenario is not a bound.

The direction of such a gap has been determined formally for a neighbouring problem. Walter et al. [70] consider a test evaluated against an imperfect reference standard and show that apparent sensitivity can be biased in either direction, with the sign depending on prevalence, the operating characteristics of the test and reference, and the covariance of their errors; when the errors are independent, apparent sensitivity always falls below the true value. They are cited for that result—direction is a property of the dependence structure rather than of imperfection as such—and not as a derivation of Equation (1), whose estimand differs. Their baseline and this scenario point opposite ways: under independent errors, the reported figure understates sensitivity against disease, while under imitation it overstates it. Which case describes a given model depends on a dependence that cannot be identified from data reported against the reader’s label, since λ cannot be estimated without observing cases the reference reader missed.

The remedy therefore lies in design rather than in post hoc adjustment, which is why methods for imperfect and missing reference standards [71] belong in a primary study’s methods.

### 2.20. A Second Distinct Failure: Construct Validity

The failure examined so far is one of degree: the label undercounts the disease. The next is one of kind. The propagation above concerns how much of the target a model detects; a separate failure exists in terms of what it detects. A model trained on “radiolucency present” labels detects radiolucency, a superset of apical periodontitis. Across 1611 periapical lesions over 20 years, 90 (5.6%) were non-endodontic [72]; in a 236-report surgical-biopsy series, the fraction was 100 (42.4%), comprising odontogenic cysts, tumours, non-odontogenic cysts, and fibro-osseous lesions [73]. One frame is diagnostic and the other is selected by indication for surgery, so a figure from the second does not transport to the first. Consequently, in the surgical series, about a quarter of the teeth linked to odontogenic cysts were vital, so root canal therapy was unwarranted [73]. Imaging and clinical impression resolve histotype poorly—agreement between clinical and histological diagnoses of granuloma and cyst was near chance across 317 biopsy specimens [74], provisional diagnoses were correct for under half of granulomas and about a third of radicular cysts [75], and a radiopaque lamina did not reliably mark a cyst [76]. Histotype is not the target of any of the 44 studies; it is a distinct clinical question with its own reference requirement, and no imaging reference at any voxel size settles it.

### 2.21. Reporting Completeness and Reference Validity Are Orthogonal

Whether a study’s reference has the three properties set out above is a different question from whether the study describes its reference standard, and the guidelines that govern this literature require only the second. CLAIM 2024, STARD 2015, STARD-AI, PRIDASE 2024, and TRIPOD+AI each ask for the reference method, its rationale, the annotators’ number and qualifications, and their variability, and none asks whether the label was ascertained independently of the index test [63,64,77,78,79]; SANRA has no reference standard provision [13]. A study can satisfy every item with a reference that is a reading of the index image, and PRIDASE admits practical availability as a rationale, which is precisely what same-image consensus offers. QUADAS-2 comes closest and stops short of a criterion: it asks whether the reference standard correctly classifies the target condition, assuming it is 100% sensitive [80], an assumption a label with a sensitivity of 0.27 against histopathology does not meet; QUADAS-AI names inappropriate reference standards [81], with its item set remaining unpublished [82]. Completeness of reporting and validity of the reference standard are therefore orthogonal, and instruments govern only the first—which is itself only partially achieved [83]. The consequences reach past the literature: the higher figure is the one that enters cost-effectiveness models [84], as though it were a sensitivity against disease.

### 2.22. Routes to an Interpretable Figure Without a Cohort-Wide Scan

Four routes to an independent label avoid a cohort-wide scan: partial verification of a subsample with explicit statistical correction, or a latent-class or imperfect-reference model [71]; a sensitivity analysis propagating dependent errors between an imperfect reference and the index test [70]; retaining an ordinal or probabilistic label rather than forcing a dichotomy; and confining the claim to concordance with expert two-dimensional reading, named as such. All studies contain all necessary scanners, no additional routes require a scanner, and each route is a valid design rather than a compromise. The 2D-only workflow is the reality for most patients with apical periodontitis worldwide; a 2D model can be validated, but not against its own input. Additionally, pooling data across sites does not substitute for any of the four routes. Federated learning [85] handles the data concentration half of the problem and not the label half. Ten institutions labelling using the same-image consensus produce a larger same-image-labelled dataset, not a valid one—although a consortium in which a minority of sites contribute independently verified labels would. No such consortium design appears among the 44 studies.

In this section, we have established the following: The sensitivity reported against a same-image label is a measure of concordance with expert reading, and the design that produced it cannot show in which direction it departs from the sensitivity against disease. Construct validity is a separate failure no imaging reference resolves, the reporting guidelines do not ask the one question that fixes what a figure means, and interpretable figures are obtainable without a cohort-wide scan.

### 2.23. Limitations

The routes described above can be explored in primary studies. This review has limits of its own, which affect how applicable its counts and scenario are. The 44 studies are a deliberately assembled sample, not a PRISMA-screened one. Screening was not duplicated, no protocol was registered, and no risk-of-bias instrument was applied, so the proportions are descriptive statistics of this set rather than unbiased estimates of the literature. Because screening and the 47-field coding were not duplicated by a second human coder, the classifications can be inspected against the methods sentences recorded in Table S1 but their inter-coder reliability cannot be reported (Table S3). Reading every Methods section moved the headline proportion by about one percentage point downward, while changing its basis from abstract silence to stated labels (Table S1). One reference standard remains undocumented even with the full text: a study using a public panoramic dataset states that the labelling process and annotator qualifications were not disclosed.

The studies are not fully independent samples in terms of their methods: five share a labelling platform, overlapping authorship, or both, so their reference standards are correlated by construction and the effective number of independent observations behind the headline figure is smaller than 44.

The histopathological evidence anchoring the first topic is thin; it rests on two human cadaver series [1,12,14] and one animal series [11]. The only systematic review of the question found seven cadaveric studies and no clinical studies [19], so the 0.16 to 0.38 range against histopathology is ex vivo and its transferability to living patients is unquantified. Exactly one of the 44 AI studies uses a tissue reference, and a single study is a fragile anchor.

The alternative reference is itself imperfect. Its sensitivity against histopathology, 0.89 and 0.91 [1,11], is high but incomplete and degrades in root-filled teeth [12] and with uncorrected motion [16]. Reader reliability on CBCT varies with training [56], and the two pooled CBCT estimates differ and neither states its own reference [17,18]. Combined 2D imaging can be used to approximate it for population-level estimates [86]. A CBCT reference improves design without yielding a perfect label, and every CBCT-referenced figure here, including the 0.55 carried into Figure 2, carries that imperfection.

The propagation calculation is a scenario analysis rather than a correction. It rests on the assumption that the model’s output depends on the disease only through the human label, which describes a model that reproduces the label of a human reader and is not known to describe any published model. Departures from it are possible in either direction and are not identifiable from data reported only against an index image label, so no numerical correction to any published estimate follows. Walter et al. [70] address the different problem of a test measured against an imperfect reference and show that the sign of the bias there depends on prevalence, the true operating characteristics, and the covariance of the two error sets; their result is cited for that dependence of direction on structure, not as a derivation of Figure 2.

Neither the magnitude nor, for any particular model, the direction of the gap between a same-image figure and sensitivity against disease is identifiable from that figure. Whether and by how much any published estimate departs from sensitivity against disease requires an independent reference whose absence is the subject here. Under the stated scenario, the gap runs downward for sensitivity in case-finding, in confirmation of a suspected lesion, and in severity grading; the scenario is formulated for a dichotomous detection label and makes no statement about change over time. Reading a correction factor off of Figure 2 would claim more than the evidence supports.

The two systematic reviews of overlapping scope cited in the Introduction [6,9] point in the same direction as this audit, which provides reassurance about direction rather than confirmation of magnitude.

### 2.24. Recommendations

Four recommendations follow from the preceding sections, none of which require a new checklist or scanner.

*For researchers reporting a primary study.* State the clinical question the figure answers and whether the reference was ascertained from information the model did not consume. When this is not the case, name the quantity concordance with the expert reading rather than detection; doing so costs an author nothing and changes what a downstream review can pool. When an independent reference is obtainable, a verified subsample with statistical correction recovers an interpretable figure; when it is not, a sensitivity analysis propagating plausible reference standard error replaces the assumption that the label is true.

*For reviewers and authors of syntheses.* Do not pool sensitivity across studies in which comparators range from one annotator’s reading of the index image to a blinded CBCT read by two endodontists; those figures do not estimate a single quantity. Treat reference standard as a stratifying variable rather than as heterogeneity to absorb.

*For editors and the custodians of reporting instruments.* One item is absent from every guideline governing this literature: Further requests that the reference standard be described, which CLAIM, STARD-AI, PRIDASE, and TRIPOD+AI already make, are not needed; instead, a statement of a label’s independence from the index test is needed. An extension to an existing item, with CLAIM item 15 being the natural host, has a better prospect of adoption than a new framework.

*For the field’s research agenda.* None of the 44 studies identified here evaluates either of two clinical questions against an adequate reference: change in periapical status over time, and the distinction between apical periodontitis and non-endodontic radiolucency, which decides 5.6% to 42.4% of periapical lesions and supports opposite clinical actions.

## 3. Conclusions

Two-dimensional periapical radiographs do not record periapical bone reliably enough to define the disease that they are used to diagnose. In human cadaver material, the technique detects between 16% and 38% of histologically confirmed apical periodontitis, and two specialist readers of the same films were found to disagree on more than one tooth in five. Any label drawn from a reading of such an image inherits that uncertainty, so a performance figure measured against one indicates agreement with a human reading rather than detection of disease; roughly four out of five AI studies identified in this article fit this characteristic. Those proportions describe the 44 included studies and are offered as a description of them, not as prevalence estimates for the wider literature; a different search would return a different set of studies. Under the scenario of a model that reproduces the labels of a human reader, sensitivity against disease falls below the reported figure, with an even greater decrease when the label is weakest: a reported value of 0.90 corresponds to approximately 0.26 against an independent reference when the label’s own sensitivity is 0.27. Whether a given model behaves that way depends on how its errors relate to those of the reader, which the design that produced the estimate cannot show. The comparator that would settle the question is itself imperfect, with CBCT being 0.89 to 0.91 sensitive against tissue. The histopathological evidence behind this review comprises two human cadaver series and one animal series.

The broader point, on which all four recommendations rest, is the one CLAIM’s authors reached terminologically when retiring “ground truth” for “reference standard”, given that an assigned label is itself uncertain [64]. Its quantitative completion can be described as follows: an uncertain label separates the quantity reported from the quantity of interest by a distance that the study design cannot identify, so a field that has not measured that uncertainty cannot know what its performance figures mean. In endodontic imaging, uncertainty has been measured repeatedly since 2008; what remains is to use these measurements of uncertainty.

## Figures and Tables

**Table 1:** Terms used in this review.

Term	Definition in This Review
Reference standard	The procedure against which the index test is judged; the best available means of establishing whether the target condition is present. The term is preferred to “gold standard”, which implies a perfect reference, and to “ground truth” (below).
Index test	The test under evaluation; here, the AI system’s output on a radiograph or CBCT volume.
Estimand	The quantity a study sets out to estimate. For a detection claim, it is the probability that the model is positive given that the disease is present; for a same-image design, the quantity actually estimated is the probability that the model agrees with a human reader.
Ground truth	A label treated as true by definition. Its use is avoided here, following CLAIM 2024, because the labels in this literature are readings of images that show only a minority of the disease, so calling them true assumes the answer to the question the review addresses.
Incorporation bias	Bias that arises when the result of the index test itself forms part of the reference standard, so the two are not judged independently.
Imperfect reference standard	A reference that misclassifies the target condition in some cases, whatever its relation to the index test. Histopathology approaches perfection for apical periodontitis; CBCT and radiographic reading do not.
Correlated errors	Errors in the index test and the reference that occur for the same cases more often than chance, typically because both draw on one information source; for example, a lesion an image does not show is missed by model and reader alike.
Partial verification	A design in which only a subsample of cases receives the reference standard; interpretable when the subsample is chosen by a known rule and the estimate is corrected accordingly.
Latent class analysis	A statistical method that estimates the diagnostic performance of several imperfect tests without a perfect reference by treating true disease status as an unobserved class.
Attenuation	Reduction in an estimated quantity below its true value because of measurement error in what it is compared against. Used here only for a stated scenario, since the direction of the gap for a real model is not identified.
Construct validity	Whether the labelled target corresponds to the clinical entity the model claims to detect; a model labelled for radiolucency detects radiolucency, not apical periodontitis specifically.
Reference independence	Whether the reference was determined without access to the index data; a reading of the same image the model consumed is not independent.
Reference accuracy	How well the reference itself approximates the clinical or biological target, whatever its provenance; a blinded CBCT read is independent of a radiograph yet imperfect against tissue.

## Data Availability

All data supporting the conclusions of this review are derived from the published articles cited in the reference list. The complete reference standard audit, including the verbatim quotation supporting every coding decision and a per-study record of the full-text retrieval routes attempted and their outcomes, is provided as Table S1 with its accompanying data dictionary (Table S2). The two figures are reproducible from the formulae, parameter values, and sources stated in the estimand analysis and in the figure captions; no primary imaging data were generated or analysed.

## Supplementary Materials

The following supporting information can be downloaded at https://www.mdpi.com/article/10.3390/diagnostics16193225/s1, Table S1, Reference standard audit of the 46 primary AI studies of periapical lesion detection, segmentation, and grading identified by the search (46 rows, 47 coded fields, comprising the 44 included studies and 2 retained with an explicit exclusion reason, with the verbatim methods sentence supporting every reference standard classification and a per-study record of full-text provenance); Table S2, Field-level data dictionary for Table S1; Table S3, Search and coding methods (the available search terms, recorded search dates, restrictions, deduplication, citation tracing, and screening and coding procedures, with a log of the PubMed queries as run; and the sensitivity analysis excluding the two prognostic studies); Supplementary Note S1, Derivation of the scenario calculation (the exact decomposition, the two assumptions that fix the unobserved terms, the sourcing of each input, the λ parameterisation, and what departures from the assumptions would result in).

## Abbreviations

The following abbreviations are used in this manuscript:

AI	artificial intelligence
AP	apical periodontitis
AUC	area under the receiver operating characteristic curve
CBCT	cone-beam computed tomography
CI	confidence interval
CLAIM	Checklist for Artificial Intelligence in Medical Imaging
CNN	convolutional neural network
DOI	digital object identifier
FOV	field of view
IOPA	intraoral periapical (radiograph)
IQR	interquartile range
NPV	negative predictive value
PAI	periapical index
PPV	positive predictive value
PRIDASE	Preferred Reporting Items for Diagnostic Accuracy Studies in Endodontics
QUADAS	Quality Assessment of Diagnostic Accuracy Studies
ROI	region of interest
SANRA	Scale for the Assessment of Narrative Review Articles
STARD	Standards for Reporting Diagnostic Accuracy Studies
TRIPOD	Transparent Reporting of a multivariable prediction model for Individual Prognosis Or Diagnosis
2D/3D	two-dimensional/three-dimensional
