# Supplementary material for: Reference Standards in Artificial Intelligence Detection of Apical Periodontitis: A Narrative Review of What Reported Accuracy Measures
Source: Diagnostics (Basel). 2026 Oct 6;16(19):3225. doi: 10.3390/diagnostics16193225 (PMC13664220; doi:10.3390/diagnostics16193225)
Supplement: Supplementary file 1 [file diagnostics-16-03225-s001.zip › 02_Table_S1_Reference_standard_audit.pdf]

Table S1. Reference standard audit of the 46 primary AI studies of periapical lesion detection, segmentation, and grading identified by the search.

Forty-six studies were identified; 44 are included in the denominator and 2 are retained with an explicit exclusion reason. All 46 rows are included from the published Methods sections. An empty cell means the published article does not report that field. Linked by row_id across the six sub-tables.

S1a. Study identification

row_id	first_author	year	journal	title	doi	pmid	pmcid	url	aff_country
S01	Adıgüzel	2026	BMC Oral Health	Zero-shot detection of apical periodontitis on periapical radiographs using multimodal large language models: diagnostic accuracy and error patterns	10.1186/s12903-026-09026-7	42351136		https://doi.org/10.1186/s12903-026-09026-7	Turkey
S02	Akyüz	2026	J Endod	Artificial Intelligence-assisted Periapical Radiographic Assessment: Lesion Detection, Endodontic Complication Analysis, and Review of Clinical Treatment Recommendations	10.1016/j.joen.2026.04.008	42019676		https://doi.org/10.1016/j.joen.2026.04.008	
S03	Almutairi	2026	Restor Dent Endod	Diagnostic accuracy of ChatGPT-5 in evaluating root canal treatment and periapical pathosis on periapical radiographs	10.5395/rde.2026.51.e29	42413993		https://doi.org/10.5395/rde.2026.51.e29	Saudi Arabia
S04	Bubna	2026	Braz Dent J	Can Large Language Models Detect Periapical Lesions in Anterior Teeth? A Comparative Study	10.1590/0103-644020256861	41538516		https://doi.org/10.1590/0103-644020256861	Brazil
S05	Erkal	2026	BMC Oral Health	Automated periapical lesion segmentation and area-based PAI indexing: a comparative deep learning study on periapical radiographs	10.1186/s12903-026-08645-4	42144602	PMC13383545	https://doi.org/10.1186/s12903-026-08645-4	Turkey
S06	Hassanein	2026	Bioengineering (Basel)	Artificial Intelligence Versus Human Dental Expertise in Diagnosing Periapical Pathosis on Periapical Radiographs: A Multicenter Study	10.3390/bioengineering13020232	41749771	PMC12937961	https://doi.org/10.3390/bioengineering13020232	Egypt
S07	Kunnath Menon	2026	Dent J (Basel)	Evaluating the Test Characteristics of a Prototype for AI-Assisted Radiographic Detection	10.3390/dj14020096	41744934	PMC12939923	https://doi.org/10.3390/dj14020096	United Arab Emirates
S08	Lee	2026	Int Endod J	Mamba-Based Deep Learning Model for Automated Periapical	10.1111/iej.70170	42108657		https://doi.org/10.1111/iej.70170	Korea

row_id	first_author	year	journal	title	doi	pmid	pmcid	url	aff_country
				Index Classification Using Periapical Radiographs and Clinical Metadata					
S09	Marwaha	2026	Maedica (Bucur)	AI-Based Detection of Periapical Lesions in Endodontic Radiographs: a Diagnostic Accuracy Study	10.26574/maedica.2026.21.1.64	41978871	PMC13061437	https://doi.org/10.26574/maedica.2026.21.1.64	India
S10	Mayer	2026	Int Dent J	Image Resolution's Impact on Artificial Intelligence & Human Accuracy in Full-Mouth Radiographic Analysis	10.1016/j.identj.2026.109574	42030751	PMC13125990	https://doi.org/10.1016/j.identj.2026.109574	Israel
S11	Obuchowicz	2026	J Clin Med	Radiomics as a Decision Support Tool for Detecting Occult Periapical Lesions on Intraoral Radiographs	10.3390/jcm15030971	41682653	PMC12898856	https://doi.org/10.3390/jcm15030971	Poland
S12	Sadr	2026	Oral Radiol	Web-based AI application for enhanced dental disease diagnosis using advanced object detection integrated with transformer-based attention mechanism	10.1007/s11282-025-00886-3	41528683		https://doi.org/10.1007/s11282-025-00886-3	Iran
S13	Scarano	2026	J Imaging	YoLeTooth: A Unified Framework for Joint Tooth Segmentation and Periapical Lesion Detection in Panoramic Radiographs	10.3390/jimaging12060272	42346935	PMC13301822	https://doi.org/10.3390/jimaging12060272	Italy
S14	Shahmaleki	2026	BMC Pediatr	Automated detection of periapical lesions in pediatric panoramic radiographs using YOLOv7: a retrospective internal validation study	10.1186/s12887-026-07354-9	42477623		https://doi.org/10.1186/s12887-026-07354-9	Turkey
S15	van Nistelrooij	2026	Int Dent J	Development and Validation of AI System for Tooth Detection and Diagnosis in Dental Radiographs	10.1016/j.identj.2026.109576	42044605	PMC13134024	https://doi.org/10.1016/j.identj.2026.109576	Netherlands
S16	Öksüzoğlu	2026	BMC Med Imaging	Fully automated 3D segmentation of periapical lesions on CBCT images using nnU-Net v2	10.1186/s12880-026-02316-0	41913134	PMC13159272	https://doi.org/10.1186/s12880-026-02316-0	
S17	Allihaibi	2025	Int Endod J	The detection of apical radiolucencies in periapical radiographs: A comparison between an artificial intelligence platform and expert endodontists with CBCT serving as the diagnostic	10.1111/iej.14250	40317970	PMC12254525	https://doi.org/10.1111/iej.14250	Saudi Arabia

row_id	first_author	year	journal	title	doi	pmid	pmcid	url	aff_country
				benchmark					
S18	Arı	2025	J Imaging Inform Med	Evaluation of Cone-Beam Computed Tomography Images with Artificial Intelligence	10.1007/s10278-025-01595-3	40588700	PMC13103123	https://doi.org/10.1007/s10278-025-01595-3	Turkey
S19	Bayrakdar	2025	Int J Comput Dent	Artificial intelligence in panoramic radiography interpretation: a glimpse into the state-of-the-art radiologic examination method	10.3290/j.ijcd.b6173229	40272192		https://doi.org/10.3290/j.ijcd.b6173229	Turkey
S20	Bennasar	2025	Diagnostics (Basel)	Integrating Machine Learning and Deep Learning for Predicting Non-Surgical Root Canal Treatment Outcomes Using Two-Dimensional Periapical Radiographs	10.3390/diagnostics15081009	40310439	PMC12025965	https://doi.org/10.3390/diagnostics15081009	Spain
S21	Gosavi	2025	Bioinformation	Comparison of AI-based radiographic interpretation versus endodontic specialists for identifying periapical lesions: Anstudy	10.6026/973206300214831	41907900	PMC13018384	https://doi.org/10.6026/973206300214831	India
S22	Hamdan	2025	BMC Oral Health	Evaluation of a no-code AI model for detecting periapical radiolucencies: impact of anatomical region on diagnostic performance	10.1186/s12903-025-07529-3	41419852	PMC12794265	https://doi.org/10.1186/s12903-025-07529-3	United States
S23	Hamdan	2025	Dentomaxillofac Radiol	The influence of a deep learning tool on the performance of oral and maxillofacial radiologists in the detection of apical radiolucencies	10.1093/dmfr/twae054	39656660		https://doi.org/10.1093/dmfr/twae054	United States
S24	Ibraheem	2025	Diagnostics (Basel)	Assessment of the Diagnostic Accuracy of Artificial Intelligence Software in Identifying Common Periodontal and Restorative Dental Conditions (Marginal Bone Loss, Periapical Lesion, Crown, Restoration, Dental Caries) in Intraoral Periapical Radiographs	10.3390/diagnostics15111432	40507004	PMC12154273	https://doi.org/10.3390/diagnostics15111432	Saudi Arabia
S25	Kim	2025	Dentomaxillofac Radiol	Enhancing panoramic dental imaging with AI-driven arch surface fitting: achieving improved clarity and accuracy through an optimal reconstruction zone	10.1093/dmfr/twaf006	39832267		https://doi.org/10.1093/dmfr/twaf006	Korea

row_id	first_author	year	journal	title	doi	pmid	pmcid	url	aff_country
S26	Liu	2025	BMC Oral Health	A comparative analysis of deep learning models for assisting in the diagnosis of periapical lesions in periapical radiographs	10.1186/s12903-025-06104-0	40420083	PMC12107815	https://doi.org/10.1186/s12903-025-06104-0	China
S27	Pekiner	2025	J Coll Physicians Surg Pak	A Deep Learning Approach for Detecting Periapical Lesions on Panoramic Radiographic Images	10.29271/jcpsp.2025.11.1461	41247689		https://doi.org/10.29271/jcpsp.2025.11.1461	Turkey
S28	Saber	2025	Sci Rep	Automated assessment of periapical health based on the radiographic periapical index using YOLOv8, YOLOv11, and YOLOv12 one-stage object detection algorithms	10.1038/s41598-025-21761-5	41115912	PMC12537981	https://doi.org/10.1038/s41598-025-21761-5	Egypt
S29	Szabó	2025	Diagnostics (Basel)	Deep Learning-Based Periapical Lesion Detection on Panoramic Radiographs	10.3390/diagnostics15040510	40002661	PMC11854226	https://doi.org/10.3390/diagnostics15040510	Hungary
S30	Chau	2024	J Dent	A novel AI model for detecting periapical lesion on CBCT: CBCT-SAM	10.1016/j.jdent.2024.105526	39667487		https://doi.org/10.1016/j.jdent.2024.105526	China
S31	Chen	2024	J Endod	Leveraging Pretrained Transformers for Efficient Segmentation and Lesion Detection in Cone-Beam Computed Tomography Scans	10.1016/j.joen.2024.07.012	39097163	PMC11471365	https://doi.org/10.1016/j.joen.2024.07.012	
S32	Hirata	2024	J Imaging Inform Med	Development of Periapical Index Score Classification System in Periapical Radiographs Using Deep Learning	10.1007/s10278-024-01360-y	39671050	PMC12572563	https://doi.org/10.1007/s10278-024-01360-y	Thailand
S33	Liu	2024	Sci Rep	Periapical lesion detection in periapical radiographs using the latest convolutional neural network ConvNeXt and its integrated models	10.1038/s41598-024-75748-9	39455655	PMC11511854	https://doi.org/10.1038/s41598-024-75748-9	China
S34	Rampf	2024	Eur J Dent Educ	Radiographical diagnostic competences of dental students using various feedback methods and integrating an artificial intelligence application-A randomized clinical trial	10.1111/eje.13028	39082447		https://doi.org/10.1111/eje.13028	Germany
S35	Viet	2024	Oral Radiol	Comparing the accuracy of two	10.1007/s11282-024-00759-1	38862834		https://doi.org/10.1007/s11282-024-00759-1	Vietnam

row_id	first_author	year	journal	title	doi	pmid	pmcid	url	aff_country
				machine learning models in detection and classification of periapical lesions using periapical radiographs					
S36	van Nistelrooij	2024	BMC Oral Health	Combining public datasets for automated tooth assessment in panoramic radiographs	10.1186/s12903-024-04129-5	38532414	PMC10964594	https://doi.org/10.1186/s12903-024-04129-5	Netherlands
S37	Fu	2023	J Dent Res	Clinically Oriented CBCT Periapical Lesion Evaluation via 3D CNN Algorithm	10.1177/00220345231201793	37968798		https://doi.org/10.1177/00220345231201793	China
S38	Hadzic	2023	J Clin Med	Evaluating a Periapical Lesion Detection CNN on a Clinically Representative CBCT Dataset-A Validation Study	10.3390/jcm13010197	38202204	PMC10779652	https://doi.org/10.3390/jcm13010197	Austria
S39	Huang	2023	J Endod	Uncertainty-based Active Learning by Bayesian U-Net for Multi-label Cone-beam CT Segmentation	10.1016/j.joen.2023.11.002	37979653	PMC10842728	https://doi.org/10.1016/j.joen.2023.11.002	
S40	Lee	2023	J Endod	An Endodontic Forecasting Model Based on the Analysis of Preoperative Dental Radiographs: A Pilot Study on an Endodontic Predictive Deep Neural Network	10.1016/j.joen.2023.03.015	37019378		https://doi.org/10.1016/j.joen.2023.03.015	Korea
S41	Ari	2022	Diagnostics (Basel)	Automatic Feature Segmentation in Dental Periapical Radiographs	10.3390/diagnostics12123081	36553088	PMC9777016	https://doi.org/10.3390/diagnostics12123081	Turkey
S42	Kirnbauer	2022	J Endod	Automatic Detection of Periapical Osteolytic Lesions on Cone-beam Computed Tomography Using Deep Convolutional Neuronal Networks	10.1016/j.joen.2022.07.013	35952897		https://doi.org/10.1016/j.joen.2022.07.013	Austria
S43	Qian	2022	Hua Xi Kou Qiang Yi Xue Za Zhi	Use and performance of artificial intelligence applications in the diagnosis of chronic apical periodontitis based on cone beam computed tomography imaging	10.7518/hxkq.2022.05.011	38596979	PMC9588865	https://doi.org/10.7518/hxkq.2022.05.011	China
S44	Moidu	2021	Clin Oral Investig	Deep learning for categorization of endodontic lesion based on radiographic periapical index scoring system	10.1007/s00784-021-04043-y	34213664		https://doi.org/10.1007/s00784-021-04043-y	India
S45	Orhan	2020	Int Endod J	Evaluation of artificial intelligence	10.1111/iej.13265	31922612		https://doi.org/10.1111/iej.13265	Turkey

row_id	first_author	year	journal	title	doi	pmid	pmcid	url	aff_country
				for detecting periapical pathosis on cone-beam computed tomography scans					
S46	Setzer	2020	J Endod	Artificial Intelligence for the Computer-aided Detection of Periapical Lesions in Cone-beam Computed Tomographic Images	10.1016/j.joen.2020.03.025	32402466		https://doi.org/10.1016/j.joen.2020.03.025	

S1b. Inclusion and coding provenance

row_id	first_author	included_in_denominator	exclusion_reason	exclusion_quote	coding_level	access_status	retrieval_route
S01	Adıgüzel	True			full_text	full_text_obtained	author_supplied_pdf
S02	Akyüz	True			full_text	full_text_obtained	author_supplied_pdf
S03	Almutairi	True			full_text	full_text_obtained	author_supplied_pdf
S04	Bubna	True			full_text	full_text_obtained	author_supplied_pdf
S05	Erkal	True			full_text	pmc_full_text	PubMed MCP get_full_text_article (PMC13383545)
S06	Hassanein	True			full_text	pmc_full_text	PubMed MCP get_full_text_article (PMC12937961)
S07	Kunnath Menon	True			full_text	pmc_full_text	PubMed MCP get_full_text_article (PMC12939923)
S08	Lee	True			full_text	full_text_obtained	author_supplied_pdf
S09	Marwaha	True			full_text	full_text_obtained	author_supplied_pdf
S10	Mayer	True			full_text	pmc_full_text	PubMed MCP get_full_text_article (PMC13125990)
S11	Obuchowicz	True			full_text	pmc_full_text	PubMed MCP get_full_text_article (PMC12898856)
S12	Sadr	True			full_text	full_text_obtained	author_supplied_pdf
S13	Scarano	True			full_text	pmc_full_text	PubMed MCP get_full_text_article (PMC13301822)
S14	Shahmaleki	True			full_text	full_text_obtained	author_supplied_pdf
S15	van Nistelrooij	True			full_text	pmc_full_text	PubMed MCP get_full_text_article (PMC13134024)
S16	Öksüzoğlu	True			full_text	pmc_full_text	PubMed MCP get_full_text_article (PMC13159272)
S17	Allihaibi	True			full_text	pmc_full_text	PubMed MCP get_full_text_article (PMC12254525)
S18	Arı	True			full_text	full_text_obtained	author_supplied_pdf
S19	Bayrakdar	True			full_text	full_text_obtained	author_supplied_pdf
S20	Bennasar	True			full_text	pmc_full_text	PubMed MCP get_full_text_article (PMC12025965)
S21	Gosavi	True			full_text	pmc_full_text	PubMed MCP get_full_text_article (PMC13018384)

row_id	first_author	included_in_denominator	exclusion_reason	exclusion_quote	coding_level	access_status	retrieval_route
S22	Hamdan	True			full_text	pmc_full_text	PubMed MCP get_full_text_article (PMC12794265)
S23	Hamdan	True			full_text	full_text_obtained	author_supplied_pdf
S24	Ibraheem	True			full_text	pmc_full_text	PubMed MCP get_full_text_article (PMC12154273)
S25	Kim	False	Method paper on 3D U-Net panoramic reconstruction evaluated by IoU, contrast and pixel values; reports no apical-periodontitis detection accuracy (zero occurrences of sensitivity or specificity in the full text).	Based on the original tooth with an IoU of 1, the 2 methods were compared by evaluating the IoU of the teeth generated from panoramic views at 5 different reconstruction thickness levels (5, 10, 15, 20, and 25 pixels).	full_text	full_text_obtained	author_supplied_pdf
S26	Liu	True			full_text	pmc_full_text	PubMed MCP get_full_text_article (PMC12107815)
S27	Pekiner	True			full_text	full_text_obtained	author_supplied_pdf
S28	Saber	True			full_text	pmc_full_text	PubMed MCP get_full_text_article (PMC12537981)
S29	Szabó	True			full_text	pmc_full_text	PubMed MCP get_full_text_article (PMC11854226)
S30	Chau	True			full_text	full_text_obtained	author_supplied_pdf
S31	Chen	True			full_text	pmc_full_text	PubMed MCP get_full_text_article (PMC11471365)
S32	Hirata	True			full_text	full_text_obtained	author_supplied_pdf
S33	Liu	True			full_text	pmc_full_text	PubMed MCP get_full_text_article (PMC11511854)
S34	Rampf	False	Randomised trial of teaching methods; unit of analysis is dental students, and the paper states no AI apical-periodontitis accuracy comparison was possible.	The detection of apical periodontitis by using the AI was not available at the time of study conduct and hence no further accuracy comparison was possible.	full_text	full_text_obtained	author_supplied_pdf
S35	Viet	True			full_text	full_text_obtained	author_supplied_pdf
S36	van Nistelrooij	True			full_text	pmc_full_text	PubMed MCP get_full_text_article (PMC10964594)
S37	Fu	True			full_text	full_text_obtained	author_supplied_pdf
S38	Hadzic	True			full_text	pmc_full_text	PubMed MCP get_full_text_article (PMC10779652)
S39	Huang	True			full_text	pmc_full_text	PubMed MCP get_full_text_article (PMC10842728)
S40	Lee	True			full_text	full_text_obtained	author_supplied_pdf
S41	Ari	True			full_text	pmc_full_text	PubMed MCP get_full_text_article (PMC9777016)
S42	Kirnbauer	True			full_text	full_text_obtained	author_supplied_pdf
S43	Qian	True			full_text	full_text_obtained	author_supplied_pdf
S44	Moidu	True			full_text	full_text_obtained	author_supplied_pdf
S45	Orhan	True			full_text	full_text_obtained	author_supplied_pdf
S46	Setzer	True			full_text	full_text_obtained	author_supplied_pdf

S1c. Reference standard

row_id	first_author	input_modality	reference_standard_abstract_level	reference_standard_upgraded	same_image_or_unstated	ref_quote_verbatim	coding_note	quote_splice_note
S01	Adıgüzel	IOPA	Human reading of same image	human_reading_same_image	True	The reference standard was established through independent evaluation of all radiographs by two experienced clinicians who were blinded to the model outputs.	Abstract explicitly states reference standard was established by two clinicians reading the same 2D periapical radiographs, explicitly without CBCT confirmation. Disagreements resolved by consensus; interobserver agreement reported as Cohen's kappa 0.88. No mention of lesion size or anatomical region subgroup analyses, and no external validation cohort described (single retrospective dataset from one university clinic).	
S02	Akyüz	IOPA	Human reading of same image	human_reading_same_image	True	Two endodontists independently evaluated all radiographs.	Abstract states expert endodontists served as reference standard reading the same periapical radiographs; n_annotators and clinical/3D access not specified beyond "endodontists" (implied at least two given inter-rater kappa, but count not explicitly stated).	
S03	Almutairi	IOPA	Human reading of same image	human_reading_same_image	True	All radiographs were reviewed independently on a calibrated 24-inch LCD monitor (1,920 × 1,080 resolution) under standardized lighting in a quiet environment without knowledge of the ChatGPT or general dentists' assessments.	Abstract states reference standard was set by endodontic specialists reading the same periapical radiographs, compared against a panel of general dentists and ChatGPT-5. No details on number of annotators, agreement statistics, 3D/clinical access, external validation cohort, or size/region subgroup analyses are given in the abstract.	
S04	Bubna	IOPA	Human reading of same image	human_reading_same_image	True	This categorization was performed by an endodontic specialist with over ten years of clinical experience, who individually evaluated each image according to the predefined criteria and recorded the corresponding tooth whenever an alteration was observed.	Abstract states a single calibrated endodontic specialist provided the reference diagnosis (reference standard from same-image human reading); performance stratified by "diagnostic difficulty" but not explicitly by lesion size or region, so those	

row_id	first_author	input_modality	reference_standard_abstract_level	reference_standard_upgraded	same_image_or_unstated	ref_quote_verbatim	coding_note	quote_splice_note
							moderators are coded no_evidence_in_passages. No interannotator agreement reported since only one annotator mentioned. No external validation cohort described.	
S05	Erkal	IOPA	Other / mixed	human_reading_same_image	True	Lesion masks were manually delineated by 2 endodontists (each with > 5 years of clinical experience) using ImageJ (NIH, Bethesda, MD, USA).	Reference standard established by 2 endodontists manually delineating lesion masks and assigning PAI scores on the same periapical radiographs (IOPA), with disagreements resolved by joint review to reach consensus. No CBCT, histopathology, or surgical outcome verification mentioned; the reference standard is consensus expert reading of the same 2D image. External validation not performed - authors explicitly call for future multicenter external validation. No lesion-size or anatomical-region subgroup moderator analyses evidenced in passages.	
S06	Hassanein	IOPA	Human reading of same image	human_reading_same_image	True	Reference Standard (Expert Consensus) The reference standard was established through the consensus of three board-certified oral and maxillofacial radiologists, each with over 10 years of clinical and academic experience in diagnostic dental imaging.	Reference standard was three-expert (radiologist) consensus reading of the same 2D periapical radiographs; discrepant cases resolved by consensus discussion. Because it was consensus-based, formal inter-/intra-rater reliability testing was explicitly stated as not applicable (no kappa reported for the reference standard itself, though Cohen's kappa/PABAK were used to measure agreement between ChatGPT-5 and the consensus, not inter-annotator agreement among the experts). Experts appear limited to 2D radiographic reading (no CBCT/histopath confirmation mentioned; discussion explicitly notes absence of CBCT/histopathology as a limitation). Region/anatomical	

row_id	first_author	input_modality	reference_standard_abstract_level	reference_standard_upgraded	same_image_or_unstated	ref_quote_verbatim	coding_note	quote_splice_note
							localization (arch, tooth position) was analysed as a moderator of agreement; no explicit lesion-size subgroup analysis found in passages.	
S07	Kunnath Menon	MIXED	Human reading of same image	human_reading_same_image	True	The diagnostic consensus was specified as the "ground truth".	Reference standard was established by consensus of two specialist evaluators reading the same radiographs (2D images), with a third reviewer for disagreements; radiographs excluded if no consensus. No mention of CBCT, histopathology, or clinical/surgical outcome as reference standard. No inter-annotator agreement statistic (e.g., kappa) reported in passages, only description of calibration/consensus process. No external validation cohort described; no lesion-size or region subgroup moderator analysis evidenced.	
S08	Lee	IOPA	Human reading of same image	human_reading_same_image	True	The reference standard was defined as the consensus PAI score determined by two endodontists.	Abstract states PRs and metadata collected from a single institution, reference standard set by consensus of two endodontists reading the PRs (same modality). Validation used stratified 5-fold cross-validation (internal split), no external cohort mentioned. No inter-annotator agreement value reported. No lesion size or tooth region subgroup analysis mentioned; tooth location/number used only as input metadata, not as a moderator analysis of performance.	
S09	Marwaha	IOPA	Histopathology	histopathology	False	The reference standard of lesion classification was the histopathology of the tissue samples collected at periapical surgery or removal.	Reference standard explicitly = histopathological verification from endodontic treatment/periapical surgery; clinicians (3 endodontists) also independently evaluated same radiographs as comparator, not as reference standard.	quote reproduces the contiguous fragment as printed; the sentence is broken across a page boundary

row_id	first_author	input_modality	reference_standard_abstract_level	reference_standard_upgraded	same_image_or_unstated	ref_quote_verbatim	coding_note	quote_splice_note
							Internal train/val/test split (80/10/10) reported, no external cohort. Lesion size moderator explicitly analysed (<3mm vs others); region (anatomic site) moderator not mentioned in abstract.	by a running head in the published PDF text layer
S10	Mayer	IOPA	Human reading of same image	human_reading_same_image	True	All metrics were calculated for each human examiner (1, 2, and 3) and for the AI system against the gold standard, defined as agreement of at least two human examiners.	Reference standard = consensus of ≥2 of 3 human evaluators reading the same IOPA/FMX images (medium- and high-resolution sets), blinded to AI results. No CBCT, histopathology, or clinical outcome verification mentioned. External validation not described beyond internal institutional database (255 FMX screened); no explicit train/test split or multicentre statement in passages, so coded as none_stated. No lesion-size or region subgroup moderator analyses mentioned in passages, only pathology-type and resolution comparisons.	
S11	Obuchowicz	IOPA	CBCT verification	CBCT_verification	False	CBCT served as the reference standard, and all cases in this subset were considered true positives (CBCT label = 1).	Reference standard labels (onlyCBCT vs noLesion) were derived from CBCT findings, with ROI annotation/labeling performed on RVG images based on CBCT. A separate reader study (3 radiologists) assessed visual detectability on RVG blinded to CBCT, showing poor inter-reader agreement; this is a comparison arm, not the reference standard source. No external validation reported (explicitly stated as a limitation: absence of external validation and multicenter data). No lesion size or anatomical region moderator analysis evidenced.	
S12	Sadr	PANORAMIC	Not stated in abstract	not_stated	True	details regarding the labeling process and the qualifications of the annotators have not been disclosed	Author decision: recoded from other_mixed to not_stated. Justification verbatim from the paper: "details regarding the labeling process and the	

row_id	first_author	input_modality	reference_standard_abstract_level	reference_standard_upgraded	same_image_or_unstated	ref_quote_verbatim	coding_note	quote_splice_note
							qualifications of the annotators have not been disclosed". Labels originate from the public DenTex dataset whose annotation process the paper states is undisclosed.	
S13	Scarano	PANORAMIC	Not stated in abstract	human_reading_same_image	True	The publicly released periapical lesion detection dataset [] consists of 3926 images with bounding-box annotations established by three experienced dentists (each with more than 5 years of clinical practice), classifying periapical lesions based on the Periapical Index (PAI) scoring system.	Reference standard for periapical lesion bounding boxes was established by three experienced dentists reading panoramic radiographs directly (same modality as model input) using PAI scoring; no CBCT/histopathology confirmation mentioned. A separate tooth segmentation dataset (DualLabel) had 5 annotators with a senior reviewer, but that pertains to tooth segmentation not lesion reference standard, so annotator count/expertise fields reflect the lesion detection dataset specifically.	
S14	Shahmaleki	PANORAMIC	Not stated in abstract	human_reading_same_image	True	Periapical lesions were diagnosed solely on the basis of panoramic radiographic findings assessed by experienced oral and maxillofacial radiologists, and no clinical, histopathological, or longitudinal follow-up confirmation was available.	Author decision: recoded from not_stated_in_abstract_fulltext_unavailable to human_reading_same_image on the supplied full text.	
S15	van Nistelrooij	MIXED	Human reading of same image	human_reading_same_image	True	For the second round of annotations, each annotated tooth was assessed by at least 3 clinicians independently and the clinician assessments (present/absent) were aggregated using the Dawid-Skene model.This is a probabilistic method that estimates the presence of a dental finding by weighting each clinician's assessment according to their inferred diagnostic accuracy.	Reference standard for dental findings (which include periapical lesion presence among 10 findings) was established via multiple (≥3 of 10 trained) clinicians independently reading the same 2D dental radiographs, aggregated using the Dawid-Skene probabilistic model. Annotators had no patient information/clinical access beyond the radiograph itself. No CBCT, histopathology, or surgical outcome verification mentioned. Agreement with consensus during training phase was used for annotator selection but no quantitative inter-annotator agreement value (e.g., kappa) for the actual	

row_id	first_author	input_modality	reference_standard_abstract_level	reference_standard_upgraded	same_image_or_unstated	ref_quote_verbatim	coding_note	quote_splice_note
							study annotations was given in passages. External validation: study mentions a possible future third-center external validation, but none was performed (none_stated). No lesion-size or anatomical-region moderator subgroup analysis evidenced.	
S16	Öksüz oğlu	CBCT	Human reading of same image	human_reading_same_image	True	Initial segmentations were performed by a research assistant (H.Ö.) with 1 year of experience in Oral and Maxillofacial Radiology and 3 years in Endodontics, then re-checked by H.Ö. and subsequently reviewed with a senior endodontist (Ö.T.) with 28 years of clinical experience; in cases of disagreement, the senior endodontist made the final decision, and consensus was achieved for all ground-truth segmentations.	Reference standard was manual segmentation on the same CBCT images the model consumes (human_reading_same_image), performed by two annotators with expert consensus review; explicitly no inter-examiner agreement metric was reported (authors state this as a limitation). Test set n=14 scans / 36 lesions represents an internal held-out split, not external multicentre validation. Lesion size analysed as moderator (FP/FN heavily tied to small lesion volume, discussion of small vs large lesion performance); no explicit region-based (anterior/posterior, maxilla/mandible) moderator analysis found in passages.	
S17	Allihaibi	IOPA	CBCT verification	CBCT_verification	False	Given these prior limitations and the need for further validation, the aim of this study was to assess the performance of Diagnocat in detecting apical radiolucencies on periapical radiographs of teeth without prior root canal treatment, using CBCT as the reference standard.	Assessments were conducted blinded to clinical information, Diagnocat results, and CBCT findings, so clinicians reading the PA image did not have 3D/clinical access at the time of assessment; CBCT itself served as the independent reference standard performed in prior prospective studies.	
S18	Arı	CBCT	Human reading of same image	human_reading_same_image	True	CBCT axial slice images were labeled using CranioCatch labeling software (Eskişehir, Türkiye).	Reference standard derived from manual segmentation labeling directly on the same CBCT images the model analyzes (same modality as input), so coded as human_reading_same_image. Annotator had 3D access since labeling was performed on CBCT images	

row_id	first_author	input_modality	reference_standard_abstract_level	reference_standard_upgraded	same_image_or_unstated	ref_quote_verbatim	coding_note	quote_splice_note
							themselves. No details on number/expertise of annotators, inter-annotator agreement, external validation, or size/region subgroup analyses stated in abstract.	
S19	Bayrakdar	PANORAMIC	Not stated in abstract	human_reading_same_image	True	PRs were manually segmented using CranioCatch labeling software (Eskisehir, Turkey) by dental practitioners of varying experience levels for the various dental conditions and the anatomical landmarks shown in Table 1.	Abstract only states panoramic radiographs were "labeled" for 33 conditions including periapical lesions, implying human annotation on the same panoramic images, but does not specify number/expertise of annotators, agreement statistics, or validation cohort details.	
S20	Bennasar	IOPA	Not stated in abstract	surgical_or_clinical_outcome	False	All periapical radiographs, 108 in total since due to geometric distortion or anatomical noise 11 were excluded, used for DL model training were preoperative images obtained prior to NSRCT, labeled based on post-treatment follow-up outcomes (healed: 0/not healed: 1) after a minimum of 9 years (and).	Reference standard labels (healed/not healed) were assigned based on clinical/radiographic follow-up outcome at minimum 9 years post-NSRCT, i.e., longitudinal clinical outcome, not a same-image human reading of the pretreatment radiograph. AnotIA segmentation was used only to guide model attention to clinically relevant areas, not as the outcome label source, so it does not change the reference_standard classification. No information on number/expertise of annotators, inter-annotator agreement, 3D/clinical access, external validation type, or lesion-size/region moderator analyses was present in the supplied passages.	
S21	Gosavi	IOPA	Human reading of same image	CBCT_verification	False	Reference standard establishment: The reference standard (ground truth) was established through comprehensive evaluation combining: (1) CBCT imaging performed within 30 days of periapical radiograph showing three-dimensional confirmation of periapical bone changes; (2) clinical findings including percussion sensitivity, palpation tenderness, sinus tract presence, and pulp vitality testing; (3) consensus diagnosis by two senior endodontists not participating in the evaluation phase, based on integrated CBCT and clinical data.	Reference standard combined CBCT confirmation, clinical findings, and consensus of two senior endodontists (CBCT_verification best fits since CBCT imaging is the core 3D confirmation component, combined with clinical/consensus criteria - classified as CBCT_verification given explicit CBCT-based reference	

row_id	first_author	input_modality	reference_standard_abstract_level	reference_standard_upgraded	same_image_or_unstated	ref_quote_verbatim	coding_note	quote_splice_note
							standard construction). Specialists evaluating IOPA images were blinded to CBCT/clinical info; inter-rater agreement reported among specialists and between AI and specialists. No explicit lesion-size or region subgroup moderator analysis found in passages. External validation not performed (single institutional dataset, limitation noted).	
S22	Hamdan	IOPA	CBCT verification	CBCT_verification	False	The presence or absence of periapical radiolucent lesions was established using the CBCT volumes and corresponding radiology reports as the reference standard.	Reference standard explicitly CBCT + radiology reports. Manual bounding-box annotation on IOPA images was for training data (lesion presence per CBCT-confirmed cases), not the reference standard itself. No inter-annotator agreement reported; no external validation stated (limitation notes absence of external testing). Region (anatomical region) is the study's primary moderator per title; lesion size not addressed in passages.	
S23	Hamdan	IOPA	CBCT verification	CBCT_verification	False	If a radiology report indicated the presence of an apical radiolucency, the primary investigator re-examined the corresponding CBCT volume for further analysis.	Reference standard for the 68 periapical radiographs was confirmed via CBCT, per abstract. The four oral radiologists were readers evaluating with/without AI assistance, not the original annotators establishing reference standard; CBCT confirmation is the stated reference standard. Annotators (readers) had clinical access to CBCT-confirmed cases as test subjects, but it's unclear if they had 3D access during reading itself - marked yes based on radiologist expertise/role in the cross-over study, though this refers to reader condition not reference standard annotation.	
S24	Ibraheem	IOPA	Human reading of same image	human_reading_same_image	True	These two experts independently labeled all the predefined parameters for the provided IOPARs (200 each) under standardized conditions (standardized pixels and dimly lit rooms) [].	Reference standard established by two oral	

row_id	first_author	input_modality	reference_standard_abstract_level	reference_standard_upgraded	same_image_or_unstated	ref_quote_verbatim	coding_note	quote_splice_note
							radiologists independently labeling IOPAR images (same modality as AI input), with a third radiologist reconfirming and resolving disagreements; inter-observer reliability (Cohen's kappa 0.95) reported between the two primary annotators. No mention of CBCT, histopathology, or clinical/surgical outcome confirmation, nor of lesion size or anatomical region subgroup analyses, nor of external/multicentre validation.	
S25	Kim	CBCT	Other / mixed	not_applicable_no_detection_claim		Based on the original tooth with an IoU of 1, the 2 methods were compared by evaluating the IoU of the teeth generated from panoramic views at 5 different reconstruction thickness levels (5, 10, 15, 20, and 25 pixels).	Author decision: excluded from the denominator; retained in Table S1 so the exclusion is auditable.	
S26	Liu	IOPA	Not stated in abstract	human_reading_same_image	True	The gold standard for PLs diagnosis during model training and validation was established by three oral radiologists with over 15 years of experience.	Reference standard established by 3 oral radiologists manually annotating ROIs on the same periapical radiographs (IOPA) using LabelImg; consensus of at least 2/3 required. No mention of CBCT, histopathology, or clinical outcome verification.	
S27	Pekiner	PANORAMIC	Not stated in abstract	human_reading_same_image	True	A dental student (MYP), who had previously undergone segmentation training to identify periapical lesions, along with two of the authors (HY and AGOT), labelled the patient images using the CranioCatch programme. Two oral and maxillofacial radiologists (FMNP and GK) examined the labelled periapical lesions.	Author decision: recoded from not_stated_in_abstract_fulltext_unavailable to human_reading_same_image on the supplied full text. Labelling and verification both performed on the panoramic images the model consumes.	
S28	Saber	IOPA	Human reading of same image	human_reading_same_image	True	Two experts annotated the whole dataset independently, and the third was consulted in their presence to reach a consensus in case there was a scoring disagreement.	Reference standard established via manual PAI scoring and bounding-box annotation on the same IOPA radiographs by two experts with third-expert consensus for disagreements; no CBCT/histopathology/surgical confirmation mentioned. External validation performed on 70 new images from a different clinic/device but same general population/country context (not stated to be a different country), coded as	

row_id	first_author	input_modality	reference_standard_abstract_level	reference_standard_upgraded	same_image_or_unstated	ref_quote_verbatim	coding_note	quote_splice_note
							external_same_country given no country difference is mentioned but a different clinical facility/device was used.	
S29	Szabó	PANORAMIC	Human reading of same image	human_reading_same_image	True	The ground truth was determined during personal sessions by two dentomaxillofacial radiologists, one with more than ten and one with more than thirty years of experience.	Reference standard was established by two experienced dentomaxillofacial radiologists reading the same panoramic radiographs (same modality as model input), with consensus resolution of disagreements. This is distinct from the three independent observers (student, general dentist, radiologist) who separately evaluated the PRs for the diagnostic performance comparison. The study explicitly notes absence of histopathological confirmation as a limitation, confirming reference standard was human reading, not histopathology. Some candidate sentences (Pauwels et al., Ekert et al.) refer to other studies cited in Discussion, not this study's own methodology, and were not used as the primary quote.	
S30	Chau	CBCT	Human reading of same image	human_reading_same_image	True	Periapical lesions in the CBCT scans were subsequently manually labelled in red by CKK and AA with ITK-SNAP [citation omitted].	Reference standard derived from manual segmentation on CBCT (same modality model uses) by trained operator, validated by maxillofacial radiologist - coded as human_reading_same_image since CBCT is the input modality itself, not an independent verification modality. n_annotators=2 inferred directly from named roles (operator + radiologist); no kappa/agreement value reported in abstract.	
S31	Chen	CBCT	Human reading of same image	human_reading_same_image	True	For purposes of this study, clinician experts in CBCT segmentation labeled every entire three-dimensional volume differentiating between “lesion”, “restorative material”, “bone”, “tooth structure”, and “background” following protocols established in previous studies as control.	Reference standard was established by clinician experts manually labeling the full 3D CBCT volumes (lesion, restorative material, bone, tooth structure, background) using a semi-automatic ITK-SNAP protocol with	

row_id	first_author	input_modality	reference_standard_abstract_level	reference_standard_upgraded	same_image_or_unstated	ref_quote_verbatim	coding_note	quote_splice_note
							manual review/revision - i.e., human reading of the same CBCT modality the model consumes. The paper explicitly discusses limitations of inter/intra-examiner reliability of CBCT interpretation and that low-PAI lesions were not labeled, but no formal inter-annotator agreement statistic (e.g., kappa) is reported in the passages. No external validation cohort described - only train/test split within the same dataset (internal_split assumed absent explicit statement, so coded none_stated). No lesion-size or region subgroup moderator analyses evident in passages.	
S32	Hirata	IOPA	Human reading of same image	human_reading_same_image	True	Images were manually annotated with bounding boxes covering PRAs by a consensus of two experts who are an oral and maxillofacial radiologist and an endodontist, both with 10 years of experience.	Abstract states PRAs were "scored by experts" using PAI on periapical radiographs (same modality as model input), consistent with human_reading_same_image. No details on number of annotators, agreement statistics, 3D/clinical access, or external validation cohort are given in the abstract.	
S33	Liu	IOPA	Not stated in abstract	human_reading_same_image	True	A consensus among at least two of the three doctors was requisite for validating the presence of periapical lesions in the images.	Reference standard established by consensus (≥2 of 3) of experienced oral radiologists reading the same periapical radiographs, annotating ROIs with LabelImg. No CBCT/histopathology/surgical confirmation mentioned. Passages explicitly note lack of external validation ("Future research should..."; single-center dataset limitation) and call for collaboration across institutions - interpreted as no external validation performed (none_stated). No kappa/agreement statistic reported. No mention of 3D imaging or clinical access. No	

row_id	first_author	input_modality	reference_standard_abstract_level	reference_standard_upgraded	same_image_or_unstated	ref_quote_verbatim	coding_note	quote_splice_note
							lesion size or region subgroup analysis evidenced.	
S34	Rampf	IOPA	Other / mixed	human_reading_same_image	True	Only bitewing images exhibiting at least six complete dental crowns and periapical images exhibiting at least three complete teeth (crown and roots) were included and independently evaluated by two dental experts with decades of clinical experience.	Author decision: excluded from the denominator; retained in Table S1 so the exclusion is auditable.	
S35	Viet	IOPA	Human reading of same image	human_reading_same_image	True	9728 PR were assessed to detect and classify periapical lesions based on the PAI scoring system by 3 experienced dentists.	Abstract states reference diagnosis was made by experienced dentists on periapical radiographs (same modality as model input), using PAI scoring. Dataset split into training/validation/testing (internal split) with no mention of external validation cohort. Region moderator analysed explicitly (anterior, premolar, molar comparison stated as no clear difference). No mention of lesion size analysis or inter-annotator agreement statistics.	
S36	van Nistelrooij	PANORAMIC	Other / mixed	other_mixed	False	More specifically, each diagnosed tooth is annotated within a bounding box and labeled as impacted, early caries, deep caries, and/or periapical lesion.	Reference standard is derived from two public datasets (OdontoAI, DENTEX) with pre-existing annotations on panoramic radiographs; the provenance/expertise of original annotators is not described in the supplied passages, and the paper notes inconsistency/quality issues in the DENTEX annotations (missing diagnoses, misdiagnoses), suggesting mixed/uncertain annotation quality rather than a clearly defined single reference standard. No inter-annotator agreement, annotator count/expertise, or 3D/clinical access is stated. Evaluation used a five-fold cross-validation and held-out test splits (internal split), not external validation across sites/countries.	
S37	Fu	CBCT	Human reading of same image	other_mixed	False	The gold standard diagnoses of AP were verified by 2 endodontists (W.T.F. and N.L.) using pathology and/or CBCT reports.	Methods state the reference standard combined pathology reports (21 cases) with CBCT reports and	quote reproduces the contiguous fragment

row _id	first_a uthor	input_m odality	reference_standar d_abstract_level	reference_standar d_upgraded	same_image_ or_unstated	ref_quote_verbatim	coding_note	quote_spl ice_note
							health records; a composite incorporating the index modality rather than a reading of it alone.	as printed; the sentence is interrupted by a figure caption in the published PDF text layer
S38	Hadzic	CBCT	Not stated in abstract	human_reading_sa me_image	True	The ground-truth detection of PALs was performed by three investigators (two senior oral surgeons with >15 years of experience and one junior dentist), who did an initial round of lesion detection separately from each other on the whole dataset.	Reference standard derived from consensus reading of CBCT images (same modality model consumes) by three investigators using Estrela PAI scoring; second round consensus established expert reference standard. Lesion diameter-based PAI scoring used as size moderator (five categories). No region (anterior/posterior/jaw) moderator analysis found in passages. External validation type coded as internal_split since evaluation was on a new dataset distinct from training set (144 training vs 195/196 test images), both presumably same-institution; no explicit statement of different country/multicentre.	
S39	Huang	CBCT	Human reading of same image	human_reading_sa me_image	True	All reviewers were calibrated and trained in CBCT analysis and segmentation and included two senior graduate students completing an endodontic honors program (NF, JBY), and an endodontist (FCS), who closely supervised the students during the segmentation process.	Reference standard established by clinician segmentation on same CBCT volumes model is applied to (human_reading_same_image), using ITK-SNAP semi-automatic segmentation followed by manual review/revision by trained/calibrated reviewers (2 endodontic honors students + supervising endodontist). No inter-annotator agreement statistic reported in passages. Annotators had full 3D CBCT access (same modality as model input) and clinical training, so coded 'yes' for 3D/clinical access. No external validation cohort stated (single institution/dataset, AL train/test split only) ->	

row_id	first_author	input_modality	reference_standard_abstract_level	reference_standard_upgraded	same_image_or_unstated	ref_quote_verbatim	coding_note	quote_splice_note
							none_stated. Lesion size discussed as moderator of Dice performance (lesion size vs Dice index), so yes. No region-based (anatomical location) subgroup analysis evidenced in passages, though authors suggest region-based AL as future work, not an analysis performed here.	
S40	Lee	IOPA	Surgical or clinical outcome	surgical_or_clinical_outcome	False	A database of single-root premolars that received endodontic treatment or retreatment by endodontists with presence of three-year outcome was prepared (n = 598).	Reference is three-year post-treatment outcome (PAI at follow-up or extraction), not a reading of the preoperative index radiograph; independent but prognostic rather than detection validation.	
S41	Ari	IOPA	Human reading of same image	human_reading_same_image	True	As a research assistant with 2 years of experience and a dento-maxillofacial radiologist with 12 years of experience performed labeling on periapical radiographs for different findings using CranioCatch annotation software (CranioCatch, Eskişehir, Turkey).	Reference standard established by manual labeling on the same periapical radiographs by a research assistant and a dento-maxillofacial radiologist using CranioCatch software; no CBCT, histopathology, or surgical outcome reference mentioned. Limitations sentence explicitly notes no external dataset used and lack of observers with different experience, supporting none_stated for external validation and no evidence for IAA.	
S42	Kirnbauer	CBCT	Not stated in abstract	human_reading_same_image	True	Such segmentation was followed by a manual verification and, if necessary, an adaption in the open-source software program ITKSNAP32 by an oral surgeon specializing in oral radiology.	Full text documents the labelling process: semi-automatic weighted-total-variation segmentation from operator-marked contours on the same CBCT volume, manually verified by an oral surgeon specializing in oral radiology, with PAL presence defined radiographically. Contradicts the prior 'not stated' coding, which rested on abstract silence.	
S43	Qian	CBCT	Human reading of same image	human_reading_same_image	True	从49例CBCT原始数据中随机抽取6个根尖病变，慢性根尖周炎的CBCT数据以DICOM格式导出，图像由4名主治医生先后2次（间隔2周）记录颌骨病变的位置并对于慢性根尖周炎病变区域进行识别同时使用Materialize Mimics Medical软件进行手动逐层分割。	Reference standard was manual segmentation of CBCT lesions by experienced clinicians using the same CBCT images the AI model	

row_id	first_author	input_modality	reference_standard_abstract_level	reference_standard_upgraded	same_image_or_unstated	ref_quote_verbatim	coding_note	quote_splice_note
							consumed (3D U-Net segmentation), coded as human_reading_same_image. Test set comparison implies internal train/test split; no external validation, cohort agreement, or size/region subgroup analysis mentioned in abstract.	
S44	Moidu	IOPA	Human reading of same image	human_reading_same_image	True	This was taken as the reference standard score.	Abstract states reference standard was pre-scoring of PRA by three endodontists using PAI scoring system on the same IOPA images the model uses. Data split into train/validation/test sets from same source (internal split). No mention of inter-annotator agreement, 3D/clinical access, or lesion size/region subgroup analysis in the abstract.	
S45	Orhan	CBCT	Human reading of same image	human_reading_same_image	True	Tomography data with periapical lesions were anonymized in DICOM format, and images were evaluated by an oral and maxillofacial radiologist (_I.S.B.) with 8 years of professional experience.	Reference standard for lesion presence/localization/volume derived from human observer determination and manual segmentation on the same CBCT images used as AI input. No annotator count, expertise, or agreement statistics reported in abstract. Annotator had access to CBCT (3D) images by definition since modality is CBCT.	
S46	Setzer	CBCT	Human reading of same image	human_reading_same_image	True	Clinician-dependent CBCT volume segmentation (referred to as clinician segmentation [CS]) was performed using ITKSNAP (open-source) following protocols established in previous studies23–25.	Reference standard derived from clinician-dependent segmentation of the same CBCT volumes used as model input; 5-fold cross validation described as internal split. No mention of number of annotators, agreement statistics, or size/region subgroup analyses in the abstract.	

S1d. Annotation provenance and reliability

row_id	first_author	n_annotators	annotator_expertise	annotator_had_3d_or_clinical_access	annotator_access_beyond_index_image	access_beyond_basis	annotator_access_quote	iaa_statistic_reported	iaa_pertains_to	iaa_pertains_to_own_labels	iaa_value	iaa_quote

row_id	first_author	n_annotators	annotator_expertise	annotator_had_3d_or_clinical_access	annotator_access_beyond_index_image	access_beyond_basis	annotator_access_quote	iaa_statistic_reported	iaa_pertains_to	iaa_pertains_to_own_labels	iaa_value	iaa_quote
S01	Adıgüzel	2	two experienced clinicians	no	no	reported as reading the index image only	Although three-dimensional imaging modalities such as CBCT may provide higher diagnostic sensitivity than periapical radiography, particularly for early or subtle lesions, CBCT confirmation was not available for the present dataset (3, 4).	yes_interobserver_statistic	Interobserver agreement for AP status among the two reference-standard clinicians	yes	0.88	Interobserver agreement for AP status was assessed using Cohen's kappa (κ) coefficient and indicated a high level of agreement (κ = 0.88).
S02	Akyüz	2	endodontists (each with more than 10 years of clinical experience)	no	no	reported as reading the index image only	The purpose of the present study was therefore to evaluate AI-assisted interpretation within the context of routine two-dimensional radiographic assessment rather than to establish an anatomical ground truth through advanced imaging.	yes_interobserver_statistic	inter-examiner agreement between the two expert endodontists	yes	0.95	Agreement between the expert endodontists was nearly perfect (κ 5 0.95, P , .001).
S03	Almutairi	2	two board-certified endodontists, each with more than 5 years of clinical experience in endodontic diagnosis and treatment	no	no	reported as reading the index image only	All radiographs were reviewed independently on a calibrated 24-inch LCD monitor (1,920 × 1,080 resolution) under standardized lighting in a quiet environment without knowledge of the ChatGPT or general dentists' assessments.	no				
S04	Bubna	1	endodontic specialist with over ten years of	no	no	reported as reading the index image only	This categorization was performed by an endodontic specialist with over ten years of clinical experience, who individually evaluated each image according to the predefined criteria and recorded the corresponding tooth whenever an alteration was observed.	yes_interobserver_statistic	intra-examiner agreement of the reference diagn	yes	greater than 0.90	To ensure reliability, all images were re-evaluated after a two-week interval to determine intra-examiner agreement using Cohen's Kappa coefficient, which yielded a value greater than 0.90, indicating excellent reproducibility.

row_id	first_author	n_annotators	annotator_expertise	annotator_had_3d_or_clinical_access	annotator_access_beyond_index_image	access_beyond_basis	annotator_access_quote	iaa_statistic_reported	iaa_pertains_to	iaa_pertains_to_own_labels	iaa_value	iaa_quote
			clinical experience						osis			
S05	Erkal	2	endodontists with >5 years of clinical experience	no	no	reported as reading the index image only		yes_interobserver_statistic	ground_truth_annotators	yes	Binary classification: κ = 0.812 (95% CI 0.698–0.926); Ordinal PAI: weighted κ = 0.918 (95% CI 0.867–0.969); Segmentation: Dice = 0.89 (95% CI 0.84–0.94)	For binary image-level classification (healthy = PAI 1 vs. diseased = PAI 2–5), agreement was excellent (κ = 0.812, 95% CI 0.698–0.926).
S06	Hassanein	3	Board-certified oral and maxillofacial radiologists, each with over	no	no	reported as reading the index image only		yes_interobserver_statistic	model_vs_human	no	Cohen's κ varied by parameter: overall abnormalit	Agreement between raters was assessed using Cohen's κ, supplemented by prevalence- and bias-adjusted κ (PABAK) where appropriate.

row_id	first_author	n_annotators	annotator_expertise	annotator_had_3d_or_clinical_access	annotator_access_beyond_index_image	access_beyond_basis	annotator_access_quote	iaa_statistic_reported	iaa_pertains_to	iaa_pertains_to_own_labels	iaa_value	iaa_quote
			10 years of clinical and academic experience								y detection $\kappa = 0.000$; arch $\kappa = 0.857$; lesion border characterization $\kappa = 0.127$ ($p < 0.001$); PABAK also computed where appropriate	
S07	Kunnath Menon	2	Two independent specialist evaluators with >8 years clinical experience (one with postgraduate training, one with endodontic/pedodontic depar	no	no	reported as reading the index image only		only_qualitative_consensus_statement	ground_truth_annotators			In the event of any discrepancy, a meeting was called for the specialist evaluators to discuss the disagreement and reach a diagnostic consensus.

row_id	first_author	n_annotators	annotator_expertise	annotator_had_3d_or_clinical_access	annotator_access_beyond_index_image	access_beyond_basis	annotator_access_quote	iaa_statistic_reported	iaa_pertains_to	iaa_pertains_to_own_labels	iaa_value	iaa_quote
			tment experience); third reviewer for tiebreaking									
S08	Lee	2	two endodontists	no	no	reported as reading the index image only	Each expert independently assessed the radiographs based on the Ørstavik criteria, which define PAI scores on a five-point scale.	no				
S09	Marwaha	3	three board-certified endodontists who had 8-15 years of clinical experience	no	no	reported as reading the index image only	Clinical history and histopathological results were masked to the eyes of the observers.	yes_interobserver_statistic	inter-observer agreement among endodontists	yes	0.612	clinicians was moderate (Fleiss' κ=0.612; 95% CI: 0.548-0.676)
S10	Mayer	3	experienced clinicians (three human evaluators/examiners)	no	no	reported as reading the index image only		yes_interobserver_statistic	ground_truth_annotators	yes	Cohen's kappa 0.27–0.79 (medium resolution) and 0.49–0.87 (high resolution) among three human evaluato	Inter-examiner agreement among the three human evaluators demonstrated substantial reliability across all pathological conditions (), with Cohen’s kappa values ranging from 0.27 to 0.79 in medium-resolution images, where the highest agreement was observed for missing teeth (mean κ = 0.79, 95% CI: 0.75-0.83) and the lowest for root resorption (mean κ = 0.27, 95% CI: 0.20-0.34).

row_id	first_author	n_annotators	annotator_expertise	annotator_had_3d_or_clinical_access	annotator_access_beyond_index_image	access_beyond_basis	annotator_access_quote	iaa_statistic_reported	iaa_pertains_to	iaa_pertains_to_own_labels	iaa_value	iaa_quote
											rs	
S11	Obuchowicz	3	radiologists (labeling based on CBCT); three radiologists for visual assessment reader study	yes	yes	non-CBCT input with reported 3D or clinical access		yes_interobserver_statistic	separate_reader_study	no	Quadratic-weighted Cohen's kappa ranged from -0.09 to 0.06 across reader pairs; Kendall's W = 0.015	Quadratic-weighted Cohen's κ values ranged from −0.09 to 0.06 across reader pairs, and overall concordance was negligible (Kendall's W = 0.015), thus indicating substantial variability in visual confidence scoring.
S12	Sadr							no				
S13	Scarano	3	three experienced dentists, each with more than 5 years of clinical practice	no	no	reported as reading the index image only		no				
S14	Shahmaleki	2	two dentomaxillofacial radiologists	no	no	reported as reading the index image only	Periapical lesions were diagnosed solely on the basis of panoramic radiographic findings assessed by experienced oral and maxillofacial radiologists, and no clinical, histopathological, or longitudinal follow-up confirmation was available.	yes_interobserver_statistic	interobserver and intraobserver agreement between the two radiologists on periapical	yes	97.2%	Two radiologists independently reviewed all radiographs, and retrospective evaluation demonstrated a high absolute percentage interobserver agreement rate of 97.2%, with absolute intraobserver agreement rates of 96.4% and 98.3% for the first and second observers, respectively, demonstrating high descriptive consistency in the reference standard.

row_id	first_author	n_annotators	annotator_expertise	annotator_had_3d_or_clinical_access	annotator_access_beyond_index_image	access_beyond_basis	annotator_access_quote	iaa_statistic_reported	iaa_pertains_to	iaa_pertains_to_own_labels	iaa_value	iaa_quote
									lesion diagnosis			
S15	van Nistelrooij	10	Clinicians (dentists) with at least 2 years of clinical experience, selected for good agreement with consensus	no	no	reported as reading the index image only		yes_interobserver_statistic	other	no	κ = 0.590 (Fleiss' kappa, dental students without AI support, from a cited study, not the present study's own annotators)	A recent study investigated the diagnostic accuracy and consistency (Fleiss' κ) of dental students for caries detection in bitewings with and without support from an AI system.Without support, the dental students achieved a sensitivity, specificity, F1-score, and κ of 0.679, 0.877, 0.664, and 0.590 respectively, showing an accuracy similar to the 3 dentists ().
S16	Öksüzoğlu	2	Research assistant with 1 year experience in Oral and Maxillofacial Radiology and 3 years in Endodontics; senior endodontist with 28 years clinical	yes	no	3D access is the index volume itself; no information beyond the index image reported		only_qualitative_consensus_statement	ground_truth_annotators			Third, we did not report intra- or inter-examiner agreement metrics, as the reference standard was established via expert supervision and consensus review rather than independent repeated annotations.

row_id	first_author	n_annotators	annotator_expertise	annotator_had_3d_or_clinical_access	annotator_access_beyond_index_image	access_beyond_basis	annotator_access_quote	iaa_statistic_reported	iaa_pertains_to	iaa_pertains_to_own_labels	iaa_value	iaa_quote
			experience									
S17	Allihaibi		experienced endodontists/clinicians	no	no	reported as reading the index image only		yes_interobserver_statistic	ground_truth_annotators	yes	Intra-examiner kappa: 0.90 (CBCT), 0.81 (PA); Inter-examiner kappa: 0.71 (CBCT), 0.73 (PA)	Intra-examiner Kappa scores were 0.90 for CBCT and 0.81 for PA, while inter-examiner agreement was 0.71 for CBCT and 0.73 for PA.
S18	Arı	2	a dentomaxillofacial radiologist (T.A.); reviewed and, if necessary, corrected by a researcher with over 10 years of experience in both oral and maxil	no	no	reported as reading the index image only	CBCT axial slice images were labeled using CranioCatch labeling software (Eskişehir, Türkiye).	no				

row_id	first_author	n_annotators	annotator_expertise	annotator_had_3d_or_clinical_access	annotator_access_beyond_index_image	access_beyond_basis	annotator_access_quote	iaa_statistic_reported	iaa_pertains_to	iaa_pertains_to_own_labels	iaa_value	iaa_quote
			lofacial radiology and artificial intelligence applications in dentistry (I.S.B.)									
S19	Bayrakdar		two experts (EB and ISB) with at least 10 years of experience in oral and maxillofacial radiology	no	no	reported as reading the index image only	PRs were manually segmented using CranioCatch labeling software (Eskisehir, Turkey) by dental practitioners of varying experience levels for the various dental conditions and the anatomical landmarks shown in Table 1.	no				
S20	Bennasar							no				
S21	Gosavi	2	Two senior endodontists (consensus diagnosis), plus specialist evaluators described as endodontic specialists	yes	yes	reported information beyond the index image: clinical finding, percussion, palpation, vitality, sinus tract		yes_interobserver_statistic	ground_truth_annotators	yes	Fleiss' κ=0.742	Inter-specialist agreement demonstrated substantial concordance (Fleiss' κ=0.742), indicating good consistency among experts.
S	Ha			yes	yes	report		no				

row_id	first_author	n_annotators	annotator_expertise	annotator_had_3d_or_clinical_access	annotator_access_beyond_index_image	access_beyond_basis	annotator_access_quote	iaa_statistic_reported	iaa_pertains_to	iaa_pertains_to_own_labels	iaa_value	iaa_quote
22	mdan					ed information beyond the index image: radiology report						
S23	Hamdan	3	1 of 3 oral and maxillofacial radiologists with board certification and a minimum of 10 years of experience	yes	yes	reported information beyond the index image: radiology report	If a radiology report indicated the presence of an apical radiolucency, the primary investigator re-examined the corresponding CBCT volume for further analysis.	no				
S24	Ibraheem	3	Oral radiologists (two labeling experts plus a third reconfirming radiologist)					yes_interobserver_statistic	ground_truth_annotators	yes	0.95 (Cohen's kappa)	The Cohen test of inter-observer reliability revealed a score of 0.95 between the two oral radiologists.
S25	Kim							no				
S26	Liu	3	oral radiologists with over 15 years of experience					only_qualitative_consensus_statement	ground_truth_annotators			To ensure the reliability of the standards, the presence of PLs in the images was confirmed only if at least two out of the three experts reached a consensus on the annotation results.
S27	Pekiner	5	a dental student who	no	no	reported as reading the	A dental student (MYP), who had previously undergone segmentation training to identify periapical lesions, along with two of the authors (HY and AGOT), labelled the patient images using the CranioCatch programme (CranioCatch, Eskisehir, Turkey).	no				

row_id	first_author	n_annotators	annotator_expertise	annotator_had_3d_or_clinical_access	annotator_access_beyond_index_image	access_beyond_basis	annotator_access_quote	iaa_statistic_reported	iaa_pertains_to	iaa_pertains_to_own_labels	iaa_value	iaa_quote
			had previously undergone segmentation training, along with two of the authors, labelled the patient images; two oral and maxillofacial radiologists examined the labelled periapical lesions			index image only						
S28	Saber	3	Two expert examiners (dental radiographic experts) annotated independently, third expert consulted for consensus on disagreeme	no	no	reported as reading the index image only		yes_interobserver_statistic	ground_truth_annotators	yes	kappa values 0.97 (intra-observer) and 0.96 (inter-observer)	The radiographs were re-scored at a1-month interval to assess the intra- and inter-observer agreement, which was almost perfect (kappa values 0.97 and 0.96, respectively).

row_id	first_author	n_annotators	annotator_expertise	annotator_had_3d_or_clinical_access	annotator_access_beyond_index_image	access_beyond_basis	annotator_access_quote	iaa_statistic_reported	iaa_pertains_to	iaa_pertains_to_own_labels	iaa_value	iaa_quote
			nts									
S29	Szabó	2	Two dentomaxillofacial radiologists (>10 years and >30 years experience); calibration training also involved a fifth-year dental student, a general dentist, and a dentomaxillofacial radiologist with >10 years experience, supervised by a senior radiologist with >30 years experience	no	no	reported as reading the index image only		only_qualitative_consensus_statement	ground_truth_annotators			In instances where there was disagreement regarding the detection of PL on the selected PRs, a consensus was achieved in every case.
S30	Chau	2	an expert maxillofacial radiologist (AA) and a trainee	yes	no	3D access is the index volume itself; no informatio	Periapical lesions in the CBCT scans were subsequently manually labelled in red by CKK and AA with ITK-SNAP [citation omitted].	no				

row_id	first_author	n_annotators	annotator_expertise	annotator_had_3d_or_clinical_access	annotator_access_beyond_index_image	access_beyond_basis	annotator_access_quote	iaa_statistic_reported	iaa_pertains_to	iaa_pertains_to_own_labels	iaa_value	iaa_quote
			d operator (CKK)			n beyond the index image reported						
S31	Chen		Clinician experts in CBCT segmentation; reviewers calibrated and trained in CBCT analysis and segmentation	yes	no	3D access is the index volume itself; no information beyond the index image reported		only_qualitative_consensus_statement	ground_truth_annotators			who supervised the segmentation process and resolved any disagreements by joint discussion.
S32	Hirata	2	two experts who are an oral and maxillofacial radiologist and an endodontist, both with 10 years of experience	no	no	reported as reading the index image only	Images were manually annotated with bounding boxes covering PRAs by a consensus of two experts who are an oral and maxillofacial radiologist and an endodontist, both with 10 years of experience.	no				
S33	Liu	3	oral radiologists with more than 15 years of exper	no	no	reported as reading the index image only		only_qualitative_consensus_statement	ground_truth_annotators			Although the diagnosis of periapical lesions required the agreement of at least two experts, this could still introduce a degree of subjectivity.

row_id	first_author	n_annotators	annotator_expertise	annotator_had_3d_or_clinical_access	annotator_access_beyond_index_image	access_beyond_basis	annotator_access_quote	iaa_statistic_reported	iaa_pertains_to	iaa_pertains_to_own_labels	iaa_value	iaa_quote
			ience each									
S34	Rampf	2	two dental experts with decades of clinical experience	no	no	reported as reading the index image only	All images were assessed in dimly lit rooms on diagnostic screens (21.3-inch, EIZO RadiForce R22, EIZO, Rülzheim, Germany).	yes_interobserver_statistic	inter-rater reliability of the two experts establishing the reference test	yes	.92 for bitewing images, .94 for periapical images	The inter-rater reliability of the experts was excellent (κ= .92 for bitewing images, κ= .94 for periapical images).
S35	Viet	3	3 experienced dentists	no	no	reported as reading the index image only	9728 PR were assessed to detect and classify periapical lesions based on the PAI scoring system by 3 experienced dentists.	yes_interobserver_statistic	inter-examiner agreement/calibration among the three dentists	yes	0.864	Subsequently, they were recalibrated using 40 additional PR, resulting in a Kappa index of 0.864.
S36	van Nistelrooij							no				Although frequently used for assessing tooth impaction and identifying cysts, tumors, and other bony or osteolytic pathologies, the diagnostic accuracy of PRs is limited by interobserver variability and the 2D representation of complex 3D maxillofacial structures [–].
S37	Fu	2	2 well-trained endodontists	yes	yes	reported information beyond the index image: patholog, health record	The gold standard diagnoses of AP were verified by 2 endodontists (W.T.F. and N.L.) using pathology and/or CBCT reports.	no				
S38	Hadzic	3	two senior oral surgeons with >15 years of experience and one junior dentis	yes	no	3D access is the index volume itself; no information beyond the		no				

row_id	first_author	n_annotators	annotator_expertise	annotator_had_3d_or_clinical_access	annotator_access_beyond_index_image	access_beyond_basis	annotator_access_quote	iaa_statistic_reported	iaa_pertains_to	iaa_pertains_to_own_labels	iaa_value	iaa_quote
			t			index image reported						
S39	Huang	3	Two senior graduate students in endodontic honors program, supervised by an endodontist	yes	no	3D access is the index volume itself; no information beyond the index image reported		only_qualitative_consensus_statement	ground_truth_annotators			Disagreements were resolved by joint discussion and included the endodontist and an oral radiologist (MM).
S40	Lee	2	2 independent, previously calibrated endodontists	no	no	reported as reading the index image only	Presence of a follow-up periapical radiograph more than 3 years after root filling and the availability of the periapical index (PAI)24 of the targeted tooth.	yes_interobserver_statistic	PAI index evaluation by two endodontists	yes	0.645	The PAI index was evaluated by 2 independent, previously calibrated endodontists (kappa 5 0.645), and the score was determined as follows:
S41	Ari	2	research assistant with 2 years of experience and a dento-maxillofacial radiologist with 12 years of experience	no	no	reported as reading the index image only		no				
S42	Kirnbauer	1	an oral surgeon specializin	no	no	reported as reading the index		no				

row_id	first_author	n_annotators	annotator_expertise	annotator_had_3d_or_clinical_access	annotator_access_beyond_index_image	access_beyond_basis	annotator_access_quote	iaa_statistic_reported	iaa_pertains_to	iaa_pertains_to_own_labels	iaa_value	iaa_quote
			g in oral radiology			image only						
S43	Qian	4	4名主治医生	yes	no	3D access is the index volume itself; no information beyond the index image reported	从49例CBCT原始数据中随机抽取6个根尖病变，慢性根尖周炎的CBCT数据以DICOM格式导出，图像由4名主治医生先后2次（间隔2周）记录颌骨病变的位置并对于慢性根尖周炎病变区域进行识别同时使用Materialize Mimics Medical软件进行手动逐层分割。	yes_interobserver_statistic	inter-observer and intra-observer agreement of manual segmentation	yes	0.994	测量者之间一致性的ICC值为0.994（95%CI：0.986~0.998），测量者1的自身重复测量的ICC值为0.999（95%CI：0.990~1.000），测量者2的重复测量ICC值为0.995（95%CI：0.968~0.999），测量者3的重复测量ICC值为1（95%CI：1.000~1.000），测量者4的重复测量ICC值为0.999（95%CI：0.995~1.000）。
S44	Moidu	3	Three endodontists	no	no	reported as reading the index image only	The endodontists separately viewed the IOPAR in a dimly lit room on a 22-inch monitor (1680×1050 pixels; Dell, TX, USA).	yes_interobserver_statistic	intra- and inter-observer reliability of the three endodontists	yes	0.76 (0.75–0.78) and 0.71 (0.70–0.73)	The intra- and inter-observer reliability of the three endodontists was determined to be 0.76 (0.75–0.78) and 0.71 (0.70–0.73), respectively (average Cohen kappa score).
S45	Orhan	1	an oral and maxillofacial radiologist with 8 years of professional experience	yes	no	3D access is the index volume itself; no information beyond the index image reported	Jaw location and lesion-associated teeth were recorded, and lesion volume was calculated by manual segmentation methods using Fujifilm-Synapse 3D software (Fujifilm Medical Systems, Tokyo, Japan).	yes_interobserver_statistic	intraobserver compliance for repeated volume measurements	yes	0.998	The intraclass correlation coefficient (95% confidence interval) was 0.998 (0.996–0.999) for these measurements.
S46	Setzer	3	an oral and maxillofacial radiologist (M.M	yes	no	3D access is the index volume itself; no	All CBCT volumes were initially reviewed on 24-inch light-emitting diode monitors (resolution 1920 ! 1200 at 60 Hz) using the proprietary software provided by the CBCT company (iDixel, Morita Mfg).	yes_interobserver_statistic	agreement between the three reviewers on lesion	yes	93.1%	The initial agreement between the reviewers was 27 of 29 lesions (93.1%).

row_id	first_author	n_annotators	annotator_expertise	annotator_had_3d_or_clinical_access	annotator_access_beyond_index_image	access_beyond_basis	annotator_access_quote	iaa_statistic_reported	iaa_pertains_to	iaa_pertains_to_own_labels	iaa_value	iaa_quote
			.), an endodontist (F.C.S.), and a senior graduate student completing a radiology honors program (K.J.S.)			information beyond the index image reported			presence			

S1e. Validation design and dataset size

row_id	first_author	external_validation_type	external_validation_final	external_validation_abstract_flag	external_validation_quote	dataset_size_upgraded	dataset_size_unit	dataset_size_abstract_level	dataset_size_quote
S01	Adıgüzel	internal_split	internal_split	False	First, the dataset was derived from a single academic center, and the reference standard was established by consensus interpretation of two experienced clinicians based on twodimensional periapical radiographs.	120	radiographs	120	This retrospective study was conducted on 120 digital periapical radiographs following approval by the institutional ethics committee (Approval No: 25/02/2026-22).
S02	Akyüz	internal_split	internal_split	False	Furthermore, the absence of an independent external validation dataset restricts the extrapolation of these findings to broader clinical populations.	60	patients	60	This retrospective diagnostic accuracy study was conducted on periapical radiographs from 60 patients undergoing endodontic treatment of mandibular molars.
S03	Almutairi	internal_split	internal_split	False	Periapical radiographs were retrospectively obtained from a private dental clinic archive.	271	radiographs	271	A total of 271 anonymized periapical radiographs met the inclusion criteria and were analyzed.
S04	Bubna	internal_split	internal_split	False	Digital periapical radiographs were retrieved from the database of a dental imaging service located in southern Brazil.	75	radiographs	75	Consequently, 75 periapical radiographs of anterior teeth from either the maxilla or the mandible were selected according to the eligibility criteria, evenly distributed between cases with and without periapical lesions.
S05	Erkal	internal_split	internal_split	False	Expert reference masks were created in ImageJ, and the dataset was divided at the patient level into training, validation, and independent test sets.	900	radiographs	900	The final dataset therefore comprised 900 anonymized periapical radiographs, which were subsequently divided at the patient level into training (= 594), validation (= 145), and independent test (= 161) sets to prevent information leakage.
S06	Hassanein	multicentre	multicentre	False	The images were sourced from four independent private dental clinics, each contributing 64 anonymized periapical radiographs.	256	radiographs	270	After excluding 14 images with incomplete visualization or overlapping anatomy, 256 radiographs remained for analysis ().
S07	Kunnath Menon	none_stated	none_stated	False	Statistical Analysis The diagnostic sensitivity, specificity, positive predictive value (PPV), negative predictive value (NPV), receiver operating characteristic curve (ROC), and area under the ROC of the tested dataset were calculated using Stata version 15.0 (StataCorp) for statistical analysis [].	300	radiographs	400	To account for the potential coexistence of the target pathologies in a single radiograph and to enhance the statistical power of this study, a total of 300 radiographs were analyzed.
S08	Lee	internal_split	internal_split	False	This retrospective study utilized clinical and radiographic records from Yonsei University Dental Hospital, from patients treated between 2008 and 2015.	5991	radiographs	5991	As shown in the STARD flowchart (Figure 1), a total of 5991 cases were included in this study.
S09	Marwaha	internal_split	internal_split	False	The dataset was then randomly divided into training (80%, n=998), validation (10%, n=124) and testing (10%, n=125) sets and stratified to ensure that classes were balanced.	1247	radiographs	1247	A total of 1,247 periapical radiographs meeting inclusion criteria were analyzed.
S10	Mayer	internal_split	internal_split	False	A total of 255 FMX acquired between January 2014 and September 2024 were screened from the institutional database.	200	radiographs	200	The first group consisted of 100 medium-resolution full-mouth series (FMX) including

row_id	first_author	external_validation_type	external_validation_final	external_validation_abstract_flag	external_validation_quote	dataset_size_upgraded	dataset_size_unit	dataset_size_abstract_level	dataset_size_quote
									periapical and bitewing radiographs (96-300 dpi), and the second group consisted of 100 high-resolution FMX including periapical and bitewing radiographs (≥720 dpi).
S11	Obuchowicz	internal_split	internal_split	False	In each outer cross-validation fold, approximately 80% of RVG–CBCT pairs were used for model training and hyperparameter tuning (inner loop), while the remaining 20% were held out for independent testing.	56	radiographs	109	The final dataset comprised 56 RVG-CBCT image pairs representing various tooth types, including incisors, premolars, and molars.
S12	Sadr	internal_split	internal_split	False	We exclusively utilized the training dataset of 705 images for both training and validating our models.	705	images	705	The training set comprises 705 annotated panoramic X-ray images, while the validation set contains 50 images categorized according to specific dental diseases.
S13	Scarano	internal_split	internal_split	False	We validated these parameters (,,,) through extensive ablation studies on a held-out validation set, optimizing the trade-off between computational efficiency and diagnostic accuracy; in fact we correctly associated 90.32% of the lesions with their respective teeth using these settings.	3926	radiographs		The publicly released periapical lesion detection dataset [] consists of 3926 images with bounding-box annotations established by three experienced dentists (each with more than 5 years of clinical practice), classifying periapical lesions based on the Periapical Index (PAI) scoring system.
S14	Shahmaleki	internal_split	internal_split	False	To ensure a systematic and unbiased evaluation of the YOLOv7 model, the initial dataset of 333 panoramic radiographs was divided into three subsets: a training set, a validation set and an independent test set.	333	radiographs	333	Following a meticulous review, 333 radiographs with 391 periapical lesions were selectively selected in accordance with predefined inclusion criteria (Figure 1).
S15	van Nistelrooij	internal_split	internal_split	False	More specifically, a selection of 242 held-out patients with 200 OPGs, 150 bitewings, and 50 PAs was determined with stratification based on image modality, age group, gender, ethnicity, FDI numbers, and dental findings using Scikit-Multilearn (v.	3705	radiographs	3705	Data 3705 dental radiographs from 2094 patients were retrospectively collected from Andel Elite Dental Center, Hlohovec, Slovakia and La Clinique, Alexandria, Egypt, between 2023 and 2024 using convenience sampling.
S16	Öksüzoğlu	internal_split	internal_split	False	According to the nnU-Net dataset configuration, the data were divided into three subsets: 80% for training (= 112), 10% for validation (= 14) and 10% for testing (= 14).	140	CBCT_scans	140	Ten scans were excluded because of poor image quality, leaving 140 CBCT scans for the final dataset.
S17	Allihaibi	none_stated	none_stated	False	Due to the retrospective design of this analysis, an a priori sample size calculation was not conducted.	334	radiographs	339	The study analysed the periapical status of 339 teeth from 334 preoperative periapical radiographs and their corresponding CBCT images from four prospective clinical outcome studies (Knight et al.,; Patel et al.,; Rahim,; Zahran et al.,) conducted at Guy's and St Thomas' NHS Foundation Trust (London, UK).
S18	Arı	internal_split	internal_split	False	Since the CBCT volumes were processed as individual slices in JPEG format, to prevent data leakage and minimize correlation-related bias, the test sets included only images from patients who were not represented in the training or validation sets.	300	images	300	The data set was composed of 300 CBCT images selected from the radiography archive of Eskişehir Osmangazi University Faculty of Dentistry, Department of Oral and Maxillofacial Radiology.
S19	Bayrakdar	internal_split	internal_split	False	The dataset was divided into three distinct sets: 80% for training, 10% for validation, and 10% for testing.				
S20	Bennasar	internal_split	internal_split	False	The use of LOOCV is particularly valuable for assessing the performance of artificial intelligence models, as it systematically excludes one data point from the training set, using it as a validation or test instance.	108	radiographs		All periapical radiographs, 108 in total since due to geometric distortion or anatomical noise 11 were excluded, used for DL model training were preoperative images obtained prior to NSRCT, labeled based on post-treatment follow-up outcomes (healed: 0/not healed: 1) after a minimum of 9 years (and).
S21	Gosavi	internal_split	internal_split	False	The test set was completely independent from AI training and validation data.	500	radiographs	500	A total of 500 periapical radiographs of permanent teeth were selected based on predefined criteria.
S22	Hamdan	internal_split	internal_split	False	The dataset was automatically split into training (70%), development (20%), and testing (10%) subsets using the platform's "Auto Split" feature, minimizing selection bias.	312	radiographs	312	A final dataset of 312 periapical radiographs was curated, comprising 181 images with at least one confirmed apical radiolucency and 131 lesion-free images used as controls.
S23	Hamdan	internal_split	internal_split	False	It's important to note that this training subset was derived from a distinct group of patients and did not overlap with the patients included in the testing or validation collections, ensuring that the model did not overfit to the training data.	68	radiographs	68	The final testing subset was created by randomly selecting 68 radiographs from the positive (n ¼ 130) and control (n ¼ 132) sets.
S24	Ibraheem	none_stated	none_stated	False	A total of 300 digital IOPARs, representing 1030 teeth, were selected.	300	radiographs	300	A total of 300 digital IOPARs, representing 1030 teeth, were selected.
S25	Kim	internal_split	internal_split	False	The 3D intermediate panorama dataset, consisting of 312 CBCT samples, was divided into 250 samples for training, 31 for validation, and 31 for testing.	312	patients	312	CBCT scans from 3 420 patients, taken between 2016 and 2018 for the diagnosis of impacted third molars and temporomandibular disorders, were analysed, with a total of 312 (mean age: 40.2 ± 18.1 years) selected after applying the following exclusion criteria (Table 1).

row_id	first_author	external_validation_type	external_validation_final	external_validation_abstract_flag	external_validation_quote	dataset_size_upgraded	dataset_size_unit	dataset_size_abstract_level	dataset_size_quote
S26	Liu	internal_split	internal_split	False	These images were divided into a training set and a validation set in an 8:2 ratio.	1305	radiographs	800	After manually segmenting individual teeth, a total of 1,305 PRs were collected, including 650 positive for PLs and 655 negative.
S27	Pekiner	internal_split	internal_split	False	The dataset was partitioned into three subsets to avoid reusing training images during evaluation: 80% for training, 10% for validation, and 10% for testing.	1500	patients	1500	The inclusion criteria of the study involved orthopantomography (OPTG) images of 1,500 patients aged ≤18 years or older, each with at least 8 existing teeth and with periapical lesions in at least one of these teeth.
S28	Saber	external_same_country	external_same_country	False	The models were also externally validated against 70 new images acquired from a new source (private dental clinic) acquired using Planmeca Proscanner 2.0 (Planmeca Oy, Helsinki, Finland) and fulfilling the same inclusion criteria as the initial dataset to assess their generalizability.	699	radiographs	699	A total of 699 digital periapical radiographs were retrospectively collected from the university clinic and two private clinics specialized in endodontics.
S29	Szabó	none_stated	none_stated	False	Regarding the PRs, 308 teeth with visible periapical radiolucency and 308 teeth without any periapical radiolucency (PARL) were selected.	357	radiographs	616	Results From the 357 PRs, 308 teeth with clearly visible PARL and 308 teeth without PARL were selected.
S30	Chau	internal_split	internal_split	False	This study used a 5-fold cross-validation approach to estimate the model prediction performance.	185	scans	185	Eventually, our dataset had 185 CBCT scans in total (n = 185).
S31	Chen	internal_split	internal_split	False	The CBCT dataset was divided into a training, validation, and testing set, comprising 103, 10, and 25 images, respectively.	138	CBCT_scans	138	A total of 138 de-identified CBCT images from the Department of Endodontics, Penn Dental Medicine, University of Pennsylvania were used for this investigation.
S32	Hirata	internal_split	internal_split	False	A hold-out validation method was used.	2266	roots	2266	A total of 2266 periapical root areas (PRAs) from 520 periapical radiographs (Pas) were selected and scored by experts.
S33	Liu	internal_split	internal_split	False	The dataset was split 8:2 for training and validation.	1305	radiographs	1305	A total of 1,305 periapical radiographs were collected, comprising 650 positive and 655 negative cases.
S34	Rampf	internal_split	internal_split	False	Radiographs utilized in this study were randomly selected from the pre-existing imaging resources available at the Heidelberg University.	16	images	16	During an 8-week self-assessment period, both groups of students were granted unrestricted access to 16 virtual worked example cases featuring 8 bitewing images (for caries detection) and 8 periapical radiographs (for detection of apical periodontitis).
S35	Viet	internal_split	internal_split	False	2658 PR then was randomly allocated in ratios of 80%, 10% and 10% for training, validation and testing.	2658	radiographs	2658	Finally, 2658 PR were selected for the study (Table 1).
S36	van Nistelrooij	internal_split	internal_split	False	Five-fold cross-validation splits were determined for the train/validation PRs from OdontoAI and DENTEX datasets using multi-label stratification [].	1661	radiographs		The remaining 1,661 PRs were split into 1,337 PRs for training and validation and 324 PRs for testing using multi-label stratification based on the FDI numbers [].
S37	Fu	external_independent_dataset	external_independent_dataset	False	The first data set was obtained from Xiangyang Stomatological Hospital (XSH) in Central China between 2019 and 2022, while the other 2 were collected from Tianjin Stomatological Hospital (TSH) in North China and Zhejiang University Stomatological School (ZUSS) in East China between 2019 and 2022.	4951	teeth	4951	Hence, the total sample size for the study is 4,951 teeth obtained from 279 patients.
S38	Hadzic	external_same_country	external_same_country	True	The software used in this study utilizes one of the four pre-trained models from [] to evaluate the lesion detection performance of the model on a new, independent dataset consisting of 195 CBCT images.	195	CBCT_scans	195	One patient image had to be excluded due to the software failing to read the file, leaving 195 patient images () for the comparison of software-based PAL detections with expert ground truth within this study.
S39	Huang	internal_split	internal_split	False	The finalized model was then assessed on the 20-sample test set.	20	CBCT_scans	20	CBCT volumes of 20 patients, de-identified and of known sex and age, were selected based on the following inclusion criteria: limited field of view with several roots and at least one periapical lesion per volume, teeth with or without definitive coronal restoration, with or without a history of endodontic treatment, and no to moderate presence of artifacts.
S40	Lee	internal_split	internal_split	False	However, since the dataset was limited to single-rooted premolars examined at a single institution, further validation should be performed using datasets obtained from other organizations or with additional tooth types.	598	teeth	598	The total number of data points in the input dataset was 598.
S41	Ari	internal_split	internal_split	False	Approximately 80% of the data set are divided into 3 parts: 80% training, 10% testing, and 10% validation.	1169	radiographs	1169	The dataset includes 1169 periapical radiographs taken from adults between January 2016 and June 2020.
S42	Kirnbauer	internal_split	internal_split	False	Both networks were trained and tested using the 4-fold cross-validation technique.	144	scans	144	A total of 144 CBCT volumes were selected, comprising 54 scans of only the lower jaw, 74 of only the upper jaw, and 16 of both jaws, which resulted in a total of 2128 teeth (206 with the presence of PALs and 1922 without the presence of PALs) that were used for training

row_id	first_author	external_validation_type	external_validation_final	external_validation_abstract_flag	external_validation_quote	dataset_size_upgraded	dataset_size_unit	dataset_size_abstract_level	dataset_size_quote
									and testing procedures.
S43	Qian	internal_split	internal_split	False	按照80%、10%、10%将数据分为训练集、验证集和测试集，49例患者CBCT数据作为训练集，测试集和验证集各5例CBCT数据。	55	teeth	55	收集北京大学口腔医院第二门诊部2017年1月—2021年12月罹患单根牙慢性根尖周炎的CBCT影像，总计49例患者55个样本。
S44	Moidu	internal_split	internal_split	False	Further, the data was limited as it was retrieved from a single center.	3540	roots	3540	A total of 3540 periapical root areas (PRA) on 2200 digital IOPAR were obtained from subjects (n=2200;52% males, 48% females; mean±SD age: 31.5±10.68 year; range: 18 to 68 year) who had undergone radiological investigation for diagnostic/treatment requirement.
S45	Orhan	external_independent_dataset	external_independent_dataset	False	CBCT scans for test data sets taken for various diagnostic purposes were obtained from the CBCT archive of Eskisehir Osmangazi University Faculty of Dentistry.	153	lesions	153	A total of 153 periapical lesion images obtained from 109 patients were included in this study.
S46	Setzer	internal_split	internal_split	False	Repeated splits of all images into a training set and a validation set based on 5-fold cross validation were performed using Deep Learning segmentation (DLS), and the results were averaged.	20	patients	20	Existing deidentified CBCT volumes of 20 patients with known age and sex were selected based on criteria allowing for training and cross validation of the DL algorithm to detect PLs.

S1f. Case-mix moderators and platform overlap

row_id	first_author	lesion_size_moderator_analysed	lesion_size_moderator_quote	region_moderator_analysed	region_moderator_quote	labelling_platform_cluster
S01	Adıgüzel	yes	When AP-positive cases were stratified according to lesion severity using the PAI system as PAI = 3 (small/moderate; n = 27) and PAI ≥ 4 (well-defined; n = 33), both Claude 4.5 Sonnet and Gemini 3.0 Pro demonstrated high sensitivity in each subgroup (PAI = 3: 92.6%; PAI ≥ 4: 97.0%).			
S02	Akyüz	no				
S03	Almutairi	no				
S04	Bubna	no				
S05	Erkal	no		no		
S06	Hassanein	no		yes	Importantly, tooth involvement was a major predictor: lesions around first molars were over 30 times more likely to be correctly classified (OR = 32.55,< 0.001), reflecting clearer radiographic landmarks and fewer superimpositions in posterior regions ().	
S07	Kunnath Menon	no		yes	For the panoramic radiographs, sensitivity, specificity, PPV, and NPV results were generated for specific sections, including overall, maxillary anterior, maxillary posterior, mandibular anterior, and mandibular posterior regions, for all the following outcomes: BL, C, F, I, IT, P, PC, PR, RF, and RR.	
S08	Lee	no				
S09	Marwaha	yes	For lesions <3 mm, AI accuracy was 87.8% compared to 64.2% for clinicians (p<0.001).			
S10	Mayer	no		no		
S11	Obuchowicz	no		no		

row_id	first_author	lesion_size_moderator_analysed	lesion_size_moderator_quote	region_moderator_analysed	region_moderator_quote	labelling_platform_cluster
S12	Sadr	no				
S13	Scarano	no		no		
S14	Shahmaleki	no				
S15	van Nistelrooij	no		no		
S16	Öksüzoğlu	yes	When stratified by lesion size (small, medium and large), lesion-wise performance differed across groups (Table).	no		
S17	Allihaibi	no		yes	TABLE 1 Diagnostic performance of clinicians and diagnocat in detecting periapical radiolucencies: tooth-level analysis of sensitivity, specificity and accuracy by dental arch and tooth type.	
S18	Arı	no				CranioCatch annotation platform; shares authors I.S. Bayrakdar, Ö. Çelik, E. Bilgir, A. Kuran, K. Orhan with S19
S19	Bayrakdar	no				CranioCatch annotation platform; shares authors with S18, S27, S41, S45
S20	Bennasar	no		no		
S21	Gosavi	yes	Small lesions (2-5mm) were detected with 76.6% sensitivity by AI and 78.2% by specialists, while large lesions (>10mm) achieved >98% sensitivity for both.	yes	Regarding tooth type, anterior teeth showed slightly higher detection rates (AI: 91.4%, Specialists: 93.8%) compared to premolars (AI: 88.1%, Specialists: 90.3%) and molars (AI: 87.9%, Specialists: 89.7%), though differences were not statistically significant.	
S22	Hamdan	no		yes	The highest sensitivity was observed in the anterior maxilla (97.7%), followed by the posterior maxilla (90.3%), posterior mandible (86.3%), and anterior mandible (76.0%).	
S23	Hamdan	no				
S24	Ibraheem	no		no		
S25	Kim	no				
S26	Liu	no		no		
S27	Pekiner	no				CranioCatch annotation platform; shares author I.S. Bayrakdar with S18, S19, S45
S28	Saber	no		no		
S29	Szabó	no		yes	In line with this result, the sensitivity and diagnostic accuracy of DC for detecting PARL on canines showed lower values at 0.27 and 0.64, respectively ().	
S30	Chau	no				
S31	Chen	no		no		
S32	Hirata	no				
S33	Liu	no		no		
S34	Rampf	no				
S35	Viet	no				
S36	van Nistelrooij	no		no		

row_id	first_author	lesion_size_moderator_analysed	lesion_size_moderator_quote	region_moderator_analysed	region_moderator_quote	labelling_platform_cluster
S37	Fu	yes	In this section, we classified lesions into 8 size-based categories, and Table 3 summarizes			
S38	Hadzic	yes	In, we plot a histogram of true positives and false negatives per lesion type, illustrating that for the smallest lesion type (class 1, with a diameter of periapical radiolucency between 0.5 and 1 mm), the sensitivity is low, while for classes 2 through 5 (diameters of periapical radiolucency larger than 1 mm, see also), the sensitivities are much higher.	yes	Sensitivity and specificity with their corresponding exact 95% confidence intervals (CIs) are presented for all teeth and stratified by upper/lower jaw and tooth type.	
S39	Huang	no		no		
S40	Lee	no				
S41	Ari	no		no		CranioCatch annotation platform; shares first author (Arı) with S18
S42	Kirnbauer	yes	By observing the predictions for individual lesions, we noticed that the mis-segmentation was primarily related to PALs with a very small or large volume.			
S43	Qian	no				
S44	Moidu	no				
S45	Orhan	no				Diagnocat platform (not CranioCatch); shares authors K. Orhan and I.S. Bayrakdar with S18, S19
S46	Setzer	no				
