# Supplementary material for: Reference Standards in Artificial Intelligence Detection of Apical Periodontitis: A Narrative Review of What Reported Accuracy Measures
Source: Diagnostics (Basel). 2026 Oct 6;16(19):3225. doi: 10.3390/diagnostics16193225 (PMC13664220; doi:10.3390/diagnostics16193225)
Supplement: Supplementary file 1 [file diagnostics-16-03225-s001.zip › 03_Table_S2_Data_Dictionary.pdf]

Table S2. Field-level data dictionary for Table S1.

Provenance level records where each field's value comes from. Because all 46 rows are now coded from published Methods sections, no field carries an abstract-level value; three fields are marked Superseded and retained only so that the upgrade from the earlier abstract-level coding remains auditable. An empty cell in Table S1 means the published article does not report that field.

Field	Provenance level	Definition	Non-empty rows (of 46)
row_id	Bibliographic	Study identifier, S01–S46, ordered by year descending then first author.	46
first_author	Bibliographic	First author surname as printed.	46
year	Bibliographic	Publication year.	46
journal	Bibliographic	Journal name.	46
title	Bibliographic	Article title.	46
doi	Bibliographic	Digital object identifier; used to match the supplied full-text PDF to the row.	46
url	Bibliographic	Resolvable article link.	46
pmid	Bibliographic	PubMed identifier where one exists.	46
pmcid	Bibliographic	PubMed Central identifier where one exists.	46
input_modality	Methods-coded	Image the model consumed: IOPA, PANORAMIC, CBCT or MIXED.	46
coding_level	Derived	Level at which the row was coded. All 46 rows are full_text.	46
access_status	Derived	Whether the full text was obtained.	46
retrieval_route	Derived	How the full text was obtained; author_supplied_pdf for the 24 studies supplied directly by the authors.	46
reference_standard_abstract_level	Superseded	Classification as it stood when the row was coded from the abstract. Retained so the upgrade is auditable; not used in any reported count.	46
reference_standard_upgraded	Methods-coded	Reference standard as described in the Methods: human_reading_same_image, CBCT_verification, histopathology, surgical_or_clinical_outcome, other_mixed, not_stated, or not_applicable_no_detection_claim.	46
same_image_or_unstated	Derived	TRUE where the reference is a reading of the index image or is not stated — the two categories in which no reference independent of the index image is established.	45
ref_quote_verbatim	Methods-coded	Verbatim sentence on which the reference-standard classification rests. Non-empty for every classified row.	46
coding_note	Derived	Adjudication note, including the author's settled decisions and any departure from a prior coding.	46
n_annotators	Methods-coded	Number of people who produced the reference labels; empty where the Methods do not state it.	46
annotator_expertise	Methods-coded	Annotator qualifications in the paper's own words.	46
annotator_had_3d_or_clinical_access	Methods-coded	Whether the label-producing readers used 3D or clinical information, as reported. For a CBCT-input study this is often yes because the 3D data are the index volume.	46
iaa_statistic_reported	Methods-coded	yes_interobserver_statistic, only_qualitative_consensus_statement, or no.	46
iaa_pertains_to	Methods-coded	What the reported agreement statistic measures, in the paper's terms.	46
iaa_value	Methods-coded	Agreement statistic as printed.	46
iaa_quote	Methods-coded	Verbatim sentence reporting the statistic.	46
external_validation_type	Methods-coded	Test-set provenance as described in the Methods: internal_split, external_same_country, external_independent_dataset, multicentre, or none_stated.	46

Field	Provenance level	Definition	Non-empty rows (of 46)
external_validation_final	Derived	Harmonised validation category used in the reported counts.	46
external_validation_quote	Methods-coded	Verbatim sentence describing the validation design.	46
external_validation_abstract_flag	Superseded	TRUE when the validation category rested on the abstract. FALSE for all rows.	46
dataset_size_upgraded	Methods-coded	Principal dataset size from the Methods, harmonised to imaging units.	46
dataset_size_unit	Methods-coded	Unit in which the size is reported.	46
dataset_size_quote	Methods-coded	Verbatim sentence stating the dataset size.	46
dataset_size_abstract_level	Superseded	Size as it stood at abstract-level coding; now equal to the Methods value.	45
lesion_size_moderator_analysed	Methods-coded	Whether performance is analysed by lesion size.	46
lesion_size_moderator_quote	Methods-coded	Verbatim sentence supporting the preceding field.	46
region_moderator_analysed	Methods-coded	Whether performance is analysed by anatomical region.	46
region_moderator_quote	Methods-coded	Verbatim sentence supporting the preceding field.	46
aff_country	Bibliographic	First-author affiliation country; empty where not identifiable from metadata.	46
included_in_denominator	Derived	TRUE for the 44 studies contributing to every reported proportion; FALSE for the 2 out-of-scope studies retained for auditability.	46
exclusion_reason	Derived	Why an excluded study is out of scope; empty for included studies.	2
exclusion_quote	Methods-coded	Verbatim sentence from the excluded study supporting its exclusion.	2
annotator_access_quote	Methods-coded	Verbatim sentence describing what the annotators had access to.	21
quote_splice_note	Derived	Flags the two rows whose quoted sentence is interrupted in the published PDF text layer by a running head or figure caption; the stored quote is the contiguous printed fragment.	2
annotator_access_beyond_index_image	Derived	Whether that information went beyond the index image. Distinguishes genuine independence from reading the index volume in three dimensions; this field, not the preceding one, supports the reported count.	40
access_beyond_basis	Derived	Basis on which the preceding field was derived.	40
iaa_pertains_to_own_labels	Derived	Whether that statistic pertains to the study's own reference labels rather than a separate reader study or a model-against-human comparison. Supports the reported agreement count.	21
labelling_platform_cluster	Derived	Shared annotation platform and overlapping authorship, where present. Records that these rows are not fully independent samples.	5
