# Supplementary material for: Reference Standards in Artificial Intelligence Detection of Apical Periodontitis: A Narrative Review of What Reported Accuracy Measures
Source: Diagnostics (Basel). 2026 Oct 6;16(19):3225. doi: 10.3390/diagnostics16193225 (PMC13664220; doi:10.3390/diagnostics16193225)
Supplement: Supplementary file 1 [file diagnostics-16-03225-s001.zip › 04_Table_S3_Search_and_coding_methods.pdf]

Table S3. Search and coding methods.

This table provides the available search terms, the recorded search dates, restrictions, deduplication, and screening and coding procedures. The PubMed queries are listed in the search log that follows (Table S3, continued) as they were logged when run, with the date, publication-date limit, sort order, maximum number of records requested, and the numbers of records matching and returned. The queries were developed iteratively and each retrieved a capped number of records, so the log documents the search as it was conducted; re-running it would not regenerate the same set of records. "Not recorded" marks a detail that was not captured; such items are listed so that the limit of reproducibility is visible rather than implied.

Item	Procedure as documented	Source	Note / not recorded
Databases and sources searched	PubMed only. Queries were run programmatically through the NCBI E-utilities interface; no other bibliographic database was searched. OpenAlex was used for citation tracing, PubMed Central, Europe PMC, Unpaywall and publisher sites for full-text retrieval and look-up of known articles, and CrossRef for DOI resolution and retraction notices (see the corresponding rows).	Search log (Table S3, continued); Authors' project record	
Search 1 — primary AI detection studies (search string)	Concept structure: (periapical lesion OR apical periodontitis OR periapical radiolucency OR periapical OR endodontic) AND (artificial intelligence OR deep learning OR convolutional neural network OR CNN OR U-Net OR segmentation OR detection), in some queries combined with imaging terms (periapical radiograph, intraoral radiograph, cone-beam computed tomography) or reference-standard terms (reference standard, ground truth); index test restricted to intraoral periapical, panoramic or CBCT imaging. Executed as 12 PubMed queries on 3 August 2026, each listed in the search log as logged when run; together they returned 150 unique records, among which the 46 studies in Table S1 were identified.	Search log (Table S3, continued); Main text: Approach › Search Strategy	Queries as logged when run.
Search 2 — discordance evidence (search string)	Concept structure: imaging and disease terms of Search 1 AND (histopathology OR histolog* OR cadaver OR gold standard OR reference standard); plus studies of interobserver and intraobserver agreement for radiographic periapical diagnosis. Executed as 153 PubMed queries between 3 August and 18 September 2026 (search log).	Search log (Table S3, continued); Main text: Approach › Search Strategy	Queries as logged when run. The number of matching records was not captured for 13 of these queries.
Search 3 — methodological literature (search string)	Concept structure: reporting and appraisal instruments (CLAIM, STARD, STARD-AI, PRIDASE, TRIPOD+AI, QUADAS-2, QUADAS-AI, SANRA) AND the statistical literature on reference standards: imperfect reference standard; missing reference standard; verification bias; incorporation bias; dependent errors and conditional dependence; latent class models. Executed as 84 PubMed queries between 3 August and 18 September 2026 (search log). A further 27 queries, listed as 'Background', supplied context statements such as disease burden and prevalence.	Search log (Table S3, continued); Main text: Approach › Search Strategy	Queries as logged when run. The number of matching records was not captured for 3 of these queries.
Final search dates	Search 1: 3 August 2026. Searches 2 and 3: first	Search log (Table S3, continued); Authors' project	Dates are those on which each query was run

Item	Procedure as documented	Source	Note / not recorded
	run 3 August 2026, final queries 18 September 2026. Citation tracing: 4 August 2026. Full-text retrieval of the 24 studies not available open access: 14–15 August 2026. The audited set comprises the studies identified among the records returned by the Search 1 queries of 3 August 2026; queries run later for Searches 2 and 3 did not add studies to the audit.	record	(UTC), as logged.
Date limits	None on 11 of the 12 Search 1 queries; one carried a 2019–2026 publication-date limit. Across all 276 queries, 177 carried no date limit; the limit applied to each of the others is shown in the search log.	Search log (Table S3, continued)	
Language restrictions	None. No query carried a language filter.	Search log (Table S3, continued)	
Publication-status restrictions	Reviews, editorials and conference abstracts excluded. Primary studies only.	Main text: Approach › Search Strategy	Preprint servers and grey-literature sources were not searched, PubMed being the only database. None of the 122 records cited or audited is indexed in PubMed as a preprint (publication type checked 23 September 2026).
Eligibility criteria	Primary study applying an AI system to intraoral periapical, panoramic or CBCT imaging, reporting detection, segmentation or grading performance for apical periodontitis separately from other findings. Excluded: other dental conditions; studies in which periapical lesions were an incidental class without separately reported performance; two apical-periodontitis studies with no detection claim (retained in Table S1 with verbatim exclusion quote).	Main text: Approach › Search Strategy	
Citation tracing	Backward (reference lists) and forward (citing articles) tracing through the OpenAlex citation index on 4 August 2026, from four anchor studies of the discordance evidence — Kanagasingam et al. 2017, doi:10.1111/iej.12650 (main-text reference [1]); de Paula-Silva et al. 2009, doi:10.1016/j.joen.2009.04.006 [11]; Hilmi et al. 2023, doi:10.1111/iej.13921 [19]; and Estrela et al. 2008, doi:10.1016/j.joen.2007.11.023 [32] — and from one methodological paper, Walter et al. 2012, doi:10.1002/sim.4444 [70]; up to 60 references and 80 citing works per anchor study, and 50 and 60 for the methodological paper. Eleven of the 86 cited references were not returned by any logged query and entered through citation tracing or known-item lookup: main-text references [5], [25], [28], [51], [65], [66], [67], [70], [71], [81] and [84].	Authors' project record (logged tracing calls)	
Deduplication procedure	Records deduplicated by DOI and PMID.	Authors' project record	
Title/abstract screening — number of people	Two authors. Screening was performed in a single pass with generative-AI assistance (Claude Opus 5, Anthropic) directed by the authors; it was not independently duplicated by a second human screener.	Authors' project record; Main text: Approach › Review Design and Adherence; Main text: Limitations	Which author made the final screening decisions is not recorded beyond the CRediT statement (investigation and data curation: O.R.A.).

Item	Procedure as documented	Source	Note / not recorded
Full-text screening — number of people	As for title/abstract screening: single pass, AI-assisted, author-directed, not independently duplicated.	Authors' project record; Main text: Approach › Review Design and Adherence	
Full-text retrieval	Six routes attempted (PubMed Central, Europe PMC full-text XML, Unpaywall, Semantic Scholar, CrossRef text-and-data-mining links, publisher DOI resolution), reaching 22 studies via PubMed Central; the remaining 24 were obtained by the authors through institutional subscription access on 14–15 August 2026. All 46 identified studies were coded at full-text level.	Main text: Approach › Coding of the Reference Standard; Table S1 (fields coding_level, access_status, retrieval_route, ref_quote_verbatim); Authors' project record	Table S1: coding_level = full_text for 46/46; access_status pmc_full_text 22, full_text_obtained 24; retrieval_route records the PMCID for each of the 22.
Coding of the 47 fields — number of people	Two authors. The 47-field coding was performed in a single pass with generative-AI assistance (Claude Opus 5, Anthropic) directed by the authors. There was no independent duplicate human coding.	Authors' project record; Main text: Approach › Coding of the Reference Standard	
Verification of reference-standard classification	Every reference-standard classification was verified by the authors against the verbatim Methods sentence recorded in Table S1 (field ref_quote_verbatim, populated for 46 of 46 studies).	Authors' project record; Main text: Approach › Coding of the Reference Standard; Table S1 (fields coding_level, access_status, retrieval_route, ref_quote_verbatim)	
Independent duplication of reference-standard classification	No. Classification was not independently performed by more than one reviewer.	Authors' project record; Main text: Approach › Review Design and Adherence	
Procedure for resolving coding disagreements	None. No formal disagreement-resolution procedure existed because coding was not duplicated.	Authors' project record	
Annotation-quality fields	Coded only from Methods text; an empty cell denotes a field the published Methods do not report.	Main text: Approach › Coding of the Reference Standard	
Dataset-size harmonisation	Imaging units (distinct radiographs for 2D; volumes or examinations for 3D), never teeth, roots, lesions, slices or augmented copies.	Main text: Approach › Coding of the Reference Standard	
Retraction check	CrossRef used for DOI resolution and retraction checks.	Authors' project record	Date of the retraction check: 19 September 2026.
Protocol registration	None.	Main text: Approach › Review Design and Adherence; Main text: Limitations	
Risk-of-bias assessment	None applied.	Main text: Limitations	
Reporting guideline	SANRA (six-item scale for narrative reviews). No PRISMA adherence claimed.	Main text: Approach › Review Design and Adherence	
Data dictionary	Table S2, field-level data dictionary for Table S1.	Main text: Approach › Coding of the Reference Standard; Main text: Supplementary Materials	

Table S3 (continued). Sensitivity analysis excluding the two prognostic studies (S20 Bennasar 2025; S40 Lee 2023).

Analysis	Quantity	n	%
principal (n=44)	denominator (included studies)	44	100.0
principal (n=44)	same-image or unascertainable reference (same_image_or_unstated)	34	77.3
principal (n=44)	explicit same-image human reading	33	75.0
principal (n=44)	reference not ascertainable	1	2.3
principal (n=44)	independent reference (any type)	8	18.2

Analysis	Quantity	n	%
principal (n=44)	independent reference appropriate for contemporaneous detection (CBCT_verification + histopathology)	6	13.6
principal (n=44)	of which CBCT_verification	5	11.4
principal (n=44)	of which histopathology	1	2.3
principal (n=44)	independent outcome used for prognosis (surgical_or_clinical_outcome)	2	4.5
principal (n=44)	mixed reference	2	4.5
principal (n=44)	CBCT-input studies	11	25.0
principal (n=44)	CBCT-input studies with independent reference	0	0.0
principal (n=44)	2D-input studies	33	75.0
principal (n=44)	2D-input studies with same-image reading	23	69.7
principal (n=44)	2D-input studies with independent reference	8	24.2
sensitivity: prognostic studies excluded (n=42)	denominator (included studies)	42	100.0
sensitivity: prognostic studies excluded (n=42)	same-image or unascertainable reference (same_image_or_unstated)	34	81.0
sensitivity: prognostic studies excluded (n=42)	explicit same-image human reading	33	78.6
sensitivity: prognostic studies excluded (n=42)	reference not ascertainable	1	2.4
sensitivity: prognostic studies excluded (n=42)	independent reference (any type)	6	14.3
sensitivity: prognostic studies excluded (n=42)	independent reference appropriate for contemporaneous detection (CBCT_verification + histopathology)	6	14.3
sensitivity: prognostic studies excluded (n=42)	of which CBCT_verification	5	11.9
sensitivity: prognostic studies excluded (n=42)	of which histopathology	1	2.4
sensitivity: prognostic studies excluded (n=42)	independent outcome used for prognosis (surgical_or_clinical_outcome)	0	0.0
sensitivity: prognostic studies excluded (n=42)	mixed reference	2	4.8
sensitivity: prognostic studies excluded (n=42)	CBCT-input studies	11	26.2
sensitivity: prognostic studies excluded (n=42)	CBCT-input studies with independent reference	0	0.0
sensitivity: prognostic studies excluded (n=42)	2D-input studies	31	73.8
sensitivity: prognostic studies excluded (n=42)	2D-input studies with same-image reading	23	74.2
sensitivity: prognostic studies excluded (n=42)	2D-input studies with independent reference	6	19.4
split of the 8 independent-reference studies (n=8)	independent reference appropriate for contemporaneous detection (CBCT_verification + histopathology)	6	75.0
split of the 8 independent-reference studies (n=8)	independent outcome used for prognosis (surgical_or_clinical_outcome: S20 Bennasar 2025; S40 Lee 2023)	2	25.0

Table S3 (continued). Search log: the PubMed queries as logged when run.

Queries are listed in the order run, as logged at the time they were run (UTC). They are assigned to Searches 1–3 by the part of the argument they served, and 'Background' queries supplied context statements such as disease burden and prevalence; the assignment was made when the log was compiled for this revision. 'Records matching' is the number of PubMed records the query matched on the date it was run; 'Records returned' is the number retrieved, capped by 'Maximum records requested'. A publication-date limit of 'none' means no date filter was applied. Look-ups of individual known articles by identifier or citation, counting queries used only to gauge the size of a literature, and queries run while scoping other review topics are not listed. Counts will differ if the queries are re-run, because PubMed grows.

No.	Search	Date run (UTC)	Query as executed	Publication-date limit	Sort order	Maximum records requested	Records matching	Records returned
1	Search 1	2026-08-03	artificial intelligence endodontics	none	default	3	676	3
2	Search 1	2026-08-03	artificial intelligence AND endodontics	none	default	1	676	1

No.	Search	Date run (UTC)	Query as executed	Publication-date limit	Sort order	Maximum records requested	Records matching	Records returned
3	Search 3	2026-08-03	artificial intelligence AND endodontics AND review[Publication Type]	none	default	1	192	1
4	Search 3	2026-08-03	artificial intelligence AND endodontics AND review[Publication Type]	2023	default	1	192	1
5	Search 1	2026-08-03	(artificial intelligence OR deep learning OR convolutional neural network) AND (periapical lesion OR apical periodontitis)	none	default	1	223	1
6	Search 3	2026-08-03	(artificial intelligence OR deep learning) AND (periapical lesion OR apical periodontitis) AND review[Publication Type]	none	default	1	62	1
7	Search 3	2026-08-03	(artificial intelligence OR deep learning) AND (periapical lesion OR apical periodontitis) AND review[Publication Type]	2023	default	1	62	1
8	Search 3	2026-08-03	(ground truth OR reference standard) AND (artificial intelligence OR deep learning) AND dentistry	none	default	1	416	1
9	Search 1	2026-08-03	(ground truth OR reference standard) AND (artificial intelligence OR deep learning) AND (endodontic OR periapical)	none	default	1	93	1
10	Search 2	2026-08-03	(periapical radiograph OR conventional radiography) AND cone beam computed tomography AND apical periodontitis AND detection	none	default	1	70	1
11	Search 2	2026-08-03	apical periodontitis AND cone beam computed tomography AND histolog*	none	default	1	38	1
12	Search 2	2026-08-03	observer variation AND periapical radiograph AND apical periodontitis	none	default	1	51	1
13	Search 3	2026-08-03	CLAIM OR STARD AND (dental OR dentistry) AND artificial intelligence reporting quality	none	default	1	22	1
14	Search 3	2026-08-03	QUADAS-2 AND dental AND artificial intelligence	none	default	1	125	1
15	Search 3	2026-08-03	reporting quality AND artificial intelligence AND dentistry	none	default	1	415	1
16	Search 3	2026-08-03	geographic AND dataset AND artificial intelligence AND medical imaging AND bias	none	default	1	3	1
17	Search 3	2026-08-03	artificial intelligence AND endodontics AND review[Publication Type]	2024/01/01–2026/12/31	default	30	155	30
18	Search 3	2026-08-03	artificial intelligence AND endodontics AND review[Publication Type]	2010–2018	default	5	1	1
19	Search 3	2026-08-03	(artificial intelligence OR deep learning OR machine learning) AND (periapical lesion OR apical periodontitis OR periapical radiolucency) AND (review[Publication Type] OR meta-analysis[Publication Type])	2022/01/01–2026/12/31	default	30	60	30
20	Search 3	2026-08-03	artificial intelligence AND endodontics AND (systematic review[Publication Type] OR meta-analysis[Publication Type])	2024/01/01–2026/12/31	default	30	24	24
21	Search 3	2026-08-03	(reference standard OR ground truth OR ground-truth) AND artificial intelligence AND (dental OR dentistry OR oral radiology)	2022/01/01–2026/12/31	default	30	434	30
22	Search 3	2026-08-03	(bias OR overestimat* OR risk of bias) AND artificial intelligence AND dental radiograph* AND review	2022/01/01–2026/12/31	default	25	94	25
23	Search 3	2026-08-03	(pitfalls OR limitations OR critical appraisal OR methodological quality) AND artificial intelligence AND dentistry	2022/01/01–2026/12/31	default	30	1623	30
24	Search 2	2026-08-03	periapical radiography AND cone beam computed tomography AND detection of apical periodontitis	none	default	30	96	30
25	Search 2	2026-08-03	cone beam computed tomography AND prevalence AND apical periodontitis AND periapical radiograph	none	default	25	26	25
26	Search 2	2026-08-03	histopathology AND radiographic AND periapical lesion AND accuracy	none	default	25	48	25
27	Search 2	2026-08-03	(cadaver OR ex vivo OR simulated lesion) AND periapical AND radiograph AND cone beam computed tomography AND detection	none	default	25	30	25
28	Search 2	2026-08-03	artificial bone lesion AND radiographic detection AND cancellous OR cortical bone AND periapical	none	default	20	240	20
29	Search 2	2026-08-03	bone lesion radiographic detection cortical bone experimental	none	default	15	5	5
30	Search 1	2026-08-03	deep learning AND periapical lesion detection AND radiograph	2019/01/01–2026/12/31	default	30	64	30
31	Search 1	2026-08-03	deep learning AND cone beam computed tomography AND periapical lesion segmentation	none	default	25	22	22
32	Search 3	2026-08-03	external validation AND deep learning AND dental radiograph	none	default	25	62	25
33	Search 3	2026-08-03	multicenter AND artificial intelligence AND dental radiograph* AND validation	none	default	20	40	20
34	Search 2	2026-08-03	interobserver agreement AND periapical radiograph AND periapical index	none	default	25	14	14
35	Search 2	2026-08-03	periapical index PAI radiographic scoring reliability	none	default	25	8	8
36	Search 3	2026-08-03	CLAIM checklist artificial intelligence medical imaging reporting	none	default	20	58	20
37	Search 3	2026-08-03	STARD-AI OR STARD 2015 diagnostic accuracy reporting artificial intelligence	none	default	20	26	20
38	Search 3	2026-08-03	QUADAS-AI risk of bias artificial intelligence diagnostic accuracy	none	default	15	29	15
39	Search 3	2026-08-03	reporting completeness OR adherence to guidelines AND artificial intelligence AND dentistry studies	2022/01/01–2026/12/31	default	25	202	25
40	Search 3	2026-08-03	geographic distribution AND artificial intelligence AND clinical trials OR datasets AND medical imaging	none	default	20	57620	20

No.	Search	Date run (UTC)	Query as executed	Publication-date limit	Sort order	Maximum records requested	Records matching	Records returned
41	Background	2026-08-03	global burden untreated caries AND pulpal AND apical periodontitis epidemiology	none	default	20	0	0
42	Search 3	2026-08-03	artificial intelligence AND oral health AND low- and middle-income countries	none	default	20	47	20
43	Search 3	2026-08-03	(circular OR circularity OR self-fulfilling) AND ground truth AND artificial intelligence AND imaging	none	default	25	15	15
44	Search 3	2026-08-03	imperfect reference standard AND diagnostic accuracy AND artificial intelligence	none	default	25	6	6
45	Search 3	2026-08-03	incorporation bias OR verification bias AND artificial intelligence diagnostic accuracy imaging	none	default	25	166	25
46	Search 3	2026-08-03	ground truth problem AND medical artificial intelligence AND label noise OR annotation quality	none	default	25	16158	25
47	Search 3	2026-08-03	annotation OR labelling variability AND deep learning AND dental radiograph	none	default	25	173	25
48	Search 3	2026-08-03	human expert labels limit AND deep learning performance ceiling AND radiology	none	default	20	0	0
49	Search 1	2026-08-03	(critical appraisal OR overestimation OR inflated OR caution) AND artificial intelligence AND endodontic	none	default	25	20	20
50	Search 3	2026-08-03	(hype OR overpromise OR translational gap OR clinical translation) AND artificial intelligence AND dentistry	2023	default	25	481	25
51	Search 2	2026-08-03	cone beam computed tomography versus periapical radiography detection apical periodontitis sensitivity	none	default	30	2	2
52	Search 2	2026-08-03	Estrela AND cone beam computed tomography AND apical periodontitis	none	default	20	23	20
53	Search 2	2026-08-03	CBCT periapical index CBCT-PAI apical periodontitis	none	default	20	4	4
54	Search 2	2026-08-03	(histological OR histopathological) AND (CBCT OR cone beam) AND periapical lesion AND correlation OR validation	none	default	25	1460115	25
55	Search 2	2026-08-03	differentiation periapical granuloma cyst radiographic histopathology accuracy	none	default	25	3	3
56	Search 2	2026-08-03	(cone beam computed tomography OR CBCT) AND healing AND root canal treatment outcome AND periapical radiograph comparison	none	default	25	10	10
57	Search 2	2026-08-03	("cone-beam computed tomography"[MeSH Terms] OR CBCT[tiab]) AND ("periapical periodontitis"[MeSH Terms] OR "apical periodontitis"[tiab]) AND (periapical radiograph*[tiab] OR intraoral radiograph*[tiab] OR conventional radiograph*[tiab])	none	default	35	119	35
58	Search 2	2026-08-03	CBCT[tiab] AND (detection[tiab] OR diagnosis[tiab]) AND apical periodontitis[tiab] AND (comparison[tiab] OR compared[tiab] OR versus[tiab])	none	default	35	27	27
59	Search 2	2026-08-03	(cadaver*[tiab] OR "ex vivo"[tiab] OR "human jaw"[tiab]) AND (periapical lesion*[tiab] OR apical periodontitis[tiab]) AND (CBCT[tiab] OR cone beam[tiab]) AND (histolog*[tiab] OR gold standard[tiab] OR reference standard[tiab])	none	default	30	5	5
60	Search 2	2026-08-03	(histopatholog*[tiab] OR histolog*[tiab]) AND (apical periodontitis[tiab] OR periapical lesion*[tiab]) AND (radiograph*[tiab] OR CBCT[tiab])	none	default	35	305	35
61	Search 2	2026-08-03	(radicular cyst[tiab] OR periapical granuloma[tiab]) AND (CBCT[tiab] OR radiograph*[tiab]) AND (histopatholog*[tiab] OR diagnos*[tiab]) AND accuracy[tiab]	none	default	30	11	11
62	Search 2	2026-08-03	prevalence[tiab] AND apical periodontitis[tiab] AND (CBCT[tiab] OR cone-beam[tiab]) AND (panoramic[tiab] OR periapical radiograph*[tiab])	none	default	30	9	9
63	Search 2	2026-08-03	(intraobserver[tiab] OR interobserver[tiab] OR observer agreement[tiab] OR reliability[tiab]) AND (periapical radiograph*[tiab] OR periapical index[tiab] OR PAI[tiab]) AND (apical periodontitis[tiab] OR periapical[tiab])	none	default	35	218	35
64	Search 2	2026-08-03	(kappa[tiab] OR agreement[tiab]) AND (radiographic[tiab] OR radiograph*[tiab]) AND (periapical lesion*[tiab] OR periapical status[tiab] OR apical periodontitis[tiab]) AND (examiner*[tiab] OR observer*[tiab] OR dentist*[tiab] OR endodontist*[tiab])	none	default	35	not recorded	0
65	Search 2	2026-08-03	variability[tiab] AND (endodontist*[tiab] OR general dentist*[tiab]) AND radiographic interpretation[tiab]	none	default	25	4	4
66	Search 1	2026-08-03	(deep learning[tiab] OR convolutional neural network*[tiab] OR artificial intelligence[tiab]) AND (periapical radiograph*[tiab] OR intraoral radiograph*[tiab]) AND (periapical lesion*[tiab] OR apical periodontitis[tiab] OR periapical radiolucen*[tiab])	none	default	35	36	35
67	Search 1	2026-08-03	(deep learning[tiab] OR CNN[tiab] OR U-Net[tiab]) AND (CBCT[tiab] OR cone-beam[tiab]) AND (periapical lesion*[tiab] OR apical periodontitis[tiab]) AND (segmentation[tiab] OR detection[tiab])	none	default	30	26	26
68	Search 3	2026-08-03	(external validation[tiab] OR multicent*[tiab] OR generalizab*[tiab]) AND (deep learning[tiab] OR artificial intelligence[tiab]) AND (dental[tiab] OR dentistry[tiab] OR oral radiolog*[tiab])	none	default	30	241	30
69	Search 3	2026-08-03	(CLAIM[tiab] OR "Checklist for Artificial Intelligence in Medical Imaging"[tiab]) AND (dental[tiab] OR dentistry[tiab] OR oral[tiab])	none	default	25	1498	25
70	Search 3	2026-08-03	(MI-CLAIM[tiab] OR CLAIM[tiab] OR STARD-AI[tiab] OR TRIPOD[tiab] OR "TRIPOD+AI"[tiab]) AND (reporting[tiab] OR checklist[tiab]) AND (artificial intelligence[tiab] OR machine learning[tiab])	none	default	30	418	30

No.	Search	Date run (UTC)	Query as executed	Publication-date limit	Sort order	Maximum records requested	Records matching	Records returned
71	Search 3	2026-08-03	(QUADAS-2[tiab] OR QUADAS-AI[tiab] OR PROBAST[tiab] OR "PROBAST+AI"[tiab]) AND (artificial intelligence[tiab] OR machine learning[tiab])	none	default	30	1334	30
72	Search 3	2026-08-03	(risk of bias[tiab] OR methodological quality[tiab] OR reporting quality[tiab]) AND (artificial intelligence[tiab] OR deep learning[tiab]) AND (dental[tiab] OR dentistry[tiab] OR endodontic*[tiab])	none	default	30	125	30
73	Search 3	2026-08-03	(diagnostic accuracy studies[tiab] OR deep learning studies[tiab]) AND (design[tiab] OR quality[tiab] OR bias[tiab]) AND (systematic review[tiab]) AND medical imaging[tiab]	none	default	30	51	30
74	Search 3	2026-08-03	(geographic[tiab] OR geographical[tiab] OR country[tiab] OR countries[tiab]) AND (dataset*[tiab] OR data set*[tiab] OR cohort*[tiab]) AND (deep learning[tiab] OR artificial intelligence[tiab]) AND (imaging[tiab] OR radiolog*[tiab])	none	default	30	365	30
75	Background	2026-08-03	(Global Burden of Disease[tiab]) AND (oral[tiab] OR dental caries[tiab] OR untreated caries[tiab])	none	default	30	264	30
76	Background	2026-08-03	(prevalence[tiab] OR epidemiolog*[tiab]) AND apical periodontitis[tiab] AND (population[tiab] OR global[tiab] OR cross-sectional[tiab])	none	default	30	195	30
77	Search 3	2026-08-03	(low- and middle-income[tiab] OR LMIC[tiab] OR underserved[tiab] OR equity[tiab]) AND (artificial intelligence[tiab] OR teledentistry[tiab]) AND (dental[tiab] OR oral health[tiab])	none	default	30	114	30
78	Search 2	2026-08-03	(observer[tiab] OR agreement[tiab] OR reliability[tiab] OR reproducibility[tiab]) AND (periapical radiograph*[tiab]) AND (diagnos*[tiab] OR interpretation[tiab])	none	default	30	173	30
79	Search 2	2026-08-03	reproducibility of radiographic interpretation endodontic	none	default	20	34	20
80	Search 2	2026-08-03	persistent apical periodontitis[tiab] AND (cone beam[tiab] OR CBCT[tiab]) AND radiograph*[tiab]	none	default	20	10	10
81	Search 2	2026-08-03	(healing[tiab] OR outcome[tiab]) AND root canal treatment[tiab] AND (CBCT[tiab] AND periapical radiograph*[tiab]) AND (success[tiab] OR agreement[tiab])	none	default	25	12	12
82	Search 2	2026-08-03	(magnetic resonance imaging[tiab] OR MRI[tiab]) AND (apical periodontitis[tiab] OR periapical lesion*[tiab]) AND (detection[tiab] OR diagnostic[tiab])	none	default	25	19	19
83	Search 3	2026-08-03	(reporting[tiab] OR checklist[tiab] OR guideline*[tiab]) AND (artificial intelligence[tiab] OR machine learning[tiab]) AND dentistry[tiab]	none	default	30	164	30
84	Search 3	2026-08-03	(quality[tiab] AND (assessment[tiab] OR appraisal[tiab] OR evaluation[tiab])) AND (deep learning[tiab] OR artificial intelligence[tiab]) AND (dental radiograph*[tiab] OR dentistry[tiab] OR oral[tiab]) AND studies[tiab]	none	default	30	122	30
85	Search 3	2026-08-03	(umbrella review[tiab] OR overview of reviews[tiab] OR scoping review[tiab]) AND artificial intelligence[tiab] AND dentistry[tiab]	none	default	25	61	25
86	Search 3	2026-08-03	(caries[tiab] OR periodontal[tiab] OR periapical[tiab]) AND deep learning[tiab] AND (QUADAS[tiab] OR risk of bias[tiab])	none	default	30	44	30
87	Search 1	2026-08-03	(reference standard[tiab] OR ground truth[tiab]) AND (endodontic*[tiab] OR periapical[tiab] OR apical periodontitis[tiab]) AND (artificial intelligence[tiab] OR deep learning[tiab])	none	default	30	59	30
88	Search 3	2026-08-03	(dataset[tiab] OR datasets[tiab]) AND (dental[tiab] OR oral[tiab]) AND (public[tiab] OR open[tiab] OR benchmark[tiab]) AND (deep learning[tiab] OR artificial intelligence[tiab])	none	default	30	129	30
89	Search 3	2026-08-03	(geographic[tiab] OR geographical[tiab]) AND (concentration[tiab] OR distribution[tiab] OR representation[tiab] OR diversity[tiab]) AND (deep learning[tiab] OR artificial intelligence[tiab]) AND (patient data[tiab] OR databases[tiab] OR training data[tiab] OR cohorts[tiab])	none	default	30	65	30
90	Background	2026-08-03	(global burden[tiab] OR worldwide[tiab]) AND (untreated caries[tiab] OR oral disorders[tiab] OR oral diseases[tiab]) AND (prevalence[tiab] OR burden[tiab])	none	default	30	197	30
91	Search 3	2026-08-03	Geographic Distribution of US Cohorts Used to Train Deep Learning Algorithms	none	default	10	1	1
92	Search 3	2026-08-03	sources of bias in artificial intelligence that perpetuate healthcare disparities global review	none	default	15	3	3
93	Search 3	2026-08-03	(health equity[tiab] OR fairness[tiab] OR algorithmic bias[tiab]) AND (medical imaging[tiab] OR radiolog*[tiab]) AND (artificial intelligence[tiab] OR deep learning[tiab])	none	default	25	205	25
94	Background	2026-08-03	(Global Burden of Disease[tiab] OR GBD[tiab]) AND (untreated caries[tiab] OR permanent teeth[tiab] OR oral conditions[tiab]) AND (1990[tiab] OR trends[tiab] OR global[tiab])	none	default	25	53	25
95	Search 1	2026-08-03	(bibliometric[tiab] OR scientometric[tiab] OR research trends[tiab]) AND artificial intelligence[tiab] AND (dentistry[tiab] OR endodontic*[tiab] OR dental[tiab])	none	default	25	45	25
96	Search 2	2026-08-03	(publicly available dataset[tiab] OR open dataset[tiab] OR benchmark dataset[tiab]) AND (panoramic radiograph*[tiab] OR dental radiograph*[tiab] OR periapical radiograph*[tiab])	none	default	25	5	5
97	Background	2026-08-03	apical periodontitis[tiab] AND (untreated[tiab] OR access to care[tiab] OR unmet[tiab] OR low-income[tiab])	none	default	20	112	20
98	Background	2026-08-03	(access to endodontic care[tiab] OR dental workforce[tiab] OR tooth extraction rates[tiab]) AND (inequal*[tiab] OR disparit*[tiab] OR low-income[tiab])	none	default	20	110	20

No.	Search	Date run (UTC)	Query as executed	Publication-date limit	Sort order	Maximum records requested	Records matching	Records returned
99	Search 3	2026-08-03	(circular*[tiab] OR self-referential[tiab] OR tautolog*[tiab] OR "imperfect gold standard"[tiab] OR "imperfect reference"[tiab]) AND (artificial intelligence[tiab] OR deep learning[tiab])	none	default	25	836	25
100	Search 3	2026-08-03	("ground truth"[tiab]) AND (limitation*[tiab] OR problem*[tiab] OR quality[tiab] OR bias[tiab]) AND (deep learning[tiab] OR artificial intelligence[tiab]) AND (radiolog*[tiab] OR imaging[tiab] OR dental[tiab])	none	default	30	658	30
101	Search 3	2026-08-03	(label noise[tiab] OR noisy labels[tiab] OR annotation quality[tiab] OR inter-annotator[tiab]) AND (medical imaging[tiab] OR radiograph*[tiab] OR deep learning[tiab])	none	default	25	263	25
102	Search 2	2026-08-03	(agreement[tiab] OR variability[tiab] OR consistency[tiab]) AND (interpretation[tiab] OR assessment[tiab]) AND periapical[tiab] AND (dentists[tiab] OR endodontists[tiab] OR observers[tiab] OR examiners[tiab])	none	default	30	87	30
103	Search 2	2026-08-03	(periapical index[tiab] OR PAI[tiab]) AND (reliability[tiab] OR calibration[tiab] OR agreement[tiab] OR kappa[tiab])	none	default	25	341	25
104	Search 2	2026-08-03	(interobserver[tiab] OR intraobserver[tiab]) AND (cone beam[tiab] OR CBCT[tiab]) AND (apical periodontitis[tiab] OR periapical[tiab])	none	default	20	65	20
105	Search 2	2026-08-03	endodontic radiographic interpretation observer disagreement decision	none	default	20	0	0
106	Search 2	2026-08-03	Endodontic radiography: who is reading the digital radiograph?	none	default	5	2	2
107	Search 3	2026-08-03	(narrative review[tiab] OR critical review[tiab] OR commentary[tiab] OR editorial[tiab]) AND (artificial intelligence[tiab]) AND (endodontic*[tiab] OR periapical[tiab])	none	default	25	33	25
108	Search 1	2026-08-03	Diagnostics (Basel)[Journal] AND (endodontic*[tiab] OR periapical[tiab]) AND (artificial intelligence[tiab] OR deep learning[tiab])	none	default	25	51	25
109	Search 3	2026-08-04	Checklist for Artificial Intelligence in Medical Imaging CLAIM guide for authors and reviewers	none	relevance	5	5	5
110	Search 3	2026-08-04	Checklist for Artificial Intelligence in Medical Imaging CLAIM 2024 Update	none	relevance	5	2	2
111	Search 3	2026-08-04	QUADAS-2 revised tool quality assessment diagnostic accuracy studies	none	relevance	5	111	5
112	Search 3	2026-08-04	QUADAS-AI quality assessment tool artificial intelligence centred diagnostic test accuracy	none	relevance	5	0	0
113	Search 3	2026-08-04	QUADAS-AI protocol development quality assessment tool artificial intelligence diagnostic accuracy	none	relevance	5	5	5
114	Search 3	2026-08-04	STARD 2015 updated list essential items reporting diagnostic accuracy studies	none	relevance	5	5	5
115	Search 3	2026-08-04	STARD-AI protocol reporting guideline diagnostic accuracy artificial intelligence	none	relevance	5	2	2
116	Search 3	2026-08-04	TRIPOD+AI statement reporting clinical prediction models machine learning	none	relevance	5	11	5
117	Search 3	2026-08-04	PRIDASE guidelines reporting in vitro diagnostic accuracy studies endodontics	none	relevance	5	0	0
118	Search 3	2026-08-04	SANRA scale for the quality assessment of narrative review articles	none	relevance	5	86	5
119	Search 3	2026-08-04	STARD-AI checklist statement reporting diagnostic accuracy artificial intelligence 2026	none	publication date	6	not recorded	0
120	Search 3	2026-08-04	STARD-AI checklist statement reporting diagnostic accuracy artificial intelligence	none	publication date	8	1	1
121	Search 3	2026-08-04	PRIDASE guidelines reporting diagnostic accuracy studies endodontics	none	publication date	8	4	4
122	Search 3	2026-08-04	QUADAS-AI tool development Delphi	none	publication date	8	1	1
123	Search 3	2026-08-04	reference standard artificial intelligence dentistry ground truth critique	none	publication date	8	1	1
124	Search 3	2026-08-04	ground truth quality artificial intelligence dental radiography annotation reliability	none	publication date	8	0	0
125	Search 3	2026-08-04	(ground truth OR reference standard) AND (artificial intelligence OR deep learning) AND (dentistry OR dental OR endodontic OR radiograph)	2025/01/01	publication date	25	1370	25
126	Search 3	2026-08-04	imperfect reference standard artificial intelligence diagnostic accuracy bias overestimation	2025/01/01	publication date	25	0	0
127	Search 3	2026-08-04	imperfect reference standard bias correction sensitivity specificity latent class no gold standard	2025/01/01	publication date	25	0	0
128	Search 3	2026-08-04	label noise annotation quality medical imaging deep learning performance ceiling	2025/01/01	publication date	25	0	0
129	Search 2	2026-08-04	(apical periodontitis OR periapical) AND (histopathology OR histological) AND (cone beam computed tomography OR radiograph) AND diagnostic accuracy	2025/01/01	publication date	25	115	25
130	Search 2	2026-08-04	(apical periodontitis OR periapical lesion) AND (interobserver OR inter-examiner OR observer agreement OR kappa) AND radiograph	2025/01/01	publication date	25	157	25
131	Search 3	2026-08-04	(artificial intelligence OR deep learning) AND (apical periodontitis OR periapical lesion) AND cone beam computed tomography reference standard	2025/01/01	publication date	25	8	8

No.	Search	Date run (UTC)	Query as executed	Publication-date limit	Sort order	Maximum records requested	Records matching	Records returned
132	Search 3	2026-08-04	circularity artificial intelligence validation expert labels medical imaging accuracy inflated	2025/06/01	publication date	15	0	0
133	Search 3	2026-08-04	(ground truth OR reference standard) problem artificial intelligence radiology critique epistemic	2025/06/01	publication date	15	0	0
134	Search 3	2026-08-04	endodontics artificial intelligence editorial critique methodology rigour	2025/06/01	publication date	15	0	0
135	Search 3	2026-08-04	dental artificial intelligence annotation ground truth reliability limitation review 2026	2025/06/01	publication date	15	1	1
136	Search 3	2026-08-04	imperfect gold standard bias diagnostic accuracy artificial intelligence correction	2025/06/01	publication date	15	0	0
137	Search 3	2026-08-04	reference standard hierarchy diagnostic accuracy clinical question imaging validation framework	2025/06/01	publication date	15	1	1
138	Search 2	2026-08-14	cone beam computed tomography periapical radiograph periapical status irreversible pulpitis vital pulp prevalence	2016/01/01	relevance	25	0	0
139	Search 2	2026-08-14	CBCT versus periapical radiography detection apical periodontitis untreated teeth clinical comparison	2016/01/01	relevance	25	0	0
140	Search 2	2026-08-14	cone beam computed tomography periapical radiography necrotic pulp roots apical periodontitis detection	2016/01/01	relevance	25	1	1
141	Search 2	2026-08-14	ex vivo accuracy cone beam CT intraoral digital radiography detection small periapical lesions jaws	2016/01/01	relevance	25	1	1
142	Search 2	2026-08-14	artificial periapical lesion detection accuracy CBCT digital radiography ex vivo mandible defect size	2016/01/01	relevance	25	0	0
143	Search 2	2026-08-14	periapical index PAI interobserver agreement kappa observers root filled teeth index systems comparison	2016/01/01	relevance	25	1	1
144	Search 2	2026-08-14	periapical index reliability observer agreement radiographic periapical assessment kappa	2016/01/01	relevance	25	5	5
145	Background	2026-08-14	global burden of disease oral disorders untreated caries prevalence billion	2016/01/01	relevance	25	15	15
146	Background	2026-08-14	Global Burden of Disease 2021 oral conditions untreated dental caries permanent teeth prevalence	2016/01/01	relevance	25	7	7
147	Search 3	2026-08-14	evaluating diagnostic accuracy multiple reference standards bias	2016/01/01	relevance	25	88	25
148	Search 2	2026-08-14	CBCT periapical radiography apical periodontitis detection prevalence teeth	2016/01/01	relevance	40	20	20
149	Search 2	2026-08-14	cone beam computed tomography periapical radiographs apical periodontitis untreated teeth	2016/01/01	relevance	40	10	10
150	Search 2	2026-08-14	ex vivo periapical lesion detection cone beam computed tomography accuracy	2016/01/01	relevance	40	9	9
151	Search 2	2026-08-14	periapical lesion size detection accuracy CBCT radiography simulated lesions	2016/01/01	relevance	40	3	3
152	Search 2	2026-08-14	periapical index observer agreement	2016/01/01	relevance	40	26	26
153	Search 2	2026-08-14	periapical index systems comparison radiographic assessment periapical health	2016/01/01	relevance	40	3	3
154	Background	2026-08-14	global burden oral diseases 2021 caries prevalence	2016/01/01	relevance	40	32	32
155	Background	2026-08-14	untreated caries global prevalence systematic analysis burden	2016/01/01	relevance	40	13	13
156	Search 2	2026-08-14	interobserver agreement periapical radiographs root filled teeth kappa scoring	2016/01/01	relevance	40	3	3
157	Search 2	2026-08-14	Accuracy of Periapical Radiography and Cone-Beam Computed Tomography Scans in Diagnosing Apical Periodontitis Using Histopathological Findings as a Gold Standard	none	default	5	0	0
158	Search 2	2026-08-14	Accuracy of cone beam computed tomography and panoramic and periapical radiography for detection of apical periodontitis Estrela	none	default	5	1	1
159	Search 2	2026-08-14	de Paula-Silva periapical radiography cone-beam computed tomography apical periodontitis histopathological gold standard dog	none	default	8	1	1
160	Search 2	2026-08-14	apical periodontitis visible cone beam computed tomography missed periapical radiographs	2016/01/01	relevance	30	1	1
161	Search 2	2026-08-14	periapical status vital pulp cone beam computed tomography radiograph roots comparison prospective	2016/01/01	relevance	30	0	0
162	Search 2	2026-08-14	CBCT periapical radiography paired comparison roots lesion detection clinical study 2020	2016/01/01	relevance	30	0	0
163	Search 2	2026-08-14	periapical index PAI observer agreement artificial intelligence deep learning kappa radiographs	2016/01/01	relevance	30	0	0
164	Search 2	2026-08-14	reliability periapical index scoring radiographs observers weighted kappa	2016/01/01	relevance	30	0	0
165	Search 2	2026-08-14	agreement among observers periapical index CBCT PAI scoring reproducibility	2016/01/01	relevance	30	0	0
166	Background	2026-08-14	Global Burden of Disease Study 2019 oral conditions untreated caries permanent teeth	2016/01/01	relevance	30	6	6
167	Background	2026-08-14	global oral health burden 2022 untreated caries 2 billion people	2016/01/01	relevance	30	0	0
168	Background	2026-08-14	oral disorders global burden 1990 2019 systematic analysis	2016/01/01	relevance	30	22	22
169	Background	2026-08-14	Bernabe global burden oral conditions	2016/01/01	relevance	30	21	21
170	Background	2026-08-14	trends burden oral conditions 1990 2017 systematic analysis	2016/01/01	relevance	40	15	15
171	Background	2026-08-14	burden oral conditions systematic analysis Global Burden Disease	2016/01/01	relevance	40	204	40

No.	Search	Date run (UTC)	Query as executed	Publication-date limit	Sort order	Maximum records requested	Records matching	Records returned
172	Background	2026-08-14	untreated caries permanent teeth prevalence global	2016/01/01	relevance	40	34	34
173	Search 2	2026-08-14	periapical index reliability	2016/01/01	relevance	40	54	40
174	Search 2	2026-08-14	periapical index agreement observers	2016/01/01	relevance	40	26	26
175	Search 2	2026-08-14	radiographic periapical index scoring agreement	2016/01/01	relevance	40	17	17
176	Search 2	2026-08-14	periapical radiographs cone beam computed tomography paired teeth lesion detection difference	2016/01/01	relevance	40	4	4
177	Search 2	2026-08-14	simulated periapical lesions detection accuracy periapical radiography cone beam	2016/01/01	relevance	40	15	15
178	Background	2026-08-14	Global Regional National Levels Trends Burden Oral Conditions 1990 2017	none	relevance	30	11	11
179	Background	2026-08-14	oral diseases burden Global Burden of Disease Study 2021 caries edentulism periodontitis	none	relevance	30	13	13
180	Background	2026-08-14	global oral health status report burden oral diseases 3.5 billion	none	relevance	30	0	0
181	Background	2026-08-14	untreated caries permanent teeth 2 billion prevalent condition	none	relevance	30	8	8
182	Search 2	2026-08-14	Periapical Index screening endodontic disease reliability accuracy	none	relevance	30	2	2
183	Search 2	2026-08-14	deep learning periapical index classification interobserver agreement dentists	none	relevance	30	0	0
184	Search 2	2026-08-14	calibration observers periapical index radiographic scoring endodontics agreement study	none	relevance	30	1	1
185	Search 2	2026-08-14	comparison index systems radiographic periapical assessment observers	none	relevance	30	2	2
186	Search 2	2026-08-14	periapical index interobserver agreement periapical radiographs observers root filled	2016/01/01	relevance	40	1	1
187	Search 2	2026-08-14	Strindberg criteria periapical index agreement observers radiographic	2016/01/01	relevance	40	0	0
188	Search 2	2026-08-14	multiple observers periapical radiographs periapical index kappa agreement 2020	2016/01/01	relevance	40	0	0
189	Search 2	2026-08-14	artificial intelligence periapical index scoring agreement observers panoramic radiographs kappa	2016/01/01	relevance	40	1	1
190	Search 2	2026-08-14	artificial periapical bone defects 1 mm 2 mm detection accuracy radiography cone beam ex vivo	2016/01/01	relevance	40	0	0
191	Search 2	2026-08-14	diagnostic accuracy periapical radiography cone beam computed tomography artificial lesions observers sensitivity specificity	2016/01/01	relevance	40	5	5
192	Search 2	2026-08-14	prospective comparison periapical radiography cone beam computed tomography apical periodontitis roots patients	2016/01/01	relevance	40	1	1
193	Search 2	2026-08-14	lesions detected only on CBCT not periapical radiograph proportion teeth	2016/01/01	relevance	40	66	40
194	Search 2	2026-08-14	periapical index interobserver kappa observers 2D radiographs agreement periapical status assessment	2016/01/01	relevance	30	0	0
195	Search 2	2026-08-14	convolutional neural network periapical index panoramic radiograph observer agreement kappa periapical status	2016/01/01	relevance	30	0	0
196	Search 2	2026-08-14	proportion apical periodontitis detected CBCT undetected periapical radiograph teeth study	2016/01/01	relevance	30	0	0
197	Background	2026-08-14	untreated caries permanent teeth 2021 global prevalence cases billion GBD	2016/01/01	relevance	30	1	1
198	Background	2026-08-14	untreated caries permanent teeth 2021 number of cases global prevalent cases GBD 2021	2022/01/01	relevance	20	2	2
199	Background	2026-08-14	2.5 billion untreated caries permanent teeth global	2022/01/01	relevance	20	0	0
200	Background	2026-08-14	global prevalence untreated caries permanent teeth cases 2021 systematic analysis burden	2022/01/01	relevance	20	1	1
201	Background	2026-08-14	WHO global oral health status report oral diseases affect 3.5 billion people	2022/01/01	relevance	20	0	0
202	Search 2	2026-08-14	Radiographic and histologic periapical findings of root canal treated teeth in cadaver	none	default	5	2	2
203	Search 2	2026-08-14	apical periodontitis AND (histopathology OR histological) AND (sensitivity OR specificity OR diagnostic accuracy) AND radiograph*	none	relevance	40	87	40
204	Search 2	2026-08-14	(cadaver OR ex vivo) AND periapical lesion AND (cone beam OR CBCT OR radiograph) AND accuracy	none	relevance	40	21	21
205	Search 2	2026-08-14	periapical lesion detection AND (meta-analysis OR systematic review) AND diagnostic accuracy	none	relevance	40	21	21
206	Search 2	2026-08-14	periapical lesion AND surgical findings AND radiographic diagnosis accuracy	none	relevance	40	18	18
207	Search 2	2026-08-14	CBCT reference standard AND periapical radiograph AND sensitivity apical periodontitis	none	relevance	40	6	6
208	Search 2	2026-08-14	lesion size AND detection AND periapical radiograph AND (cortical OR cancellous)	none	relevance	40	4	4
209	Search 2	2026-08-14	simulated periapical bone lesion AND radiographic detection AND size	none	relevance	40	5	5
210	Search 2	2026-08-14	cancellous bone lesion radiographic detection mandible cortical plate	none	relevance	40	3	3
211	Search 2	2026-08-14	periapical lesion volume AND CBCT AND detectability radiograph	none	relevance	40	12	12
212	Search 2	2026-08-14	minimum detectable bone lesion size radiography jaw	none	relevance	40	1	1
213	Search 2	2026-08-14	anatomical region AND periapical radiolucency detection AND (maxilla OR mandible) accuracy	none	relevance	40	1	1
214	Search 2	2026-08-14	maxillary sinus OR zygomatic AND periapical lesion detection radiograph superimposition	none	relevance	40	0	0
215	Search 2	2026-08-14	observer agreement AND periapical lesion detection AND kappa radiograph	none	relevance	40	16	16
216	Search 2	2026-08-14	interobserver reliability radiographic diagnosis periapical bone loss kappa	none	relevance	40	1	1
217	Search 2	2026-08-14	apical periodontitis AND diagnostic accuracy AND imaging	2020	relevance	40	141	40
218	Search 2	2026-08-14	periapical lesion AND (sensitivity AND specificity) AND (CBCT OR periapical radiography)	2020	relevance	40	185	40
219	Search 2	2026-08-14	periapical granuloma AND radicular cyst AND radiographic differentiation histopathology	none	relevance	40	19	19

No.	Search	Date run (UTC)	Query as executed	Publication-date limit	Sort order	Maximum records requested	Records matching	Records returned
220	Search 2	2026-08-14	radiographic AND histopathological correlation periapical lesions	none	relevance	40	25	25
221	Search 2	2026-08-14	CBCT AND periapical lesion AND histopathological diagnosis granuloma cyst	none	relevance	40	14	14
222	Search 2	2026-08-14	Bender Seltzer roentgenographic bone lesion	none	relevance	40	2	2
223	Search 2	2026-08-14	artificial bone lesion radiographic detection jaw	none	relevance	40	23	23
224	Search 2	2026-08-14	chemically created osseous lesion radiographic detection	none	relevance	40	4	4
225	Search 2	2026-08-14	experimental bone defect radiographic visibility cortical bone	none	relevance	40	3	3
226	Search 2	2026-08-14	bone lesion depth AND radiographic detection AND periapical	none	relevance	40	10	10
227	Search 2	2026-08-14	lesion size influence CBCT detection periapical	none	relevance	40	4	4
228	Search 2	2026-08-14	lesion diameter sensitivity radiographic detection periapical lesion	none	relevance	40	5	5
229	Search 2	2026-08-14	medullary bone lesion radiograph not detectable cortical involvement	none	relevance	40	195	40
230	Search 2	2026-08-14	Wenzel periapical bone lesion detection observer	none	relevance	40	1	1
231	Search 2	2026-08-14	cortical plate perforation radiographic appearance periapical lesion	none	relevance	40	1	1
232	Search 2	2026-08-14	periapical lesion detection maxilla mandible difference radiographic sensitivity	none	relevance	40	5	5
233	Search 2	2026-08-14	anatomical region diagnostic performance periapical radiolucency deep learning	none	relevance	40	1	1
234	Search 2	2026-08-14	tooth type region periapical radiolucency detection accuracy panoramic	none	relevance	40	0	0
235	Search 2	2026-08-14	maxillary sinus floor periapical lesion radiographic detection difficulty	none	relevance	40	0	0
236	Search 2	2026-08-14	agreement detection periapical radiolucency observers cone beam computed tomography kappa	none	relevance	40	3	3
237	Search 2	2026-08-14	observer variation radiographic periapical diagnosis	none	relevance	40	211	40
238	Search 2	2026-08-14	panoramic radiograph periapical lesion observer agreement	none	relevance	40	10	10
239	Search 2	2026-08-14	pig jaw OR sheep mandible periapical lesion detection accuracy CBCT	none	relevance	40	1689	40
240	Search 2	2026-08-14	cone beam computed tomography apical periodontitis diagnostic test accuracy meta-analysis	none	relevance	40	1	1
241	Search 2	2026-08-14	periapical biopsy histopathologic diagnosis radiographic impression agreement	none	relevance	40	0	0
242	Search 2	2026-08-14	cyst granuloma lesion size radiographic distinction periapical	none	relevance	40	2	2
243	Search 2	2026-08-14	Bender roentgenographic interpretation experimental bone lesions	none	relevance	25	0	0
244	Search 2	2026-08-14	to what extent does bone destruction have to occur before radiographic detection	none	relevance	25	7	7
245	Search 2	2026-08-14	incisive canal mimic periapical radiolucency diagnosis	none	relevance	25	2	2
246	Search 2	2026-08-14	periapical lesion volume mm3 threshold detection CBCT sensitivity	none	relevance	25	0	0
247	Search 2	2026-08-14	lesion size stratified sensitivity artificial intelligence periapical radiolucency detection	none	relevance	25	0	0
248	Search 2	2026-08-14	cortical bone involvement necessary radiographic detection periapical lesion detectability	none	relevance	25	0	0
249	Search 2	2026-08-14	periapical lesion detection radiographic sensitivity trabecular bone loss threshold	none	relevance	25	0	0
250	Search 2	2026-08-14	detection periapical lesions maxillary posterior teeth difficulty radiograph accuracy	none	relevance	25	0	0
251	Search 2	2026-08-14	agreement presence absence periapical lesion two observers periapical radiograph kappa detection	none	relevance	25	0	0
252	Search 2	2026-08-14	cannot differentiate periapical granuloma from cyst radiographically size criterion	none	relevance	25	0	0
253	Search 2	2026-08-14	surgical exploration reference standard periapical lesion imaging accuracy	none	relevance	25	0	0
254	Search 2	2026-08-14	cadaver jaw histology reference apical periodontitis CBCT accuracy blinded	none	relevance	25	0	0
255	Search 2	2026-08-14	Roentgenographic and direct observation of experimental lesions in bone	none	relevance	10	2	2
256	Search 2	2026-08-14	factors influencing the radiographic appearance of bony lesions	none	relevance	10	8	8
257	Search 2	2026-09-18	apical periodontitis histopathology radiograph cadaver sensitivity	2020–2026 (publication date)	default	15	1	1
258	Search 2	2026-09-18	(apical periodontitis OR periapical lesion*) AND (histolog* OR histopatholog*) AND (radiograph* OR CBCT) AND (accuracy OR sensitivity) AND (cadaver* OR "reference standard" OR "gold standard")	2020–2026 (publication date)	default	12	5	5
259	Search 2	2026-09-18	(periapical lesion* OR radicular cyst OR periapical granuloma) AND histopatholog* AND radiograph* AND (agreement OR correspond* OR accuracy OR diagnosis)	2020–2026 (publication date)	default	12	115	12
260	Search 2	2026-09-18	(periapical OR apical) AND (lesion size OR defect size OR simulated lesion* OR artificial lesion*) AND (radiograph* OR CBCT) AND (detect* OR sensitivity) AND (cortical OR cancellous OR millimet*)	2020–2026 (publication date)	default	12	not recorded	not recorded
261	Search 2	2026-09-18	(apical periodontitis OR periapical) AND "cone-beam" AND periapical radiograph* AND (prevalence OR sensitivity OR detection) AND (necrotic OR irreversible pulpitis OR root-filled OR root canal treated)	2020–2026 (publication date)	default	12	not recorded	not recorded
262	Search 2	2026-09-18	(periapical OR apical periodontitis) AND radiograph* AND (interobserver OR inter-observer OR interexaminer OR intraobserver OR kappa OR "observer agreement" OR reliability)	2020–2026 (publication date)	default	12	not recorded	not recorded
263	Search 2	2026-09-18	"periapical index" AND (validation OR reliability OR histolog*)	2020–2026 (publication date)	default	12	not recorded	not recorded
264	Search 3	2026-09-18	"imperfect reference standard" OR "imperfect gold standard" OR ("dependent errors" AND "reference standard") OR ("conditional dependence" AND diagnostic accuracy)	2020–2026 (publication date)	default	12	not recorded	not recorded

No.	Search	Date run (UTC)	Query as executed	Publication-date limit	Sort order	Maximum records requested	Records matching	Records returned
265	Search 3	2026-09-18	(QUADAS-AI OR "STARD-AI" OR "QUADAS-2" OR "STARD 2015") AND (diagnostic accuracy) AND (update OR extension OR tool)	2020–2026 (publication date)	default	12	not recorded	not recorded
266	Search 2	2026-09-18	(radicular cyst OR periapical cyst) AND (granuloma) AND radiograph* AND histopatholog* AND (differentiat* OR distinguish*)	2020–2026 (publication date)	default	12	not recorded	not recorded
267	Search 2	2026-09-18	ultrasonography AND periapical lesion* AND histopatholog*	2020–2026 (publication date)	default	12	not recorded	not recorded
268	Search 2	2026-09-18	(Bender AND Seltzer) OR ("cancellous bone" AND periapical AND radiograph* AND detect*)	2020–2026 (publication date)	default	12	not recorded	not recorded
269	Search 2	2026-09-18	(periapical radiograph* OR intraoral radiograph*) AND (apical periodontitis OR periapical lesion* OR periapical status) AND (interobserver OR inter-observer OR interexaminer OR intraobserver) AND (kappa OR agreement) NOT (deep learning OR artificial intelligence OR neural network)	2020–2026 (publication date)	default	12	20	12
270	Search 2	2026-09-18	(periapical OR apical periodontitis) AND radiograph* AND observer* AND calibration AND (agreement OR reliability)	2020–2026 (publication date)	default	12	not recorded	not recorded
271	Search 2	2026-09-18	(apical periodontitis OR periapical radiolucenc*) AND (cone-beam OR CBCT) AND (periapical radiograph* OR intraoral radiograph*) AND (paired OR same teeth OR compared) AND (prevalence OR detected) NOT (deep learning OR artificial intelligence)	2020–2026 (publication date)	default	12	not recorded	not recorded
272	Search 2	2026-09-18	(cadaver* OR post-mortem OR autopsy) AND (apical periodontitis OR periapical) AND (radiograph* OR CBCT) AND (histolog* OR histopatholog*)	2020–2026 (publication date)	default	12	not recorded	not recorded
273	Search 2	2026-09-18	("periapical index" OR PAI) AND (Orstavik OR Ørstavik) AND (reliability OR validation OR reproducibility) NOT (deep learning OR artificial intelligence)	2020–2026 (publication date)	default	12	not recorded	not recorded
274	Search 3	2026-09-18	("imperfect reference standard" OR "imperfect gold standard" OR "missing reference standard") AND (review OR tutorial OR guidance OR overview) AND (diagnostic accuracy)	2020–2026 (publication date)	default	15	10	10
275	Search 3	2026-09-18	("incorporation bias" OR "reference standard bias" OR "verification bias") AND (diagnostic accuracy) AND (review OR taxonomy OR sources of bias)	2020–2026 (publication date)	default	15	47	15
276	Search 2	2026-09-18	apical periodontitis cadaver histopathology radiograph sensitivity	none	default	3	not recorded	0
