# Supplementary material for: Reference Standards in Artificial Intelligence Detection of Apical Periodontitis: A Narrative Review of What Reported Accuracy Measures
Source: Diagnostics (Basel). 2026 Oct 6;16(19):3225. doi: 10.3390/diagnostics16193225 (PMC13664220; doi:10.3390/diagnostics16193225)
Supplement: Supplementary file 1 [file diagnostics-16-03225-s001.zip › 05_Note_S1_Scenario_derivation.pdf]

Supplementary Note S1. Derivation of the scenario calculation.

This note sets out in full the calculation summarised in the section "Ceiling Propagation" and plotted in Figure 2. Reference numbers follow the main manuscript's reference list.

S1.1 Exact decomposition

The gap between concordance and detection can be written out exactly. Let D denote true disease, H the human reading of the index image that serves as the label, and M the model's output. Sensitivity against disease decomposes exactly as

$$Se_{true} = P(M=1 \mid D=1) = P(M=1 \mid H=1, D=1)\, Se_H + P(M=1 \mid H=0, D=1)\,(1 - Se_H),$$

where $Se_H = P(H=1 \mid D=1)$ is the sensitivity of the human 2D reading against an independent reference. The two probabilities on the right are conditioned on disease, and a same-image design does not observe them. What it observes is conditioned on the label: $Se_{AI} = P(M=1 \mid H=1)$ and $1 - Sp_{AI} = P(M=1 \mid H=0)$. Figure 2 therefore fixes the unobserved quantities by two assumptions. First, among true cases the reader detected, the model is positive at the same rate as among reader-positives generally, $P(M=1 \mid H=1, D=1) = Se_{AI}$. Second, among true cases the reader missed, the model is positive at the same rate as among reader-negatives generally, $P(M=1 \mid H=0, D=1) = 1 - Sp_{AI}$. Together they say that the model's output depends on disease only through the human label: a model that has learned to reproduce its annotators and carries no information about the lesion beyond what their reading carries. Under these two assumptions,

$$Se_{true} = Se_{AI}\, Se_H + (1 - Sp_{AI})(1 - Se_H).$$

S1.2 What the assumptions do, and what departures from them mean

This is a scenario, not a bound and not a property of any particular model. The first term counts true cases the reader detected and the model agreed on; the second, true cases the reader missed and the model flagged anyway, at its false-positive rate. A model that finds part of what the reader missed has $P(M=1 \mid H=0, D=1)$ above $1 - Sp_{AI}$ and a higher sensitivity against disease than the scenario returns; a model that shares the reader's blind spots has a lower one. Neither can be estimated from data reported against the reader's label. The scenario answers one question: if a model behaved as its label does, what would a reported sensitivity say about disease?

S1.3 The dependence parameter λ

The first departure — a model sharing the reader's blind spots — is the one panel b examines. Introducing a single dependence parameter $\lambda \in [0, 1]$, the share of the human label's own false negatives on which the model is also negative, gives

$$Se_{true} = Se_{AI}\, Se_H + (1 - \lambda)(1 - Sp_{AI})(1 - Se_H),$$

so $\lambda = 0$ recovers the scenario of panel a and $\lambda = 1$ a fully shared blind spot. The additional reduction, $\lambda(1 - Sp_{AI})(1 - Se_H)$, is linear in λ and largest where Se_H is smallest: at $\lambda = 1$ and $Sp_{AI} = 0.98$ it reaches 1.7 percentage points at $Se_H = 0.16$, and 8.4 points at specificity 0.90 (Figure 2b). The second departure, a model detecting part of what the reader missed, is not plotted, because nothing in the published record of a same-image study allows its size to be guessed; it is the reason the scenario is not a bound.

S1.4 Sourcing of the inputs

The scenario's two inputs are taken from the AI literature itself, specificity first. Set $Sp_{AI} = 0.98$, the pooled specificity for deep-learning detection of periapical radiolucencies on panoramic radiographs [7]

— a value whose confidence interval runs from 0.87 to 1.00, softer than it looks. A second synthesis could not serve as an independent check. Across 24 studies, 4 of them pooled, it reports sensitivity 0.94 (95% CI 0.90–0.96) at specificity 0.96 (95% CI 0.91–0.98) [68]; its reference standard is not stated, which places its inputs inside the arrangement examined here rather than outside it, and makes it a worked example of the scenario's inputs rather than an independent benchmark.

The calculation takes $Se_{\mathrm{AI}} = 0.90$ as its input, a value near the middle of the range the audited same-image studies report. What a sensitivity against such a label stands for can be read off one recent evaluation against same-image consensus. Across 120 periapical radiographs — 60 with apical periodontitis, 60 healthy — referenced to two experienced clinicians' consensus on the two-dimensional image without CBCT confirmation, at a reference interobserver kappa of 0.88, three general-purpose multimodal models returned sensitivity 95.00% at specificity 13.33%, 20.00% at 98.33%, and 95.00% at 48.33% [69]. Two of the three sit near 0.95 and one far below it; the example shows the range a same-image sensitivity can take, not a typical value, and each figure is agreement with the two readers rather than detection of disease. At that input the scenario returns sensitivities against disease of 0.161 at $Se_{\mathrm{H}} = 0.16$, 0.258 at 0.27, 0.354 at 0.38 and 0.504 at 0.55, so that the reported figure exceeds the scenario value by factors of 5.60, 3.49, 2.54 and 1.79 (Figure 2a, where marker labels are rounded to two decimal places).

The four Se_{H} values are not a graded scale. Three (0.16, 0.27, 0.38) come from a 67-tooth human cadaver series against histopathology [1,14] and 0.55 from a clinical series using CBCT rather than tissue as reference [32]; they differ in reference standard and imaged population. What they share is a reference other than the index radiograph, the only property the calculation requires. They are an illustrative range of plausible label sensitivities rather than a dose-response curve, and the point holds for both panels: Figure 2 spans the plausible quality of a same-image label, does not describe any single population, and the curve between the plotted points has no empirical referent. Each point answers what a reported sensitivity would imply, under the scenario, if the label were that good.

S1.5 Relation to the imperfect-reference-standard literature

The direction of such a gap has been worked out formally for a neighbouring problem. Walter et al. [70] treat a related but distinct problem: a test evaluated against an imperfect reference standard, where the question is how the measured figure relates to the test's true accuracy under a specified dependence between the two sets of errors. They show that the apparent sensitivity can be biased in either direction, the sign depending on prevalence, the true operating characteristics of test and reference, and the covariance of their errors among cases and among non-cases; when the errors are independent, apparent sensitivity always falls below the true value. That paper is cited here for what it shows — that direction is a property of the dependence structure rather than of imperfection as such — and not as a derivation of Equation (1) of the main text, whose estimand is different. The starting points differ: Walter et al. begin from a known model and reference and ask what the comparison will report; Figure 2 begins from a reported figure and asks what it would mean about disease if the model behaved as its label does. The two settings need not agree in sign. Under independent errors, which is Walter's baseline case, the reported figure understates sensitivity against disease; under the imitation scenario of Figure 2, it overstates it. Which case describes a given model depends on the dependence between its errors and the reader's, and that dependence cannot be identified from data reported against the reader's label, since λ cannot be estimated without observing cases the reference reader missed.

S1.6 What the calculation is not

The calculation is a scenario, not a bound, not a correction factor, and not an estimate of any published model's accuracy against disease. It answers one conditional question: if a model reproduced its reader, what would a sensitivity reported against that reader's label imply about disease? Because the two conditional probabilities the design does not observe cannot be estimated from same-image data, no

numerical correction to any reported figure follows from it, and the direction of the gap for a real model is not identified.
